# Design, Synthesis, In Vitro and In Silico Evaluation of Triazole–Benzimidazole Hybrid Derivatives as Potential Inhibitors of EGFR, EGFR-L858R, and EGFR-L858R/T790M

**DOI:** 10.3390/molecules31193433

**Published:** 2026-09-27

**Authors:** Harun Uslu, Arzu Hıdır, Esra Yumuşak, Derya Osmaniye, Bünyamin Göktaş, Yusuf Özkay, Zafer Asım Kaplancıklı

**Affiliations:** 1 Department of Pharmaceutical Chemistry, Faculty of Pharmacy, Fırat University, 23119 Elazığ, Türkiye; 2 Department of Pharmaceutical Chemistry, Faculty of Pharmacy, Anadolu University, 26470 Eskişehir, Türkiye; 3 Department of Pharmaceutical Chemistry, The Institute of Graduate Education, Anadolu University, 26470 Eskişehir, Türkiye; 4 Faculty of Pharmacy, Anadolu University, 26470 Eskişehir, Türkiye; 5 Central Research Laboratory, Faculty of Pharmacy, Anadolu University, 26470 Eskişehir, Türkiye

**Keywords:** mutant EGFR, EGFR inhibitors, triazole, benzimidazole, ADME, molecular docking, molecular dynamics

## Abstract

Epidermal growth factor receptor (EGFR) has become one of the most important molecular targets for the development of anticancer agents because of its critical role in regulating cell proliferation, differentiation, and survival. In the present study, a novel series of benzimidazole–triazole derivatives were designed by incorporating pharmacophoric features of clinically used EGFR inhibitors, synthesized, characterized, and evaluated for their anticancer potential. Cytotoxicity studies against the A549 lung cancer cell line identified 2-((4-Ethyl-5-(2-(4-hydroxyphenyl)-1*H*-benzo[*d*]imidazol-6-yl)-4*H*-1,2,4-triazol-3-yl)thio)-*N*-methylacetamide, 2-((4-Ethyl-5-(2-(4-hydroxyphenyl)-1*H*-benzo[*d*]imidazol-6-yl)-4*H*-1,2,4-triazol-3-yl)thio)-*N*-(hydroxymethyl)acetamide, 2-((4-Ethyl-5-(2-(4-hydroxyphenyl)-1*H*-benzo[d]imidazol-6-yl)-4*H*-1,2,4-triazol-3-yl)thio)-*N*-methoxy-*N*-methylacetamide, and 2-((4-Ethyl-5-(2-(4-hydroxyphenyl)-1*H*-benzo[*d*]imidazol-6-yl)-4*H*-1,2,4-triazol-3-yl)thio)-*N*-phenylacetamide as active derivatives, with compound **5a** exhibiting the highest antiproliferative activity (IC_50_ = 6.014 ± 0.758 µM), and relatively low cytotoxicity toward NIH/3T3 cells. Based on these findings, 2-((4-Ethyl-5-(2-(4-hydroxyphenyl)-1*H*-benzo[*d*]imidazol-6-yl)-4*H*-1,2,4-triazol-3-yl)thio)-*N*-methylacetamide was further evaluated against EGFR and its clinically relevant mutant forms, EGFR-L858R and EGFR-L858R/T790M. Retrospective in silico ADME analysis indicated that the synthesized compounds generally possessed favorable drug-like properties, with 2-((4-Ethyl-5-(2-(4-hydroxyphenyl)-1*H*-benzo[*d*]imidazol-6-yl)-4*H*-1,2,4-triazol-3-yl)thio)-*N*-methylacetamide showing the most favorable overall drug-likeness profile among the series. Molecular docking and molecular dynamics studies revealed that compound 2-((4-Ethyl-5-(2-(4-hydroxyphenyl)-1*H*-benzo[*d*]imidazol-6-yl)-4*H*-1,2,4-triazol-3-yl)thio)-*N*-methylacetamide formed stable interactions with key residues in the ATP-binding site of unmutated EGFR, supporting the in vitro inhibition results. Overall, synthesized benzimidazole–triazole derivatives may be promising precursor compounds for the development of novel EGFR inhibitors, particularly against unmutated EGFR cancer cells.

## 1. Introduction

According to the World Health Organization (WHO) and its cancer agency, the International Agency for Research on Cancer (IARC), an estimated 9.8 million cancer-related deaths occurred worldwide in 2024 [1]. Sadly, one in five people will develop cancer during their lifetime. Cancer cells exhibit many different changes, including irregular cell division. This irregular cell division is caused by abnormalities in intracellular signaling pathways. Today, receptor tyrosine kinases (RTKs), which play important roles in these signaling pathways, have become a key target in the design of anticancer agents to correct these abnormal signaling pathways [2,3,4].

Tyrosine kinases are a family of protein kinase enzymes that transfer phosphate groups from ATP, phosphorylate tyrosine residues in cellular proteins, and attach phosphates to extra amino acids such as serine and threonine. The epidermal growth factor receptor (EGFR), a tyrosine kinase signaling component of the erythroblastic leukemia viral oncogene (ErbB) family, is a transmembrane glycoprotein whose ATP-dependent activation mediates proliferation, differentiation, and survival signaling [2,5].

EGFR inhibitors are used to treat many common malignancies, including lung cancer, which is a major cause of cancer-related deaths worldwide. One of the most important factors leading to the discontinuation of EGFR inhibitor treatment is EGFR mutations. Point mutations in exon 20, specifically at T790M, are commonly seen in first-generation EGFR inhibitors such as gefitinib and erlotinib, which are used to treat non-small-cell lung cancer. Threonine mutations at region 790 of EGFR create a large chain of methionine residues, and the resulting steric hindrance prevents EGFR RTKs from binding [5,6,7].

The L858R mutation detected in exon 20 is also one of the factors that can lead to the discontinuation of treatment. Here, the replacement of the leucine 858 residue with arginine results in ligand-autonomic signaling. This allows the ligand to remain constantly stimulated without needing a separate stimulus [8,9].

Benzimidazole is one of the important heterocyclic ring systems that can be used for the development of new anticancer agents because its structure shows structural similarities to the basic components of DNA and RNA. Its ease of synthesis and its significant activities as a topoisomerase inhibitor, androgen receptor antagonist, microtubule inhibitor, and protein kinase inhibitor have made the benzimidazole ring prominent in new drug development studies. It is also known to be present in the structure of some newly approved drugs that are in the clinical development phase [10,11]. A review of the literature reveals that benzimidazole has been involved in the development of various EGFR inhibitor compounds [10,11,12,13,14].

The triazole ring is a five-membered aromatic heterocycle of significant importance in medicinal chemistry. Its electronic properties and ability to participate in hydrogen-bonding interactions contribute to its potential biological activity and molecular recognition. In addition, triazole can act as a bioisostere of amide functionalities. Compounds combining benzimidazole and triazole moieties and exhibiting EGFR inhibitory activity have been reported previously [15,16,17].

In our design strategy, a series of compounds was designed based on selected structural and pharmacophoric motifs observed in anticancer agents such as letrozole, afatinib, dacomitinib, sorafenib, and tivozanib, which have previously been reported to exhibit anticancer activity (Figure 1). These drugs are included in the National Cancer Institute’s list of targeted therapy drugs approved for specific types of cancer [18]. These reference compounds were selected to highlight relevant structural features, including triazole, benzimidazole/quinazoline-related heteroaromatic, and amide/urea moieties, rather than to imply identical mechanisms of action. In this study, ten novel compounds containing benzimidazole and triazole scaffolds were synthesized as potential EGFR inhibitors. Their biological activities were subsequently evaluated to determine their inhibitory potential against wild-type EGFR, EGFR-L858R, and the drug-resistant EGFR-L858R/T790M mutant.

## 2. Results and Discussion

### 2.1. Chemistry

The synthetic route and the substituents of the synthesized final compounds are presented in Scheme 1. The synthesis of compounds **1a**–**4a** was performed by applying synthetic approaches, previously developed and reported in the literature for the preparation of the present benzimidazole-1,2,4-triazole hybrid scaffold [19,20,21,22]. Thus, the synthetic strategy employed in this study represents an extension of our previously established methodologies to new hybrid structures rather than an entirely unprecedented synthetic approach.

Methyl 2-(4-hydroxyphenyl)-1*H*-benzo[*d*]imidazole-6-carboxylate (**1a**) was synthesized by the reaction of methyl 3,4-diaminobenzoate with 4-hydroxybenzaldehyde. Subsequently, 2-(4-hydroxyphenyl)-1*H*-benzo[*d*]imidazole-6-carbohydrazide (**2a**) was obtained by reacting compound **1a** with an excess of hydrazine hydrate. The resulting carbohydrazide derivative was then reacted with ethyl isothiocyanate to afford *N*-ethyl-2-(2-(4-hydroxyphenyl)-1*H*-benzo[*d*]imidazole-6-carbonyl)hydrazine-1-carbothioamide (**3a**). Compound **3a** was reacted with sodium hydroxide under reflux to give synthesis of 4-(6-(4-ethyl-5-mercapto-4*H*-1,2,4-triazol-3-yl)-1*H*-benzo[*d*]imidazol-2-yl)phenol (**4a**). Compound **4a** and the appropriate acetamide derivatives were then reacted to obtain target compounds **5a**–**5j**.

This general synthetic concept has been applied before [23]; however, we have extended it to a new series of benzimidazole-1,2,4-triazole-acetamide hybrids. The present work focuses on the design and synthesis of a new series of benzimidazole-1,2,4-triazole hybrid derivatives bearing acetamide moieties and on the investigation of their biological properties. In this context, the established synthetic approaches provide a useful basis for the construction of these new hybrid structures.

The employed synthetic strategy provides a practical route to the target compounds by extending established methodologies to the present hybrid scaffold. The microwave-assisted synthesis of the benzimidazole intermediate enabled preparation of the **5a**–**5j** series with structural diversity in the acetamide substituents.

The structures of the final compounds **5a**–**5j** were elucidated by the ^1^H-NMR, ^13^C-NMR, and HRMS spectroscopic methods. In the ^1^H-NMR spectra, all aromatic protons were observed in the range of 6.86–8.36 ppm, while the ^13^C-NMR spectra showed the expected signals for the aromatic carbon atoms.

The HRMS data were in good agreement with the calculated M+H values of the synthesized triazole–benzimidazole derivatives. Purity analysis showed that the compounds had purities ranging from 94.130% to 100%. The analytical spectra of all synthesized triazole–benzimidazole derivatives are provided in the Supplementary Materials (Figures S1–S42).

To support the structural characterization of the compound and to achieve precise assignments of the carbon signals, Heteronuclear Single Quantum Coherence (HSQC) and Heteronuclear Multiple Bond Correlation (HMBC) analyses were performed as part of the two-dimensional NMR studies. The HSQC spectrum was used to determine the direct ^1^H-^13^C correlations of proton-bearing carbon atoms. Based on the obtained HSQC data, the signal corresponding to the methyl carbon of the ethyl group attached to the triazole ring was observed at *δ*_C_ 15.6 ppm, whereas the signal for the methylene carbon of the same ethyl group was found to overlap with the DMSO-*d*_6_ solvent signal. It was determined that the methyl carbons of the dimethylamino group resonated at *δ*_C_ 35.8 and 37.6 ppm, while the methylene carbon adjacent to the carbonyl group resonated at *δ*_C_ 37.05 ppm. Examination of the aromatic region revealed signals at *δ*_C_ 116.5 ppm for the protonated carbons located near the hydroxy group on the 1,4-disubstituted benzene ring, and signals at *δ*_C_ 128.5 ppm for the other protonated carbons. The proton-bearing carbons on the other benzene ring were found to resonate at *δ*_C_ 115.5 and 122.5 ppm.

The HMBC spectrum, which displays long-range ^1^H-^13^C correlations, was utilized to identify non-protonated quaternary carbons that could not be directly assigned via HSQC analysis. Based on the HMBC data, the signal for the carbon of the triazole ring attached to the benzimidazole group was identified at *δ*_C_ 156.6 ppm, while the signal for the other carbon in the triazole ring was identified at *δ*_C_ 150.0 ppm. The carbonyl carbon was assigned a chemical shift of *δ*_C_ 167.3 ppm based on long-range correlations observed with the -CH_2_- protons adjacent to the carbonyl group. Within the benzene ring, the quaternary carbon attached to the hydroxy group was identified at *δ*_C_ 120.3 ppm, while the carbon attached to the benzimidazole group was found at *δ*_C_ 128.9 ppm. The carbon at the 2-position of the benzimidazole ring was determined to resonate at *δ*_C_ 154.4 ppm, whereas the carbon of the benzimidazole ring attached to the triazole group was assigned a value of *δ*_C_ 120.7 ppm. Carbon signals associated with the 4- and 5-positions of the benzimidazole ring were observed around *δ*_C_ 140.4 ppm. Overall, the correlations obtained from HSQC and HMBC analyses, when evaluated alongside the ^1^H and ^13^C NMR data, enabled the assignment of the relevant carbon signals in a manner consistent with the structure and supported the proposed molecular structure (Figures S41 and S42).

### 2.2. Cytotoxicity Assay

Of the ten synthesized compounds, 4 (**5a**, **5c**, **5d** and **5i**) had IC_50_ values ≤ 100 μM and were considered effective against the A549 cell line, while 2 (**5a** and **5i**) were effective against NIH/3T3. Of these, **5a** and **5i** were more active against the A549 cell line, while **5i** was more active against the NIH/3T3 cell line. Compound **5a** exhibited the highest cytotoxic activity against A549 cells, with an IC_50_ value of 6.01 ± 0.76 µM, surpassing that of the reference drug Doxorubicin (9.14 ± 0.18 µM). However, its IC_50_ value against NIH/3T3 cells (12.43 ± 0.87 µM) indicated limited selectivity toward cancer cells. Nevertheless, owing to its pronounced activity against A549 cells, compound **5a** was selected for further molecular docking and molecular dynamics studies to investigate its potential molecular interactions and binding stability. It can be concluded that the synthesized compounds exhibit better inhibitory activity on EGFR (WT). When the amide derivatives attached to the thiol group of the triazole ring were examined, compounds **5a**, **5c**, and **5i**, which are methyl-substituted derivatives, were identified as the active derivatives in the series. The relevant results are presented in Table 1. Dose–response curves for all compounds in A549 cells were determined by the MTT test, and detailed graphs are shared in the Supplementary Materials file (Figures S43–S52).

### 2.3. In Vitro EGFR Inhibition Assay

Compound **5a**, which exhibited relatively high activity and low toxicity in the MTT assay, showed the highest to lowest EGFR inhibitory activity against EGFR (WT), EGFR-L858R, and EGFR-L858R/T790M, respectively. Furthermore, compound **5a** exhibited 88.039% inhibitory activity on EGFR at a concentration of 1000 µM and 86.378% at a concentration of 100 µM. Compound **5a** in the series exhibited good activity against EGFR at concentrations of 1000 µM and 100 µM, but when IC_50_ values (0.068 ± 0.008, 0.269 ± 0.011, 5.267 ± 0.109 µM) were compared, it was assessed to have an approximately 4-fold lower inhibitory activity on the single-mutated (EGFR-L858R) form and an approximately 77-fold lower inhibitory activity on the double-mutated (EGFR-L858R/T790M) form. The relevant results are presented in Table 2. Kinase experiments were conducted as four independent experiments at eight different concentrations; detailed graphs are presented in the Supplementary Materials file (Figures S53–S55).

### 2.4. ADME Predictions

To retrospectively characterize the drug-likeness and predicted ADME properties of the synthesized compounds, compounds **5a**–**5j** generally exhibited favorable drug-likeness profiles. All obtained compounds conformed to Lipinski’s rules, while many compounds also met the Ghose, Veber, and Muegge rules. Among the compounds in the series, compound **5f** met all five drug-likeness rules. In terms of water solubility, compounds **5a** and **5c** were classified as soluble, while the remaining compounds were estimated to be moderately soluble. Gastrointestinal absorption was estimated to be high for compounds **5a**, **5f**, and **5h**, while other derivatives showed low absorption. Notably, all compounds showed the same estimated oral bioavailability score (0.55). These data indicate that the synthesized compounds possess acceptable levels of drug-like properties, and compound **5f** has the best ADME profile in the series (Table 3 and Table 4).

When the BOILED-Egg analysis is examined, it is observed that all compounds except **5g** and **5f** in the **5a**–**5j** series are located outside the white and yellow regions. This indicates that the compounds may have high TPSA values, meaning they have higher polarity, which may lead to lower gastrointestinal absorption and lower probability of crossing the blood–brain barrier. Compound **5c**, in particular, stands out more distinctly due to its highest TPSA value. Furthermore, the blue coloration of all compounds suggests that they may be P-glycoprotein (P-gp) substrates and may be subject to excretion mechanisms. Therefore, it is thought that the synthesized compounds may have low permeability to the central nervous system and high oral bioavailability due to their high polarity (Figure 2).

When the predicted physicochemical domains for oral bioavailability obtained with the help of the SwissADME program were examined, it was observed that the physicochemical properties of the compounds in the **5a**–**5j** series were generally within the recommended ranges for oral drug-like molecules. All compounds exhibited a stable profile in terms of lipophilicity, molecular size, polarity, and molecular flexibility. However, some compounds showed limited deviations in the parameters of unsaturation (INSATU) and solubility (INSOLU). The results indicate that the synthesized compounds may possess suitable ADME properties and are promising in terms of oral bioavailability (Figure 3).

### 2.5. Molecular Docking Studies

Docking studies were performed using crystals with PDB IDs 4HJO, 2ITZ, and 4I22, containing the mutations EGFR, EGFR-L858R, and EGFR-L858R/T790M, respectively.

The interaction domain of EGFR (PDB ID: 4HJO) and its cocrystal ligand Erlotinib (PDB ID: AQ4) with the A-chain had been previously identified; LEU694, VAL702, ALA719, LYS721, LEU764, THR766, GLN767, LEU768, MET769, PRO770, GLY772, LEU820, THR830, ASP831 and HOH1104 were highlighted as important for the interaction (https://www.ebi.ac.uk/pdbe/entry/pdb/4hjo/bound/AQ4#1001A, accessed on 1 July 2026). Figure 4 shows 2D and 3D images of the interactions of compound **5a** with EGFR (PDB: 4HJO). Examination of the binding positions of compound **5a** clearly reveals that it exhibits hydrogen bond interactions. The study shows that the benzimidazole and amide nitrogen atoms of compound **5a** form hydrogen bonds with THR766 and MET769.

The interaction domain of EGFR-L858R (PDB ID: 2ITZ) and its cocrystal ligand Gefitinib (PDB ID: IRE) with the A-chain had been previously identified; LEU718, ALA743, LYS745, MET766, THR790, GLN791, LEU792, MET793, PRO794, GLY796, ASP800, LEU844, ASP855, HOH3024, and HOH3033 were highlighted as important for the interaction (https://www.ebi.ac.uk/pdbe/entry/pdb/2itz?activeTab=ligands&id=IRE, accessed on 1 July 2026). Figure 4 shows 2D and 3D images of the interactions of compound **5a** with EGFR-L858R (PDB: 2ITZ). Examination of the binding positions of compound **5a** clearly reveals that it exhibits hydrogen bond interactions. The study shows that the benzimidazole and amide nitrogen atoms of compound **5a** form hydrogen bonds with GLU762, PHE795 and CYS797.

The interaction domain EGFR-L858R/T790M (PDB ID: 4I22) and its cocrystal ligand Gefitinib (PDB ID: IRE) with the A-chain had been previously identified; LEU718, VAL726, ALA743, LYS745, LEU788, MET790, GLN791, LEU792, MET793, GLY796, ASP800, LEU844, ASP855, HOH9101, HOH9102, HOH9150, HOH9156, HOH9183 and HOH9224 were highlighted as important for the interaction (https://www.ebi.ac.uk/pdbe/entry/pdb/4i22?activeTab=ligands, accessed on 1 July 2026). Figure 4 shows 2D and 3D images of the interactions of compound **5a** with EGFR-L858R/T790M (PDB: 4I22). Examination of the binding positions of compound **5a** clearly reveals that it exhibits hydrogen bond interactions. The study shows that the benzimidazole and amide nitrogen atoms of compound **5a** form hydrogen bonds with LYS716 and ASP855.

When the interactions of **5a** and 4HJO were examined, it was determined that the benzimidazole and amide nitrogens of **5a** hydrogen bonded with the THR766 and MET769 residues of 4HJO, respectively. When the interactions of **5a** and 2ITZ were examined, it was determined that the triazole and amide nitrogens of **5a** hydrogen bonded with the PHE795 and CYS797 residues of 2ITZ, respectively. In addition, a hydrogen bond was detected between the phenol hydroxyl of **5a** and GLU762. When the interactions of **5a** and 4I22 were examined, it was determined that the carbonyl of the amide of **5a** hydrogen bonded with the LYS716 residue of 4I22, respectively. In addition, a hydrogen bond was detected between the phenol hydroxyl of **5a** and ASP855 (Figure 4). When in vitro and in silico findings are evaluated together, two-dimensional interaction diagrams of molecular docking studies performed with all compounds for wild-type EGFR (PDB ID: 4HJO), where higher inhibitory activity was observed, are presented in Figures S56–S64 in the Supplementary Materials file.

### 2.6. Molecular Dynamic Studies

Molecular docking studies were conducted to predict the possible binding patterns of compound **5a** to target proteins, and molecular dynamics simulations were performed to validate these results. In molecular dynamics (MD) simulations, the crystal structures EGFR (PDB ID: 4HJO), EGFR-L858R (PDB ID: 2ITZ), and EGFR-L858R/T790 M (PDB ID: 4I22) were also used.

For molecular dynamics simulations, RMSD (root mean square deviation) is a parameter indicating structural stability over time, and a value close to 2 Å is desirable. The RMSD value for compound **5a** with the 4HJO complex was found to be 2.25 Å (Figure 5), for the 2ITZ complex 2.8 Å (Figure 6), and for the 4I22 complex 2.4 Å (Figure 7). Analysis of the dynamic results revealed that compound **5a** exhibited better inhibitory properties on the unmutated EGFR enzyme (PDB ID: 4HJO). In vitro results also showed that compound **5a** was more effective on the unmutated EGFR enzyme. It was also determined that 4HJO forms hydrogen bonds similarly to the MET769 residue, which is important due to its hydrogen-bonding properties.

For compound **5a**, it can be said that GLU762, PHE795, and CYS797 hydrogen bond with single-mutated EGFR-L858R, and LYS716 and ASP855 hydrogen bond with double-mutated EGFR-L858R/T790M; however, among the interactions stated to be important for cocrystal gefitinib, it is only compatible with ASP855, while the others are not significant interactions. This explains the reason for the higher inhibitory effect on unmutated EGFR.

Figure 5E shows the interactions between compound **5a** and the EGFR enzyme. The presence of a hydrogen bond interaction between compound **5a** and the amino acid MET769, which is critically important in the active site of 4HJO, and the observation of a continuous interaction supports the more favorable results obtained in in vitro studies.

These synthesized compounds are expected to show higher efficacy and minimal toxicity in cancer cells carrying EGFR mutation-free cells, rather than in cancer cells carrying EGFR mutations.

To more comprehensively evaluate the activity profiles of the compounds and to reveal the molecular basis of the differences between the active and inactive derivatives, molecular dynamics (MD) simulations were also performed for the inactive derivatives **5i** and **5j**. The MD analysis results for these compounds are presented in Figures S65 and S66, respectively. The findings showed that despite being biologically inactive, both compounds exhibited a certain conformational stability and maintained their positions in the enzyme’s active site throughout the 100 ns simulation period. However, one of the most striking differences between the active and inactive derivatives was observed in the persistence of interactions with the amino acid MET769, which is critical for EGFR inhibition. While the active derivative **5a** maintained its interaction with MET769 for approximately 60% of the simulation period, this interaction rate remained below 10% for the inactive derivatives **5i** and **5j**. These results suggest that the stable presence of compounds in the EGFR active site may not be sufficient for inhibitory activity alone; rather, the persistence of interactions with critical amino acids, such as MET769, and the high interaction rate may play a significant role in explaining the observed activity.

## 3. Methods and Materials

### 3.1. General Information

All chemicals and reagents used in this study were purchased from Merck (Darmstadt, Germany) and Sigma-Aldrich (St. Louis, MO, USA) and used without further purification. The melting points of the synthesized compounds were determined using an MP90 digital melting point apparatus (Mettler Toledo, Columbus, OH, USA). LC–MS/MS analyses were performed using a Shimadzu 8040 LC–MS/MS system (Shimadzu, Kyoto, Japan). The purity of the synthesized compounds was assessed using an IT-TOF mass spectrometer equipped with a photodiode array (PDA) detector. ^1^H-NMR and ^13^C-NMR spectra were recorded in DMSO-*d*_6_ using Bruker FT-NMR spectrometers operating at 300 and 75 MHz, respectively (Bruker, Billerica, MA, USA). In the NMR spectra, chemical shifts are reported in ppm, and the coupling constants (*J*) are given in Hz. The splitting patterns were designated as singlet (s), doublet (d), triplet (t), and multiplet (m).

### 3.2. Chemistry

*Synthesis of Methyl 2-(4-hydroxyphenyl)-1H-benzo[d]imidazole-6-carboxylate* (**1a**)

Methyl 3,4-diaminobenzoate (5.00 g, 0.02 mol), 4-hydroxybenzaldehyde (2.44 g, 0.02 mol), sodium disulfite (2.21 g, 0.024 mol), and dimethylformamide (30 mL) were reacted using the microwave-assisted synthesis method. The compound was synthesized following the procedure previously reported in the literature [19,20,21,22].

*Synthesis of 2-(4-hydroxyphenyl)-1H-benzo[d]imidazole-6-carbohydrazide* (**2a**)

Methyl 2-(4-hydroxyphenyl)-1*H*-benzo[d]imidazole-6-carboxylate (4.02 gr, 0.015), ethanol (25 mL) and hydrazine hydrate (1.80 mL, 0.045 mol) were added. The reaction mixture refluxed at 79 °C for 10 h. At the end of the reaction, the product was poured into ice water to precipitate it, filtered, washed with plenty of water, and crystallized from ethanol. The compound was synthesized following the procedure previously reported in the literature [19,21].

*Synthesis of N-ethyl-2-(2-(4-hydroxyphenyl)-1H-benzo[d]imidazole-6-carbonyl)hydrazine-1-carbothioamide* (**3a**)

2-(4-Hydroxyphenyl)-1*H*-benzo[d]imidazole-6-carbohydrazide (**2a**) (3.45 gr, 0.013 mol) and ethyl isothiocyanate (1.14 mL, 0.013 mol) were dissolved in ethanol (30 mL) and stirred under reflux at 79 °C for 4 h. The precipitated product was filtered and dried. The compound was synthesized following the procedure previously reported in the literature [23].

*Synthesis of 4-Ethyl-5-(2-(4-hydroxyphenyl)-1H-benzo[d]imidazol-6-yl)-2,4-dihydro-3H-1,2,4-triazole-3-thione* (**4a**)

The dried product (**3a**) (3.65 gr, 0.010 mol) was added to the sodium hydroxide (0.48 gr, 0.012 mol) solution in ethanol (30 mL) and stirred under reflux for 4 h at 79 °C. At the end of the reaction, HCl was added to the product until pH = 4; the product was precipitated by pouring it into ice water and was crystallized from ethanol by washing with plenty of water. The compound was synthesized following the procedure previously reported in the literature [23].

*General procedures of target compounds* (**5a**–**5j**)

4-Ethyl-5-(2-(4-hydroxyphenyl)-1*H*-benzo[*d*]imidazol-6-yl)-2,4-dihydro-3*H*-1,2,4-triazole-3-thione (**4a**) (0.33 g, 0.001 mol) and appropriate acetamide derivatives (0.001 mol) [2-Chloro-*N*-methylacetamide (Cas No: 96-30-0); *N*-Isopropyl-2-chloroacetamide (Cas No: 2895-21-8); 2-Chloro-*N*-(hydroxymethyl)acetamide (Cas No: 2832-19-1); 2-Chloro-*N*-methoxy-*N*-methylacetamide (Cas No: 67442-07-3); 2-Chloro-*N*-cyclopropylacetamide (Cas No: 19047-31-5); 2-Chloro-*N*,*N*-dimethylacetamide (Cas No: 2675-89-0); 2-Chloro-*N*,*N*-diethylacetamide (Cas No: 2315-36-8); 2-Chloro-1-morpholinoethan-1-one (Cas No: 1440-61-5); 2-Chloro-*N*-phenylacetamide (Cas No: 587-65-5); 2-Chloro-1-(4-chlorophenyl)ethan-1-one (Cas No: 937-20-2)] were stirred for 4 h in acetone (20 mL) in the presence of potassium carbonate (0.138 gr, 0.001 mol) at 25 °C. After completion of the reaction, acetone was removed under reduced pressure and the residue was washed with water, dried, and recrystallized from EtOH.

*2-((4-Ethyl-5-(2-(4-hydroxyphenyl)-1H-benzo[d]imidazol-6-yl)-4H-1,2,4-triazol-3-yl)thio)-N-methylacetamide* (**5a**)

C_20_H_20_N_6_O_2_S, yield: 75%, melting point: 253.4–256.4 °C. ^1^H-NMR (300 MHz, DMSO-*d*_6_): δ = 1.24 (3H, t, *J* = 6.35 Hz, -CH_3_), 2.63 (3H, s, -CH_3_), 3.96 (2H, s, -CH_2_), 4.05 (2H, d, *J* = 6.40 Hz, -CH_2_), 6.93 (2H, d, *J* = 7.76 Hz, Ar-H), 7.41 (H, d, *J* = 8.06 Hz, Ar-H), 7.69 (H, d, *J* = 8.06 Hz, Ar-H), 7.78 (H, s, -NH), 8.05 (2H, d, *J* = 7.74 Hz, Ar-H), 8.24 (H, s, Ar-H). ^13^C-NMR (75 MHz, DMSO-*d*_6_): δ = 15.6, 26.4, 33.9, 36.9, 115.4, 116.3, 117.1, 120.8, 122.2, 122.6, 128.0, 128.9, 140.9, 149.9, 154.1, 156.2, 160.4, 167.7. HRMS (*m*/*z*): [M + H]^+^ calculated: 409.1441; found: 409.1416.

*2-((4-Ethyl-5-(2-(4-hydroxyphenyl)-1H-benzo[d]imidazol-6-yl)-4H-1,2,4-triazol-3-yl)thio)-N-isopropylacetamide* (**5b**)

C_22_H_24_N_6_O_2_S, yield: 78%, melting point: 189.5–191.9 °C. ^1^H-NMR (300 MHz, DMSO-*d*_6_): δ = 1.05 (6H, d, *J* = 5.16 Hz, 2.-CH_3_), 1.25 (3H, s, -CH_3_), 3.82 (H, d, *J* = 5.52 Hz, -CH), 3.91 (2H, s, -CH_2_),4.07 (2H, d, *J* = 5.64 Hz, -CH_2_), 6.94 (2H, d, *J* = 7.28 Hz, Ar-H), 7.41 (H, s, NH), 7.65–7.82 (2H, m, Ar-H), 8.05 (2H, d, *J* = 7.28 Hz, Ar-H), 8.16 (H, d, *J* = 5.12 Hz, Ar-H), 10.05 (H, s, OH), 12.97 (H, s, NH). ^13^C-NMR (75 MHz, DMSO-*d*_6_): δ = 15.7, 21.9, 22.7, 37.6, 42.3, 112.5, 116.2, 117.1, 118.0, 118.1, 120.9, 121.0, 122.7, 128.9, 149.8, 153.9, 156.1, 159.9, 166.2. HRMS (*m*/*z*): [M + H]^+^ calculated: 437.1754; found: 437.1753.

*2-((4-Ethyl-5-(2-(4-hydroxyphenyl)-1H-benzo[d]imidazol-6-yl)-4H-1,2,4-triazol-3-yl)thio)-N-(hydroxymethyl)acetamide* (**5c**)

C_20_H_20_N_6_O_3_S, yield: 79%, melting point: 162.9–165.7 °C. ^1^H-NMR (300 MHz, DMSO-*d*_6_): δ = 1.25 (3H, s, -CH_3_), 3.69–3.99 (4H, m, 2.-CH_2_),, 4.06 (2H, d, *J* = 4.35 Hz, -CH_2_), 4.55 (H, s, -OH), 6.93 (2H, d, *J* = 7.49 Hz, Ar-H), 7.28 (H, s, NH), 7.41 (H, d, *J* = 8.04 Hz, Ar-H), 7.69 (H, d, *J* = 7.96 Hz, Ar-H), 7.74–7.77 (H, m, Ar-H), 8.04 (2H, d, *J* = 7.40 Hz, Ar-H). ^13^C-NMR (75 MHz, DMSO-*d*_6_): δ = 15.6, 37.1, 37.3, 63.2, 115.7, 116.3, 120.8, 122.6, 128.9, 132.8, 149.9, 150.0, 154.1, 156.2, 156.2, 160.5, 167.5, 169.3. HRMS (*m*/*z*): [M + H]^+^ calculated: 425.1390; found: 425.1371.

*2-((4-Ethyl-5-(2-(4-hydroxyphenyl)-1H-benzo[d]imidazol-6-yl)-4H-1,2,4-triazol-3-yl)thio)-N-methoxy-N-methylacetamide* (**5d**)

C_21_H_22_N_6_O_3_S, yield: 73%, melting point: 186.4–188.9 °C. ^1^H-NMR (300 MHz, DMSO-*d*_6_): δ = 1.26 (3H, s, -CH_3_), 3.17 (3H, s, -CH_3_), 3.78 (3H, s, -CH_3_), 4.07 (2H, d, *J* = 6.21 Hz, -CH_2_), 4.38 (2H, s, -CH_2_), 6.94 (2H, d, *J* = 7.85 Hz, Ar-H), 7.41 (H, d, *J* = 7.60 Hz, Ar-H), 7.70 (H, d, *J* = 7.28 Hz, Ar-H), 7.77 (H, s, Ar-H), 8.05 (2H, d, *J* = 7.44 Hz, Ar-H). ^13^C-NMR (75 MHz, DMSO-*d*_6_): δ = 15.5, 32.6, 32.6, 35.6, 61.7, 113.2, 116.3, 120.8, 121.0, 122.6, 128.9, 130.6, 144.8, 149.8, 153. 9, 154.0, 156.2, 160.2, 168.2. HRMS (*m*/*z*): [M + H]^+^ calculated: 439.1547; found: 439.1549.

*N-Cyclopropyl-2-((4-ethyl-5-(2-(4-hydroxyphenyl)-1H-benzo[d]imidazol-6-yl)-4H-1,2,4-triazol-3-yl)thio)acetamide* (**5e**)

C_22_H_22_N_6_O_2_S, yield: 76%, melting point: 167.5–170.3 °C. ^1^H-NMR (300 MHz, DMSO-*d*_6_): δ = 0.40 (2H, s, -CH_2_), 0.63 (2H, d, *J* = 5.98 Hz, -CH_2_), 1.25 (3H, t, *J* = 6.98 Hz, -CH_3_), 2.63 (H, s, -CH), 3.90 (2H, s, -CH_2_), 4.06 (2H, d, *J* = 6.20 Hz, -CH_2_), 6.92 (2H, d, *J* = 7.85 Hz, Ar-H), 7.40 (H, d, *J* = 8.19 Hz, Ar-H), 7.69 (H, d, *J* = 7.92 Hz, Ar-H), 7.76 (H, s, Ar-H), 8.04 (2H, d, *J* = 7.39 Hz, Ar-H), 8.36 (H, s, NH). ^13^C-NMR (75 MHz, DMSO-*d*_6_): δ = 6.1, 15.6, 23.1, 37.0, 37.2, 116.4, 120.0, 120.7, 120.8, 122.6, 128.9, 141.1, 149.8, 152.8, 154.2, 156.2, 158.8, 160.6, 168.4. HRMS (*m*/*z*): [M + H]^+^ calculated: 435.1598; found: 435.1604.

*2-((4-Ethyl-5-(2-(4-hydroxyphenyl)-1H-benzo[d]imidazol-6-yl)-4H-1,2,4-triazol-3-yl)thio)-N,N-dimethylacetamide* (**5f**)

C_21_H_22_N_6_O_2_S, yield: 72%, melting point: 183.1–186.0 °C. ^1^H-NMR (300 MHz, DMSO-*d*_6_): δ = 1.26 (3H, s, -CH_3_), 2.88 (3H, s, -CH_3_), 3.06 (3H, s, -CH_3_), 4.07 (2H, d, *J* = 6.14 Hz, -CH_2_), 4.35 (2H, s, -CH_2_), 6.91 (2H, d, *J* = 7.56 Hz, Ar-H), 7.40 (H, d, *J* = 8.20 Hz, Ar-H), 7.68 (H, d, *J* = 8.12 Hz, Ar-H), 7.75 (H, s, Ar-H), 8.03 (2H, d, *J* = 7.56 Hz, Ar-H). ^13^C-NMR (75 MHz, DMSO-*d*_6_): δ = 15.6, 35.8, 37.5, 37.6, 115.5, 116.5, 120.3, 120.7, 122.5, 128.5, 128.9, 140.4, 150.0, 154.4, 156.2, 161.2, 167.3. HRMS (*m*/*z*): [M + H]^+^ calculated: 423.1598; found: 423.1566.

*N,N-Diethyl-2-((4-ethyl-5-(2-(4-hydroxyphenyl)-1H-benzo[d]imidazol-6-yl)-4H-1,2,4-triazol-3-yl)thio)acetamide* (**5g**)

C_23_H_26_N_6_O_2_S, yield: 77%, melting point: 187.5–185.3 °C. ^1^H-NMR (300 MHz, DMSO-*d*_6_): δ = 1.04 (3H, s, -CH_3_), 1.18 (3H, s, -CH_3_), 1.26 (3H, s, -CH_3_), 3.31 (2H, d, *J* = 6.24 Hz, -CH_2_), 3.40 (2H, d, *J* = 6.11 Hz, -CH_2_), 4.07 (2H, d, *J* = 5.78 Hz, -CH_2_), 4.34 (2H, s, -CH_2_), 6.93 (2H, d, *J* = 7.48 Hz, Ar-H), 7.41 (H, d, *J* = 7.88 Hz, Ar-H), 7.69 (H, d, *J* = 7.72 Hz, Ar-H), 7.76 (H, s, Ar-H), 8.04 (2H, d, *J* = 7.36 Hz, Ar-H). ^13^C-NMR (75 MHz, DMSO-*d*_6_): δ = 13.4, 14.6, 15.6, 37.4, 42.4, 116.3, 120.9, 121.0, 122.6, 128.6, 128.9, 137.3, 150.0, 154.0, 155.4, 155.6, 156.1, 160.2, 166.2. HRMS (*m*/*z*): [M + H]^+^ calculated: 451.1911; found: 451.1867.

*2-((4-Ethyl-5-(2-(4-hydroxyphenyl)-1H-benzo[d]imidazol-6-yl)-4H-1,2,4-triazol-3-yl)thio)-1-morpholinoethan-1-one* (**5h**)

C_23_H_24_N_6_O_3_S, yield: 74%, melting point: 183.3–186.3 °C. ^1^H-NMR (300 MHz, DMSO-*d*_6_): δ = 1.26 (3H, s, -CH_3_), 3.47–3.62 (8H, m, morpholine(4.-CH_2_)), 4.08 (2H, d, *J* = 6.16 Hz, -CH_2_), 4.34 (2H, s, -CH_2_), 6.89 (2H, d, *J* = 7.59 Hz, Ar-H), 7.38 (H, d, *J* = 8.21 Hz, Ar-H), 7.67 (H, d, *J* = 8.09 Hz, Ar-H), 7.75 (H, s, Ar-H), 8.02 (2H, d, *J* = 7.60 Hz, Ar-H). ^13^C-NMR (75 MHz, DMSO-*d*_6_): δ = 15.7, 37.1, 42.4, 46.4, 66.4, 115.2, 116.3, 116.5, 120.2, 120.6, 122.4, 128.9, 137.4, 149.7, 153.9, 154.5, 156.2, 161.5, 166.1. HRMS (*m*/*z*): [M + H]^+^ calculated: 465.1661; found: 465.1703.

*2-((4-Ethyl-5-(2-(4-hydroxyphenyl)-1H-benzo[d]imidazol-6-yl)-4H-1,2,4-triazol-3-yl)thio)-N-phenylacetamide* (**5i**)

C_25_H_22_N_6_O_2_S, yield: 71%, melting point: 213.2–215.3 °C. ^1^H-NMR (300 MHz, DMSO-*d*_6_): δ = 1.15–1.26 (3H, m, -CH_3_), 4.07 (2H, d, *J* = 5.28 Hz, -CH_2_), 4.21 (2H, s, -CH_2_), 6.86 (2H, d, *J* = 7.08 Hz, Ar-H), 7.06–7.10 (H, m, Ar-H), 7.31–7.36 (3H, m, Ar-H), 7.59–7.65 (3H, m, Ar-H), 7.73 (H, s, Ar-H), 8.01 (2H, d, *J* = 7.20 Hz, Ar-H), 10.45 (H, s, -OH). ^13^C-NMR (75 MHz, DMSO-*d*_6_): δ = 15.6, 32.1, 38.1, 114.4, 115.1, 116.78, 119.4, 119.6, 120.2, 122.2, 124.0, 128.8, 129.3, 133.8, 139.3, 149.7, 155.0, 156.4, 159.8, 162.8, 166.2. HRMS (*m*/*z*): [M + H]^+^ calculated: 471.1598; found: 471.1548.

*N-(4-chlorophenyl)-2-((4-ethyl-5-(2-(4-hydroxyphenyl)-1H-benzo[d]imidazol-6-yl)-4H-1,2,4-triazol-3-yl)thio)acetamide* (**5j**)

C_25_H_21_N_6_O_2_SCl, yield: 80%, melting point: 145.0–148.3 °C. ^1^H-NMR (300 MHz, DMSO-*d*_6_): δ = 1.26 (3H, s, -CH_3_), 4.08 (2H, d, *J* = 5.90 Hz, -CH_2_), 4.22 (2H, s, -CH_2_), 6.95 (2H, d, *J* = 7.46 Hz, Ar-H), 7.39 (3H, d, *J* = 8.82 Hz, Ar-H), 7.64 (3H, d, *J* = 7.71 Hz, Ar-H), 7.70 (H, s, Ar-H), 7.77 (H, s, -NH), 8.05 (2H, d, *J* = 7.40 Hz, Ar-H), 10.07 (H, s, -OH), 10.57 (H, s, -NH). ^13^C-NMR (75 MHz, DMSO-*d*_6_): δ = 15.6, 38.0, 44.0, 115.7, 116.2, 120.8, 121.1, 121.1, 121.4, 127.6, 127.9, 128.9, 129.2, 137.9, 138.2, 149.8, 153.9, 156.3, 160.0, 165.3, 166.4. HRMS (*m*/*z*): [M + H]^+^ calculated: 505.1211; found: 505.1208. The characteristic M/M + 2 isotopic pattern attributable to the presence of one chlorine atom was observed at *m*/*z* 505.1211 and 507.1132, respectively.

### 3.3. Cytotoxicity Assay

To prepare A549 (human lung cancer cell line) and NIH/3T3 (mouse embryonic fibroblast cell line) cells for use in the experiments, routine passage was performed every 2–3 days. The cell culture flask removed from the incubator was gently shaken to allow dead cells to pass into the culture medium solution, and then the culture medium inside the flask was removed with a sterile pipette and discarded. To wash the cells, 5 mL of phosphate buffer was added to the culture flask, and the washing solution was removed from the medium. Trypsin-EDTA solution (1X) (3–5 mL for 75 cm^2^ culture flasks, 1–3 mL for 25 cm^2^) was added to the culture flask, gently shaken, and incubated for approximately 5 min (5% CO_2_, 95% humidity, and 37 °C). The cells were suspended in culture flasks taken from the incubator by adding 20–25 mL of culture medium, then divided into 1:2 or 1:3 ratios and transferred to new culture flasks. The culture flasks were placed in the incubator and incubated (5% CO_2_, 95% humidity, and 37 °C). The culture flasks were removed from the incubator, and culture medium was added, followed by the addition of trypsin-EDTA solution at a volume of at least twice that of the culture medium. The resulting cell suspension was transferred into centrifuge tubes using a pipette. After homogenization of the cell suspension, a 10 µL aliquot was collected and counted using an automated cell counter. The cell suspension was then transferred into cuvettes and seeded into a cell culture plate at 200 µL/well, corresponding to a concentration of 1104 cells/100 µL. The cells were incubated for 24 h at 37 °C, 5% CO_2_, and 95% humidity. Test agent solutions were prepared in DMSO at concentrations ranging from 0.00316 to 10 mM and added to the plates along with the negative and positive controls. The plates were subsequently incubated for 24 h under the same conditions (37 °C, 5% CO_2_, and 95% humidity). Following incubation, the supernatant was carefully removed by inverting the cell culture plates. The cells were washed with phosphate buffer, and the washing solution was removed from the medium. The MTT solution (5 mg/mL) was mixed with culture medium at a 1:10 ratio. Then, 100 µL of the MTT mixture was added to each well, and the plates were incubated for 3 h at 37 °C, 5% CO_2_, and 95% humidity. At the end of the incubation period, the supernatant was removed, and 100 µL of DMSO was added to each well. After dissolution of the formed formazan crystals, the absorbance values of the wells were measured at 540 nm using an ELISA plate reader [3,24].

### 3.4. In Vitro EGFR Inhibition Assay

In vitro test kits used to evaluate EGFR tyrosine kinase inhibitor activities were obtained from BPS Bioscience (San Diego, CA, USA). Specifically, the EGFR Kinase Assay Kit (Cat. No. 40321), EGFR (L858R) Kinase Assay Kit (Cat. No. 40324), and EGFR (T790M/L858R) Kinase Assay Kit (Cat. No. 40322) were used. Inhibitor screening and profiling were performed using a Kinase-GloMAX-based method that allows the measurement of recombinant EGFR enzyme activity. Changes in enzyme activity were evaluated by adding compound **5a** at two different concentrations to the reaction mixture. The enzyme inhibition activity of compound **5a** was assessed according to the manufacturer’s protocol, and signal measurements were performed at the end of incubation using a microplate reader. Enzyme activity was calculated as percentage inhibition compared to the control group, and IC_50_ values were determined by nonlinear regression analysis applied to concentration–response curves [24,25,26,27].

### 3.5. ADME Predictions

The SwissADME program was used to retrospectively predict the pharmacokinetic and physicochemical properties of all compounds synthesized as benzimidazole–triazole derivatives. Using the program, drug-likeness properties (Lipinski, Ghose, Veber, Egan, Muegge), water solubility, gastrointestinal absorption, lipophilicity, and physicochemical properties were compared [28,29,30].

### 3.6. Molecular Docking Studies

Molecular docking studies were performed to investigate the binding interactions and binding site types of compound **5a**, the most potent EGFR inhibitor among the synthesized compounds, using the crystal structures of EGFR, EGFR-L858R, and EGFR-L858R/T790M (PDB IDs: 4HJO, 2ITZ, and 4I22, respectively) [31,32,33]. The Protein Preparation Wizard module in Schrödinger Suite 2015 Update 2 software was used to prepare the proteins for docking studies [34]. The preparation process involved optimizing protein structures, completing missing hydrogen atoms, determining appropriate ionization states, and applying the procedure described in our previous studies, including energy minimization. The charge distributions and binding parameters of the amino acid residues in the proteins were adjusted based on the OPLS 2005 force field. The three-dimensional structures and possible ionization states of the ligands were created using the LigPrep 3.8 module [35]. Subsequently, the docking grid encompassing the active site was defined using Glide 7.1 [36], and all molecular docking calculations were performed using the Standard Precision (SP) protocol [3,24].

### 3.7. Molecular Dynamic Studies

Molecular dynamics (MD) simulations, considered an important computational tool for evaluating the time-dependent stability of a ligand at an active site for a drug–receptor complex, were performed for 100 ns for compound **5a**, which was determined to be more active in this study. Molecular dynamics simulations were performed using the Desmond application with the TIP3P three-point water model, followed by energy minimization of the complexes using the OPLS3e force field implemented in the Schrödinger Suite. A final salt concentration of 0.15 M was maintained using Na^+^ and Cl^−^ ions to simulate physiological monovalent ion conditions while ensuring system neutralization. The simulations were conducted under NPT conditions at a constant temperature of 310.55 K and pressure of 1.01325 bar, using the RESPA integrator, NH thermostat for temperature control, and the MTK method for pressure regulation. Long-range electrostatic interactions were calculated using the Particle Mesh Ewald (PME) method, while short-range electrostatic and van der Waals interactions were calculated using a 9.0 Å cutoff distance. System stabilization was performed using the default protocol implemented in Desmond, consisting of a series of constrained minimization and molecular dynamics (MD) steps to gradually relax the system. The radius of gyration (Rg), root mean square fluctuation (RMSF), and root mean square deviation (RMSD) values were calculated using the Desmond program [3,24,37].

## 4. Conclusions

A novel series of benzimidazole–triazole hybrid derivatives has been designed, synthesized, and bioevaluated as potential EGFR tyrosine kinase inhibitors for the development of novel anticancer compounds. Cytotoxicity studies showed that compounds **5a**, **5c**, **5d**, and **5i** demonstrated significant activity against the A549 lung cancer cell line, with compound **5a** standing out due to its potent antiproliferative activity (IC_50_ = 6.014 ± 0.758 μM), exhibiting greater cytotoxic activity than doxorubicin. Furthermore, compound **5a** emerged as the most promising derivative due to its relatively lower cytotoxicity against NIH/3T3 cells. In vitro EGFR inhibition studies confirmed that compound **5a** exhibited remarkable inhibitory activity against EGFR, but its efficacy decreased progressively against the EGFR-L858R and EGFR-L858R/T790M mutant forms, showing higher selectivity against the unmutated receptor. ADME estimates revealed that all synthesized compounds possessed generally acceptable drug-like properties according to the Lipinski rules. Compound **5a** demonstrated good water solubility, high predictive gastrointestinal absorption, and favorable oral drug likeness. Molecular docking and molecular dynamics simulations supported the experimental findings, showing that compound **5a** formed stable interactions with key amino acid residues in the ATP-binding pocket of EGFR, particularly via persistent hydrogen bonds with THR766 and MET769, while less critical interactions were observed with mutant receptors. These computational results were consistent with enzymatic inhibition data and explained the decreased activity against mutant EGFR variants. Overall, the findings suggest that synthesized benzimidazole–triazole hybrids, particularly compound **5a**, may be a promising construct for the development of novel EGFR inhibitors targeting EGFR cancer cells.

## Figures and Tables

**Figure 1:**
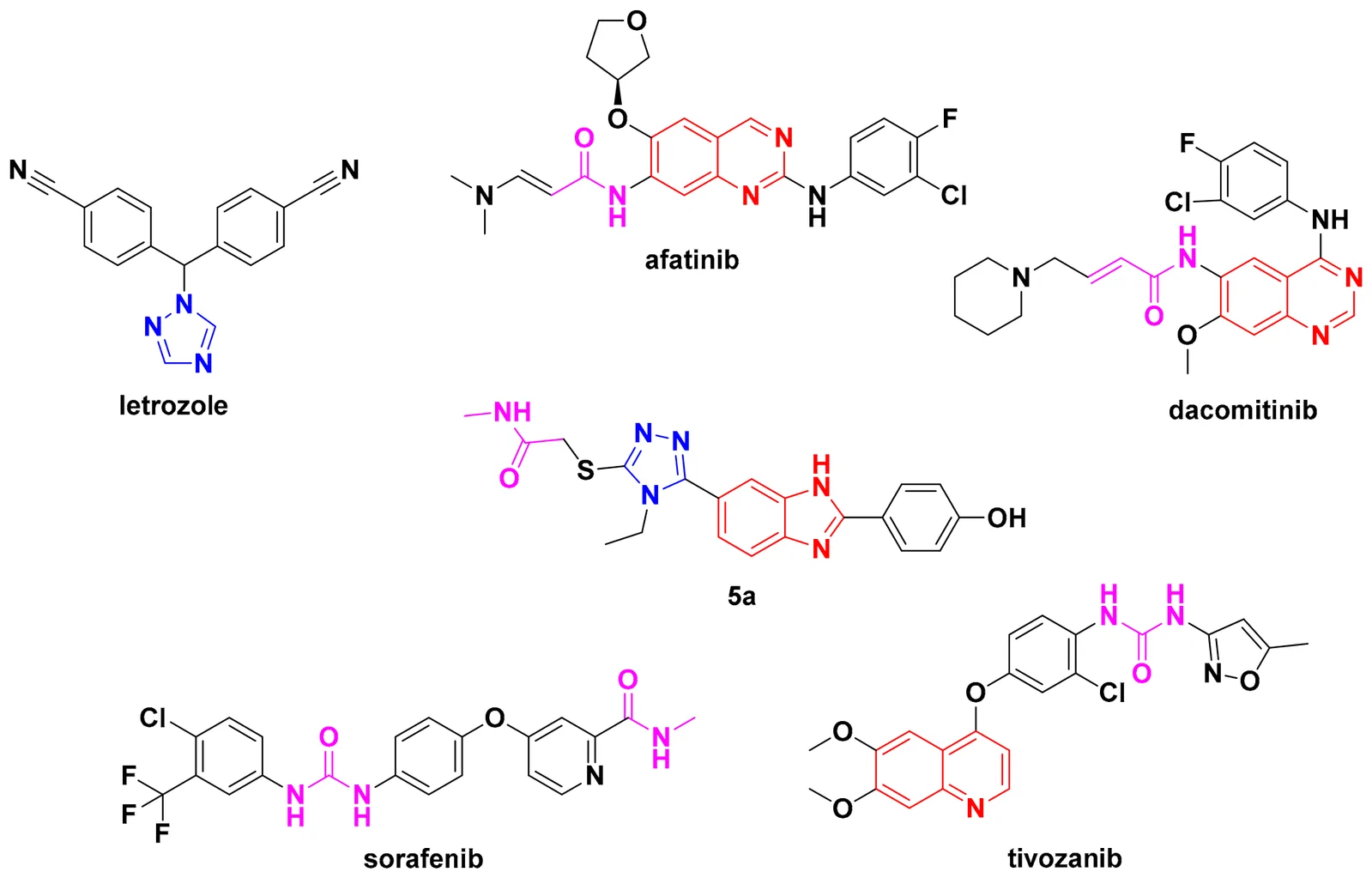
Comparison of the pharmacophoric regions of established anticancer drugs and the designed compound **5a** (blue: triazole moieties; red: benzimidazole/quinazoline-related heteroaromatic moieties; purple: amide/urea moieties; colors indicate selected structural features for visual comparison).

**Scheme 1:**
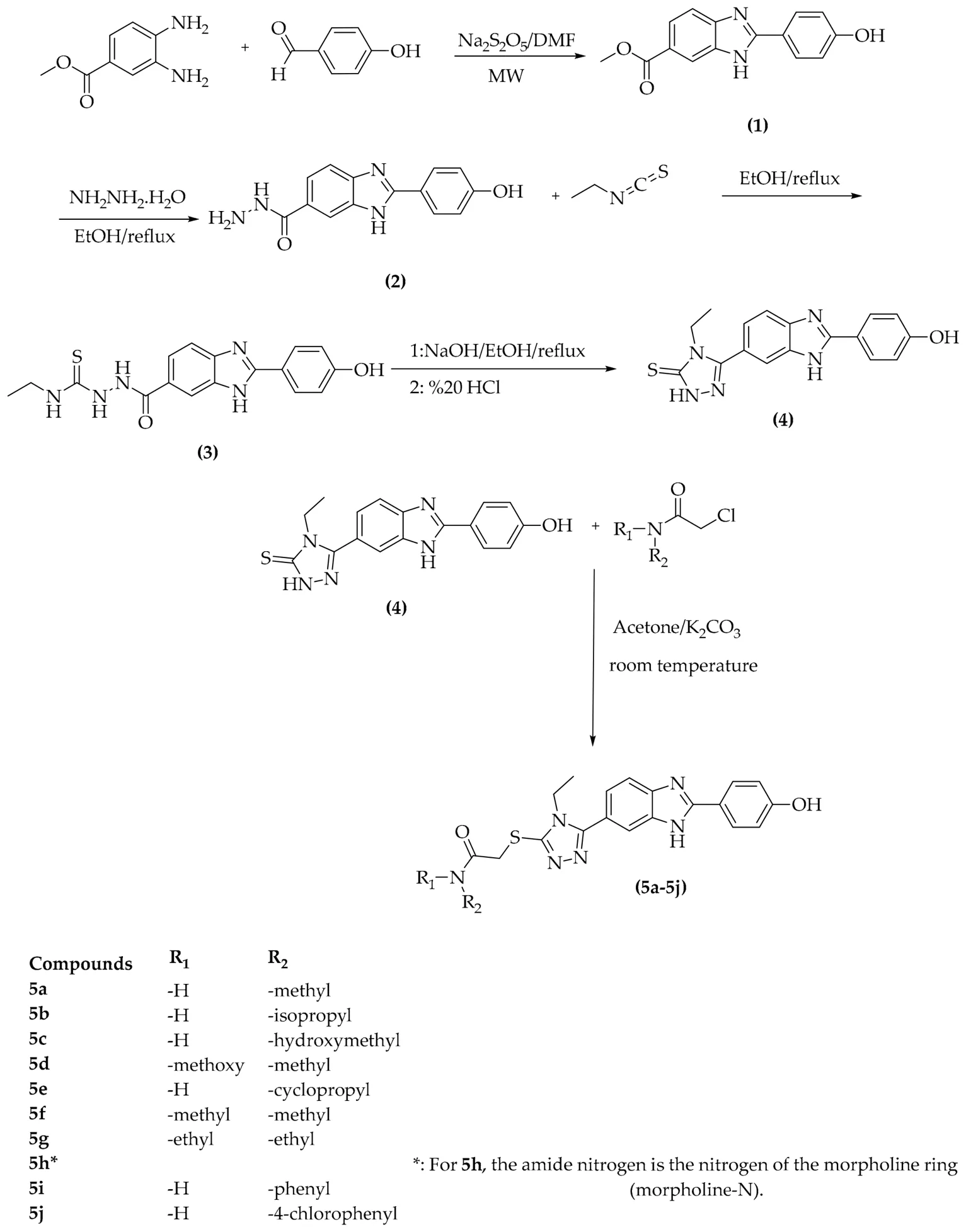
Substituents and synthesis method of the designed compounds (**5a**–**5j**).

**Figure 2:**
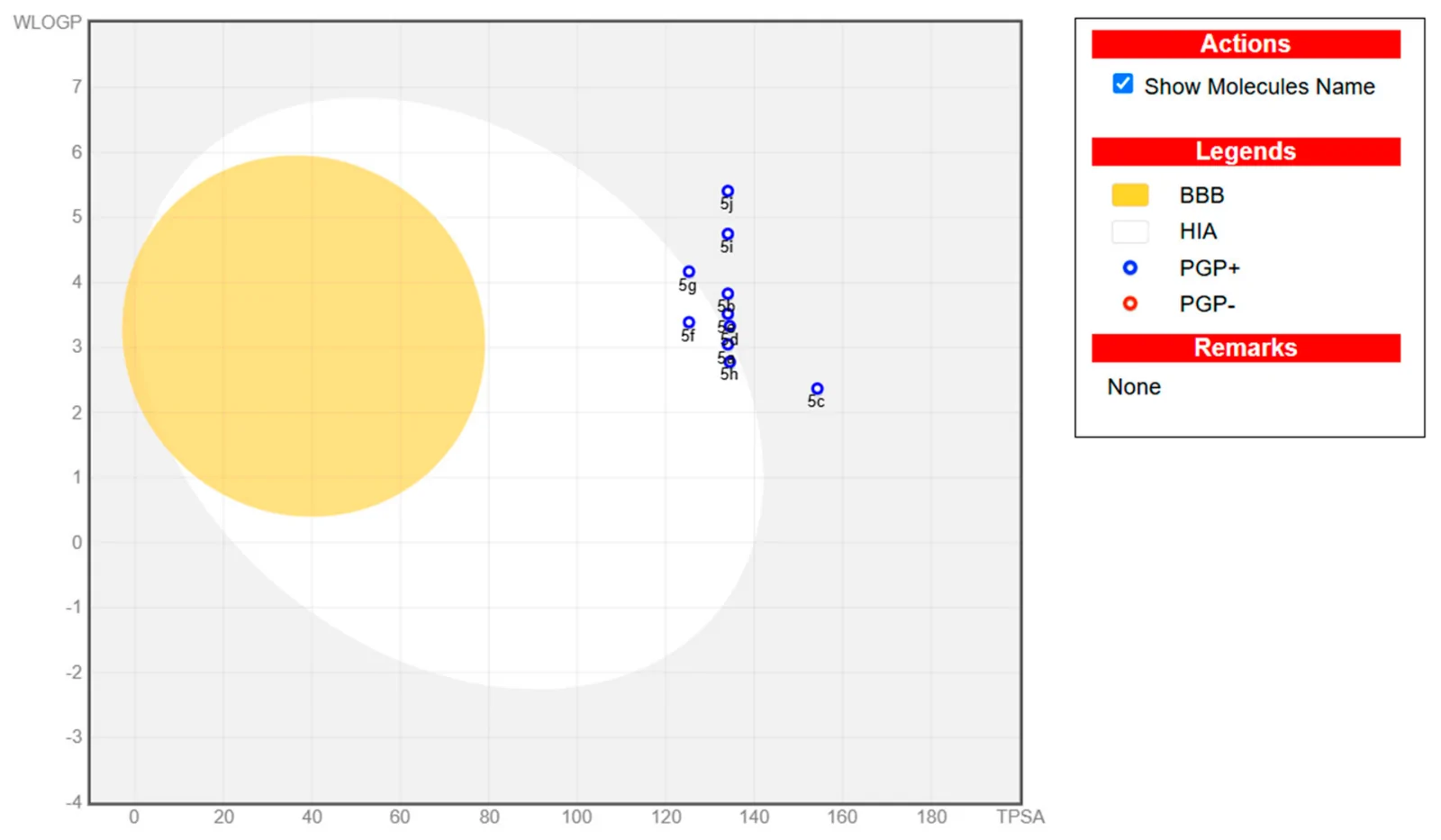
The BOILED-Egg model of all compounds.

**Figure 3:**
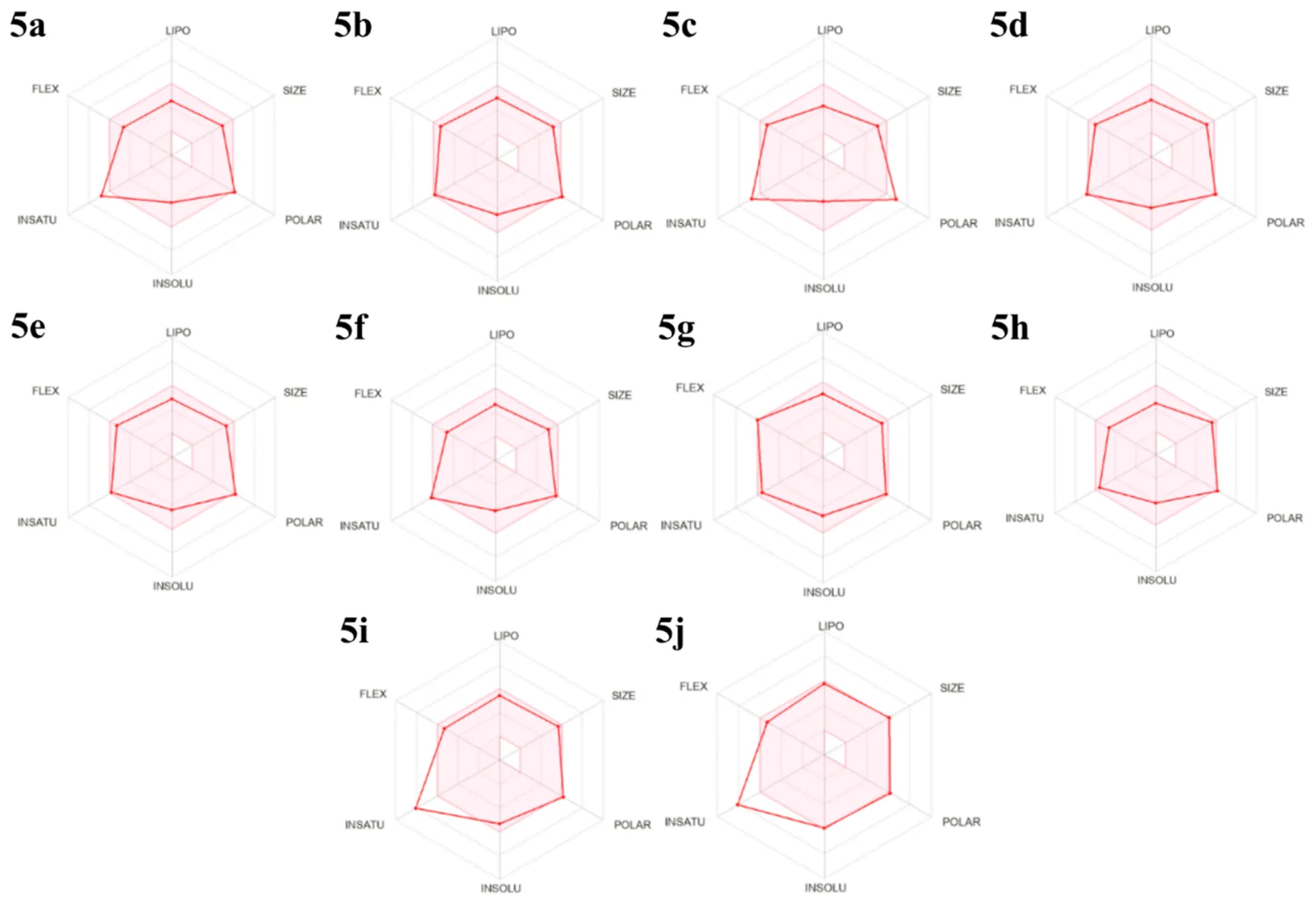
Predicted physicochemical space for oral bioavailability.

**Figure 4:**
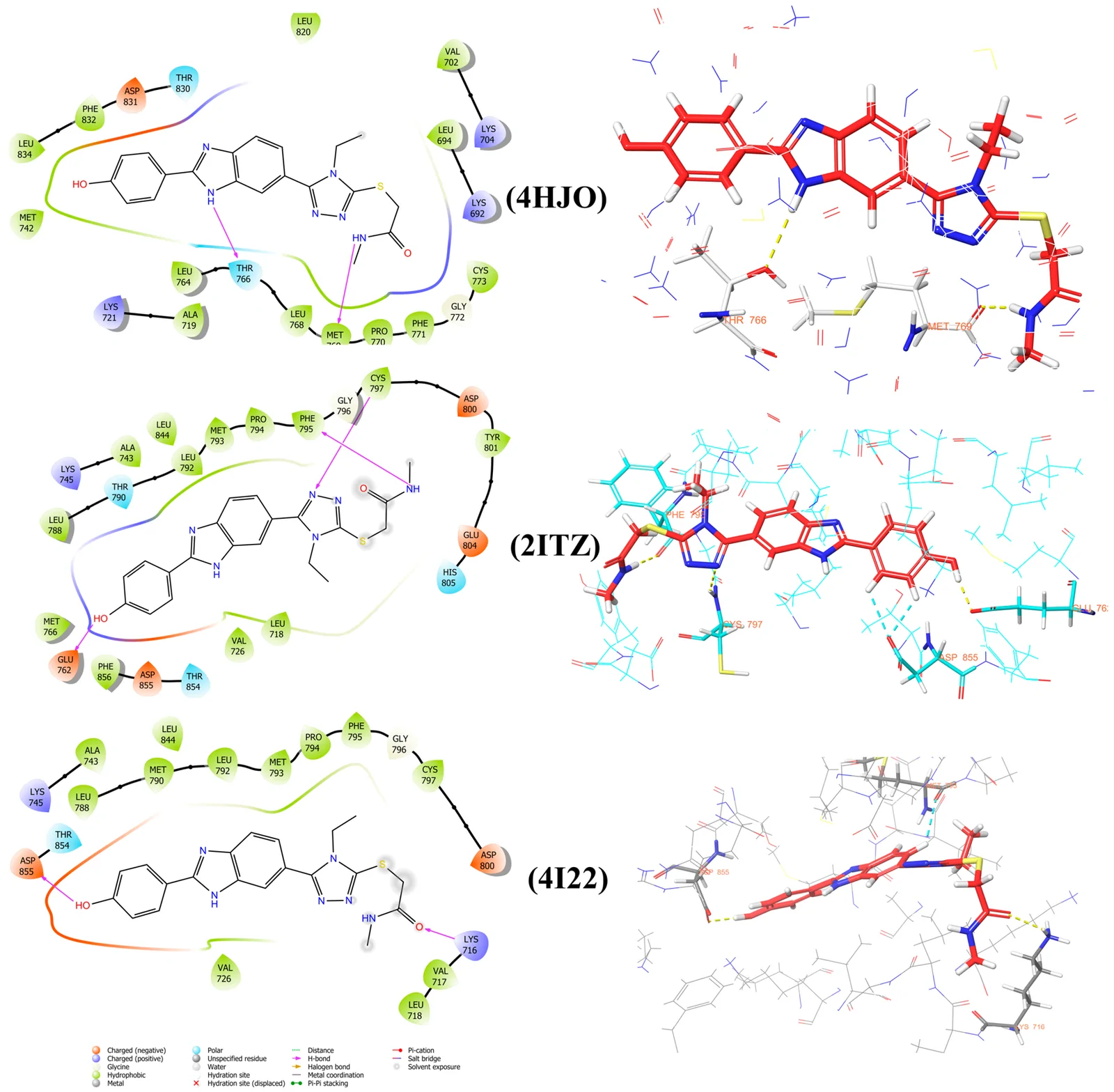
3D and 2D poses of compound **5a** in the active region of EGFR (PDB ID: 4HJO), EGFR-L858R (PDB ID: 2ITZ) and EGFR-L858R/T790M (PDB ID: 4I22).

**Figure 5:**
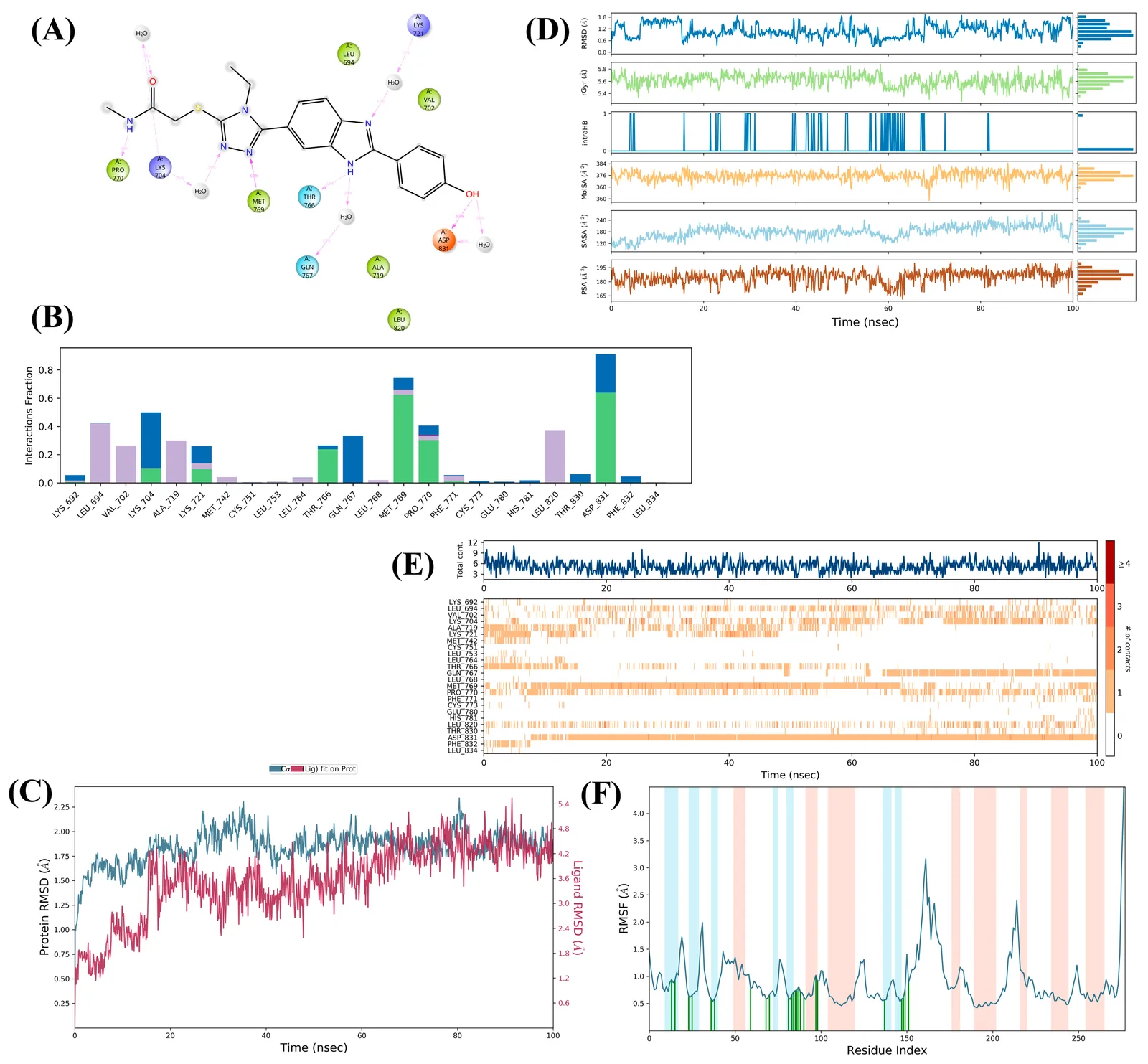
MD simulation stability results of compound **5a**-EGFR (PDB ID: 4HJO) complex. (**A**) Ligand–protein contacts 2D summary, (**B**) types of interactions, (**C**) RMSD parameters, (**D**) ligand properties, (**E**) time-dependent amino acid interactions, (**F**) RMSF parameters.

**Figure 6:**
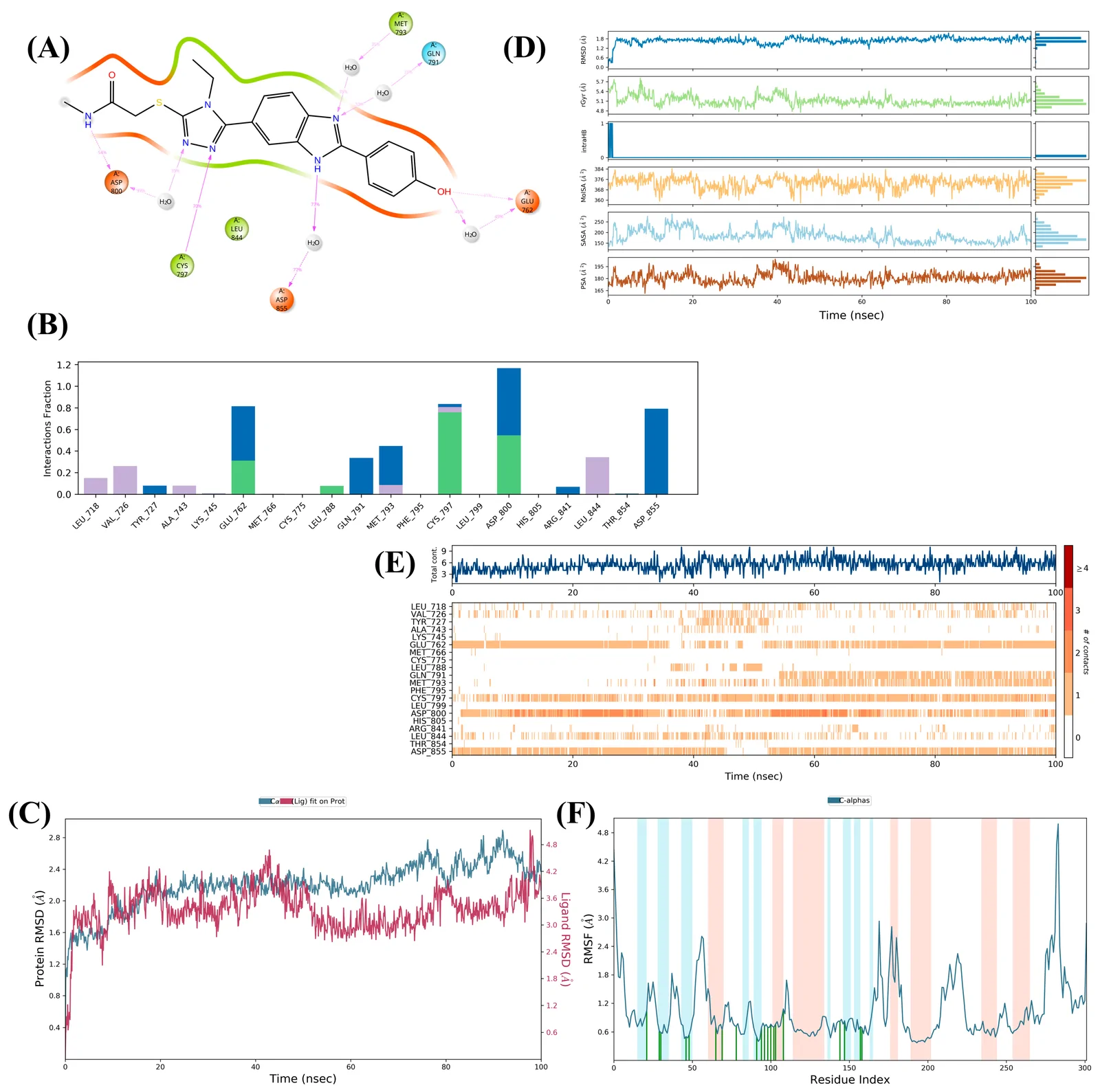
MD simulation stability results of compound **5a**-EGFR-L858R (PDB ID: 2ITZ) complex. (**A**) Ligand–protein contacts 2D summary, (**B**) types of interactions, (**C**) RMSD parameters, (**D**) ligand properties, (**E**) time-dependent amino acid interactions, (**F**) RMSF parameters.

**Figure 7:**
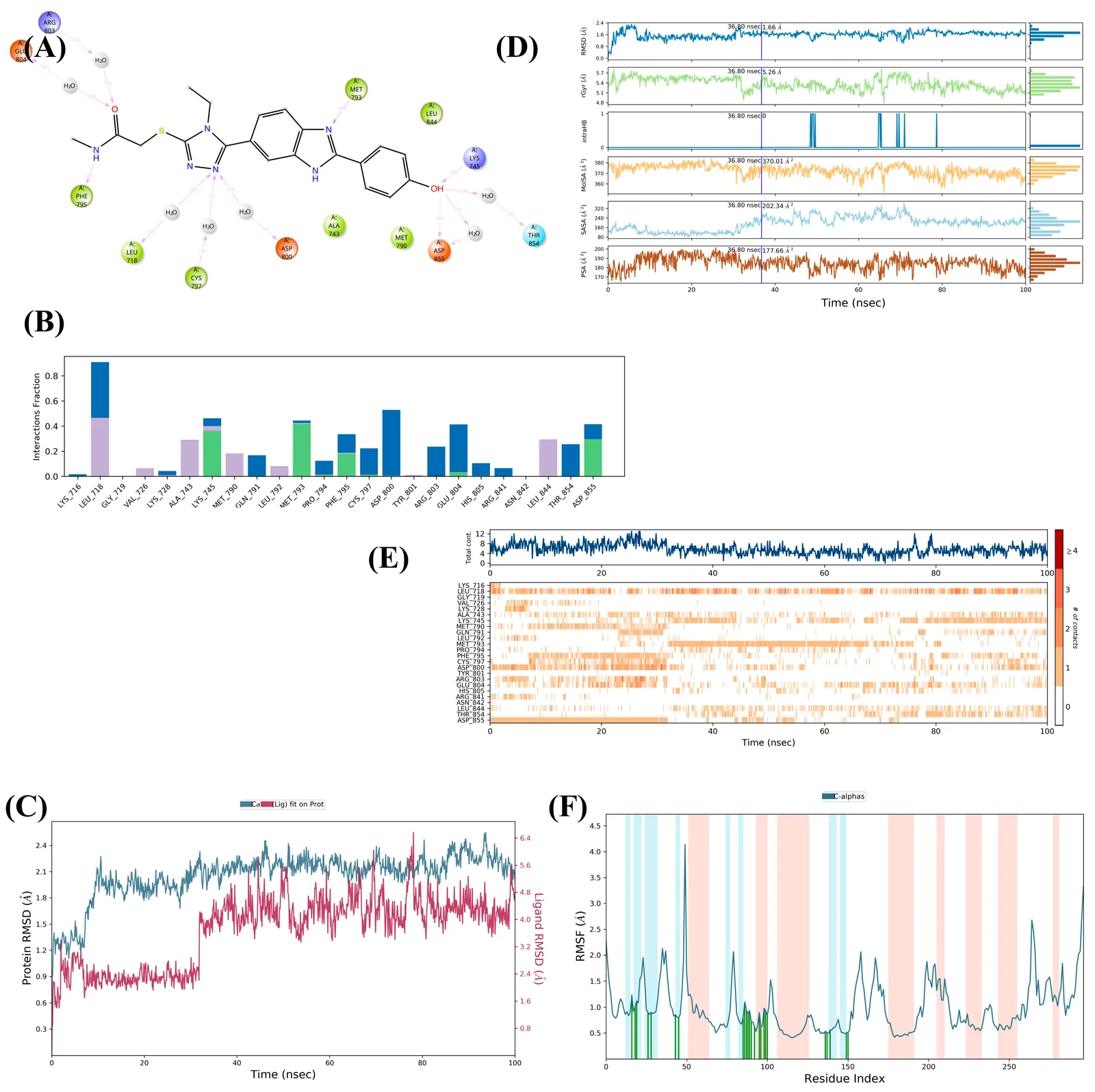
MD simulation stability results of compound **5a**-EGFR-L858R/T790M (PDB ID: 4I22) complex. (**A**) Ligand–protein contacts 2D summary, (**B**) types of interactions, (**C**) RMSD parameters, (**D**) ligand properties, (**E**) time-dependent amino acid interactions, (**F**) RMSF parameters.

**Table 1:** IC_50_ (µM) values of synthesized compounds and doxorubicine against A549 and NIH/3T3 cell lines.

Compounds	A549 (μM)	NIH/3T3 (μM)
**5a**	6.01 ± 0.76	12.43 ± 0.87
**5b**	368.62 ± 2.09	501.61 ± 2.06
**5c**	25.04 ± 0.99	182.77 ± 1.04
**5d**	86.39 ± 1.06	253.65 ± 2.91
**5e**	152.01 ± 2.88	592.45 ± 4.00
**5f**	162.83 ± 1.99	>1000
**5g**	>1000	>1000
**5h**	615.76 ± 6.09	214.86 ± 1.06
**5i**	71.35 ± 3.65	38.74 ± 0.77
**5j**	808.22 ± 3.08	>1000
Doxorubicine	9.14 ± 0.18	>1000

**Table 2:** % Inhibition and IC_50_ (µM) values of compound **5a** and Erlotinib against EGFR, EGFR-L858R, EGFR-L858R/T790M.

EGFR Types	Compound 5a IC_50_ (µM)	Erlotinib IC_50_ (µM)
EGFR	0.068 ± 0.008	0.029 ± 0.007
EGFR-L858R	0.269 ± 0.011	0.066 ± 0.015
EGFR-L858R/T790M	5.267 ± 0.109	0.590 ± 0.092

**Table 3:** Drug likeness, water solubility and pharmacokinetic properties of all compounds.

Comp.	Drug Likeness					Water Solubility		Pharmacokinetics	
	Lipinski	Ghose	Veber	Egan	Muegge	LogS	Class	GI abs.	F
**5a**	+	+	+	-	+	−3.96	Soluble	High	0.55
**5b**	+	+	+	-	+	−4.54	Moderately	Low	0.55
**5c**	+	+	-	-	-	−3.61	Soluble	Low	0.55
**5d**	+	+	+	-	+	−4.20	Moderately	Low	0.55
**5e**	+	+	+	-	+	−4.38	Moderately	Low	0.55
**5f**	+	+	+	+	+	−4.15	Moderately	High	0.55
**5g**	+	+	+	+	+	−4.63	Moderately	Low	0.55
**5h**	+	-	+	-	+	−4.13	Moderately	High	0.55
**5i**	+	-	+	-	+	−5.32	Moderately	Low	0.55
**5j**	+	-	+	-	+	−5.92	Moderately	Low	0.55

**Table 4:** The physicochemical and lipophilicity properties of all compounds.

Comp.	Physicochemical Properties							Lipophilicity
	MW	Fsp3	RB	HBA	HBD	MR	TPSA	cLogP
**5a**	404.48	0.20	7	5	3	112.97	134.02	2.38
**5b**	436.53	0.27	8	5	3	122.59	134.02	3.04
**5c**	424.48	0.20	8	6	4	114.14	154.25	1.72
**5d**	438.50	0.24	8	6	2	118.96	134.46	2.44
**5e**	434.51	0.27	8	5	3	120.47	134.02	2.86
**5f**	422.50	0.24	7	5	2	117.88	125.23	2.55
**5g**	450.56	0.30	9	5	2	127.49	125.23	3.24
**5h**	464.54	0.30	7	6	2	130.38	134.46	2.27
**5i**	470.55	0.12	8	5	3	134.19	134.02	3.74
**5j**	504.99	0.12	8	5	3	139.20	134.02	4.34

Comp: compounds, MW: molecular weight, Fsp3: fraction Csp3, RB: number of rotatable bonds, HBA: number of hydrogen bond acceptors, HBD: number of hydrogen bond donors, MR: molar refractivity, TPSA: total polar surface area, cLogP: consensus Log P (average of five predictions).

## Data Availability

Dataset is available on request from the authors.

## Supplementary Materials

The following supporting information can be downloaded at: https://www.mdpi.com/article/10.3390/molecules31193433/s1; the ^1^H-NMR, ^13^C-NMR, and HRMS spectrums; HPLC chromatograms; dose–response curves; molecular docking and molecular dynamic poses of compounds **5a**–**5j**; HSQC and HMBC spectrums of compound **5f** are available online.
