# Supplementary material for: Design, Synthesis, In Vitro and In Silico Evaluation of Triazole–Benzimidazole Hybrid Derivatives as Potential Inhibitors of EGFR, EGFR-L858R, and EGFR-L858R/T790M
Source: Molecules. 2026 Sep 27;31(19):3433. doi: 10.3390/molecules31193433 (PMC13666104; doi:10.3390/molecules31193433)
Supplement: Supplementary file 1 [file molecules-31-03433-s001.zip › molecules-4543585-supplementary.pdf]

Design, Synthesis, In Vitro and In Silico Evaluation of Triazole–Benzimidazole Hybrid Derivatives as Potential Inhibitors of EGFR, EGFR-L858R, and EGFR-L858R/T790M

Harun Uslu [1,*], Arzu Hıdır [2,3], Esra Yumuşak [4], Derya Osmaniye [2,5], Bünyamin Göktaş [1,3], Yusuf Özkay [2,5] and Zafer Asım Kaplancıklı [2,5]

[1] Department of Pharmaceutical Chemistry, Faculty of Pharmacy, Fırat University, 23119 Elazığ, Türkiye
[2] Department of Pharmaceutical Chemistry, Faculty of Pharmacy, Anadolu University, 26470 Eskişehir, Türkiye
[3] Department of Pharmaceutical Chemistry, The Institute of Graduate Education, Anadolu University, 26470 Eskişehir, Türkiye
[4] Faculty of Pharmacy, Anadolu University, 26470 Eskişehir, Türkiye;
[5] Central Research Laboratory, Faculty of Pharmacy, Anadolu University, 26470 Eskişehir, Türkiye
* Correspondence: huslu@firat.edu.tr; Tel.: +90-4246076703; Fax: +90-4242411226

2-((4-Ethyl-5-(2-(4-hydroxyphenyl)-1H-benzo[d]imidazol-6-yl)-4H-1,2,4-triazol-3-yl)thio)-N-methylacetamide ***(5a)***

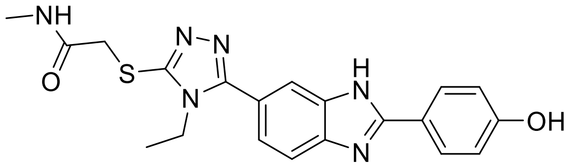

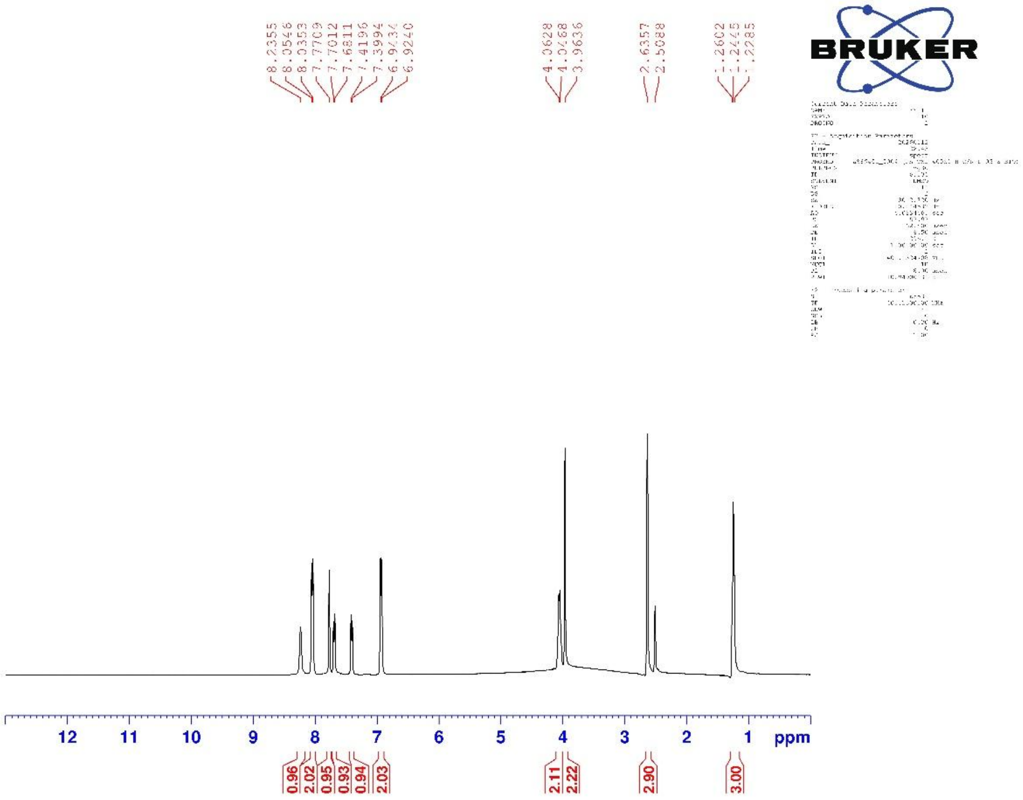

Figure S1. Compound **5a** ^{1}H-NMR spectrum.

2-((4-Ethyl-5-(2-(4-hydroxyphenyl)-1H-benzo[d]imidazol-6-yl)-4H-1,2,4-triazol-3-yl)thio)-N-methylacetamide ***(5a)***

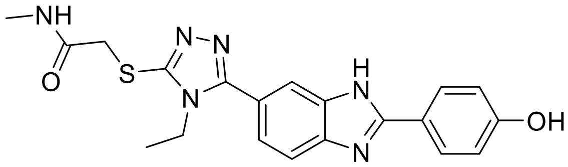

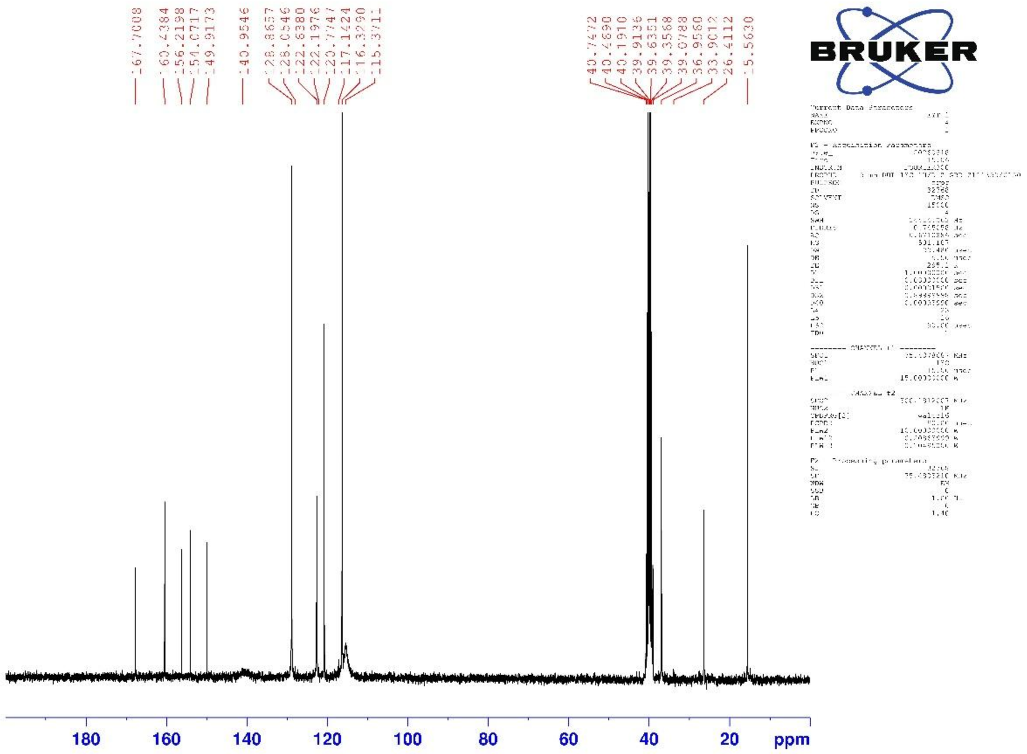

Figure S2. Compound **5a** ^{13}C-NMR spectrum.

2-((4-Ethyl-5-(2-(4-hydroxyphenyl)-1H-benzo[d]imidazol-6-yl)-4H-1,2,4-triazol-3-yl)thio)-N-methylacetamide ***(5a)***

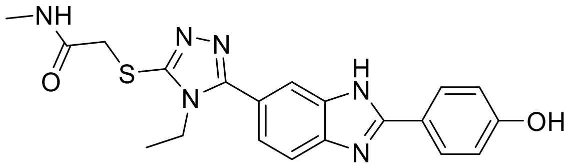

Elmt	Val.	Min	Max	Elmt	Val.	Min	Max	Elmt	Val.	Min	Max	Elmt	Val.	Min	Max	Use Adduct
H	1	10	30	O	2	0	4	S	2	1	1	Ru	2	0	0	H
C	4	12	30	F	1	0	0	Cl	1	0	1	Pd	2	0	0	
N	3	2	6	P	3	0	0	Br	1	0	0	I	3	0	0	

Error Margin (ppm):	10	DBE Range:	10.0 - 20.0	Electron Ions:	both
HC Ratio:	unlimited	Apply N Rule:	yes	Use MSn Info:	yes
Max Isotopes:	5	Isotope RI (%):	1.00	Isotope Res:	9000
MSn Iso RI (%):	10.00	MSn Logic Mode:	AND	Max Results:	50

Event#: 1 MS(E+) Ret. Time : 1.240 Scan# : 187

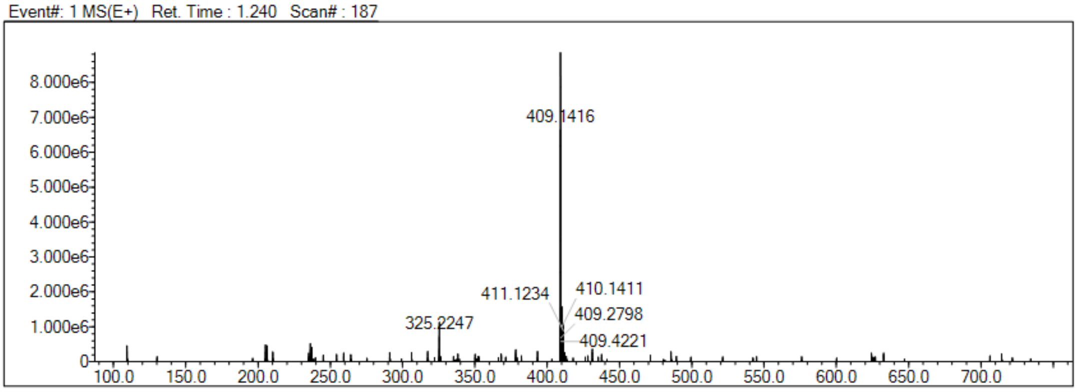

Measured region for 409.1416 m/z

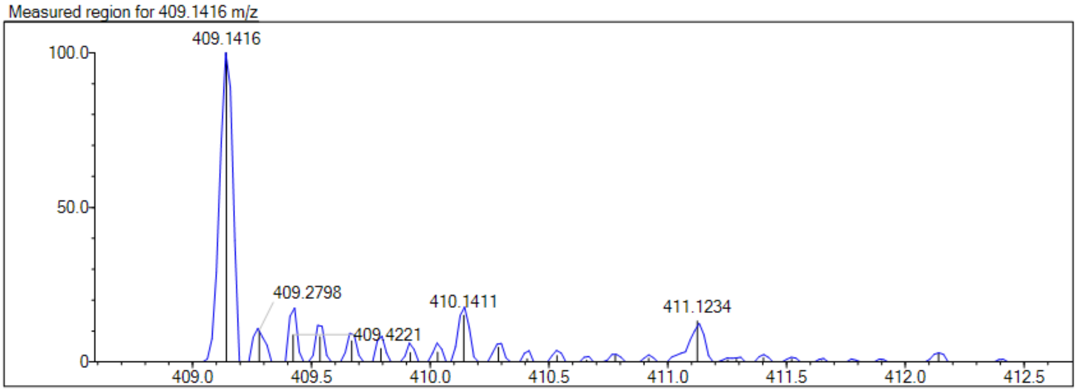

C20 H20 N6 O2 S [M+H]+ : Predicted region for 409.1441 m/z

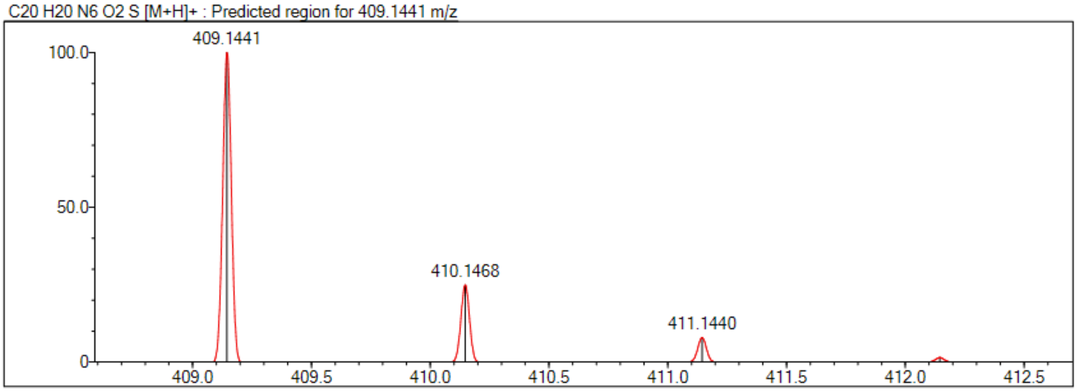

Rank	Score	Formula (M)	Ion	Meas. m/z	Pred. m/z	Df. (mDa)	Df. (ppm)	Iso	DBE
1	41.52	C20 H20 N6 O2 S	[M+H]+	409.1416	409.1441	-2.5	-6.11	52.63	14.0

Figure S3. Compound **5a** HRMS spectrum.

2-((4-ethyl-5-(2-(4-hydroxyphenyl)-1H-benzo[d]imidazol-6-yl)-4H-1,2,4-triazol-3-yl)thio)-N-isopropylacetamide ***(5b)***

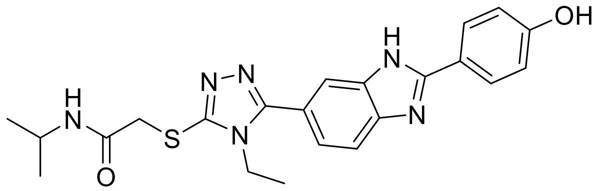

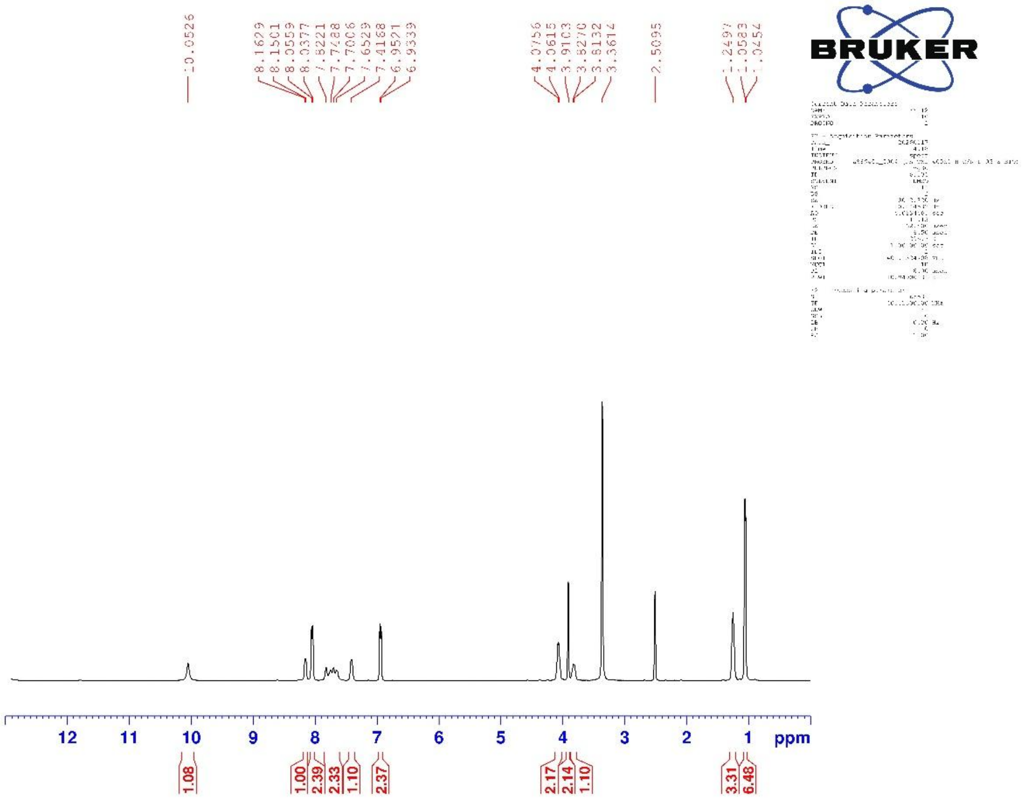

Figure S4. Compound **5b** ^{1}H-NMR spectrum.

2-((4-ethyl-5-(2-(4-hydroxyphenyl)-1H-benzo[d]imidazol-6-yl)-4H-1,2,4-triazol-3-yl)thio)-N-isopropylacetamide ***(5b)***

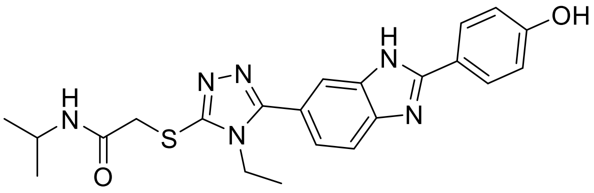

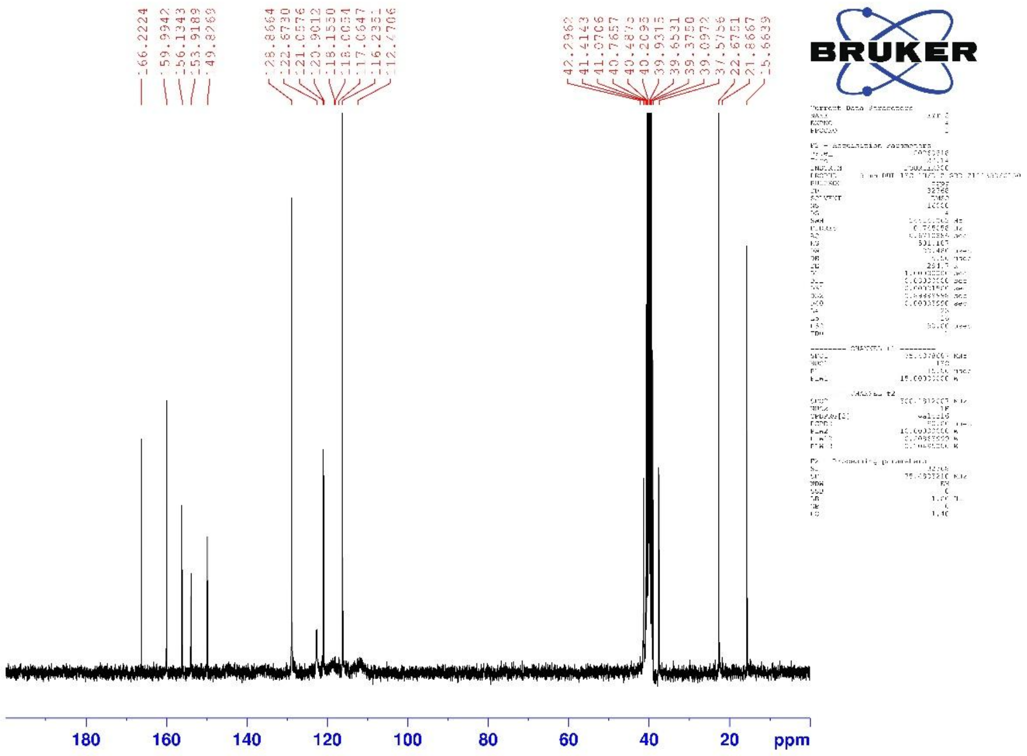

Figure S5. Compound **5b** ^{13}C-NMR spectrum.

2-((4-ethyl-5-(2-(4-hydroxyphenyl)-1H-benzo[d]imidazol-6-yl)-4H-1,2,4-triazol-3-yl)thio)-N-isopropylacetamide ***(5b)***

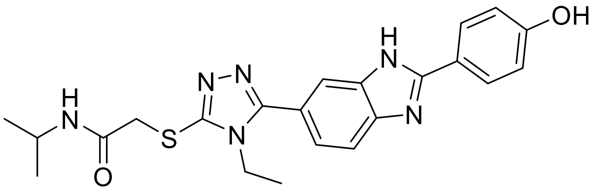

Elmt	Val.	Min	Max	Elmt	Val.	Min	Max	Elmt	Val.	Min	Max	Elmt	Val.	Min	Max	Use Adduct
H	1	10	30	O	2	0	4	S	2	1	1	Ru	2	0	0	H
C	4	12	30	F	1	0	0	Cl	1	0	1	Pd	2	0	0	
N	3	2	6	P	3	0	0	Br	1	0	0	I	3	0	0	

Error Margin (ppm): 5
HC Ratio: unlimited
Max Isotopes: 5
MSn Iso RI (%): 10.00

DBE Range: 10.0 - 20.0
Apply N Rule: yes
Isotope RI (%): 1.00
MSn Logic Mode: AND

Electron Ions: both
Use MSn Info: yes
Isotope Res: 9000
Max Results: 50

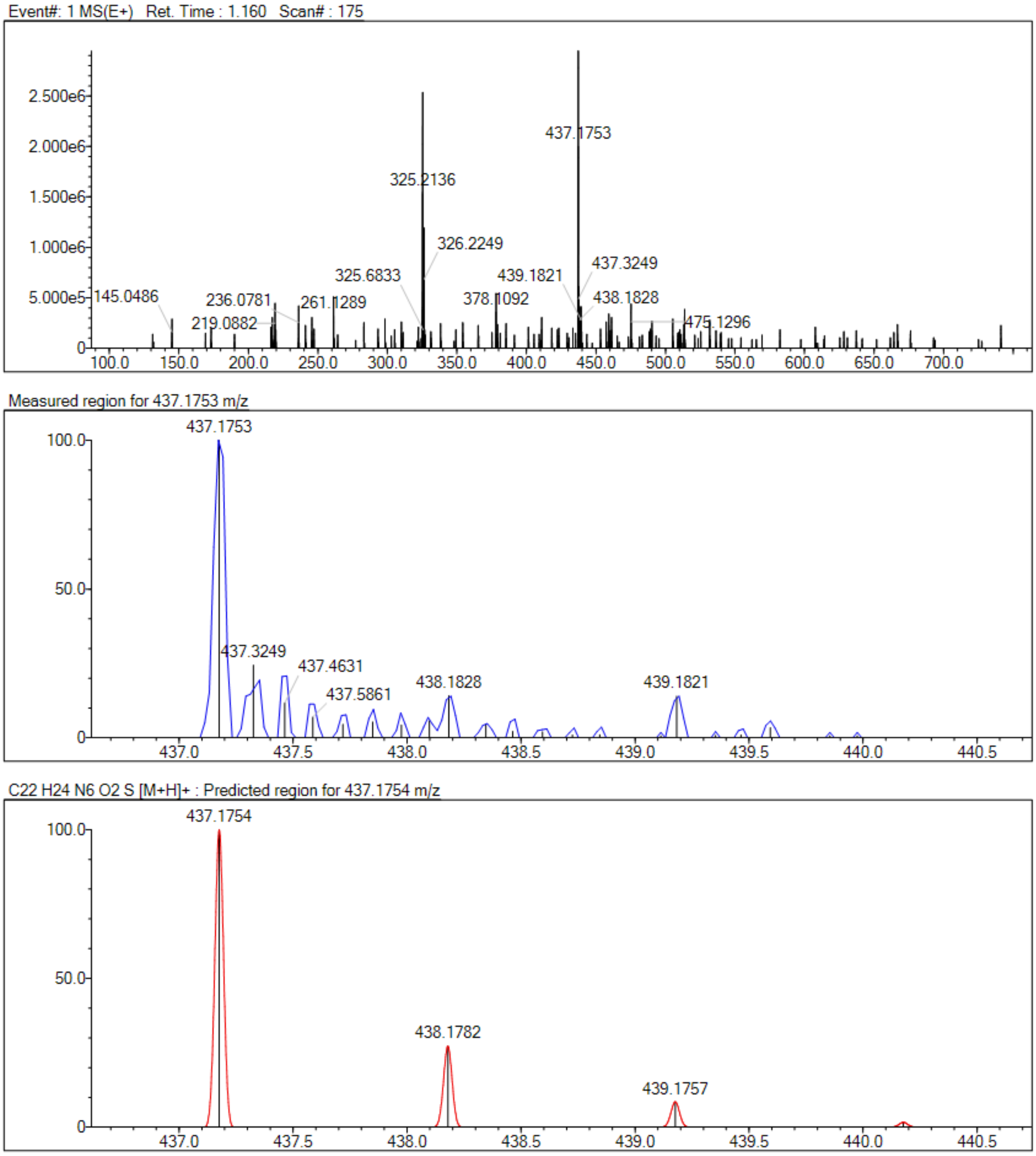

Rank	Score	Formula (M)	Ion	Meas. m/z	Pred. m/z	Df. (mDa)	Df. (ppm)	Iso	DBE
1	44.63	C22 H24 N6 O2 S	[M+H]+	437.1753	437.1754	-0.1	-0.23	44.63	14.0

Figure S6. Compound **5b** HRMS spectrum.

2-((4-ethyl-5-(2-(4-hydroxyphenyl)-1H-benzo[d]imidazol-6-yl)-4H-1,2,4-triazol-3-yl)thio)-N-(hydroxymethyl)acetamide ***(5c)***

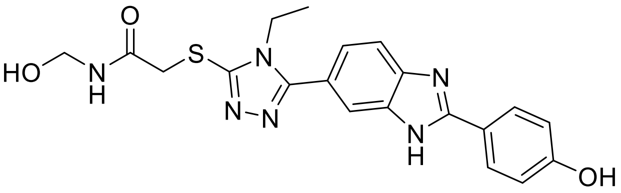

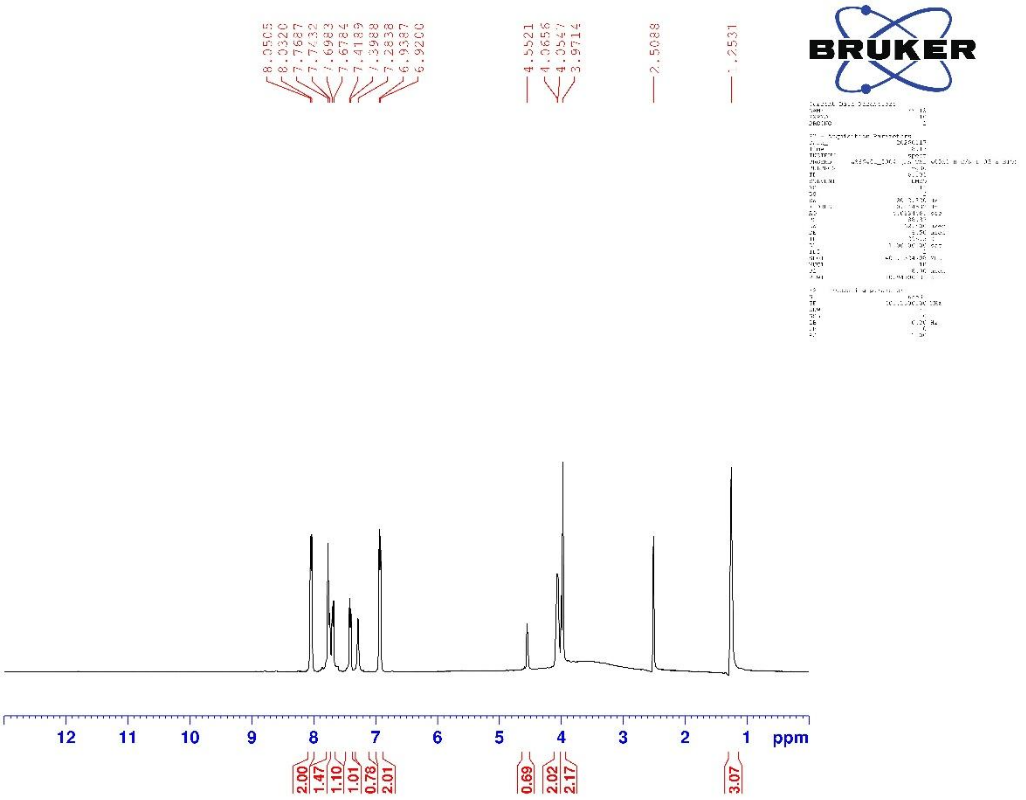

Figure S7. Compound **5c** ^{1}H-NMR spectrum.

2-((4-ethyl-5-(2-(4-hydroxyphenyl)-1H-benzo[d]imidazol-6-yl)-4H-1,2,4-triazol-3-yl)thio)-N-(hydroxymethyl)acetamide ***(5c)***

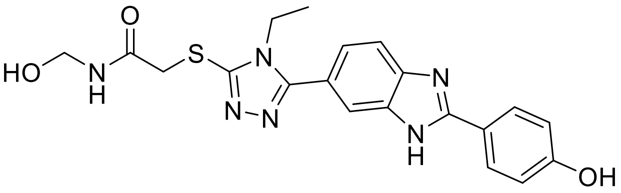

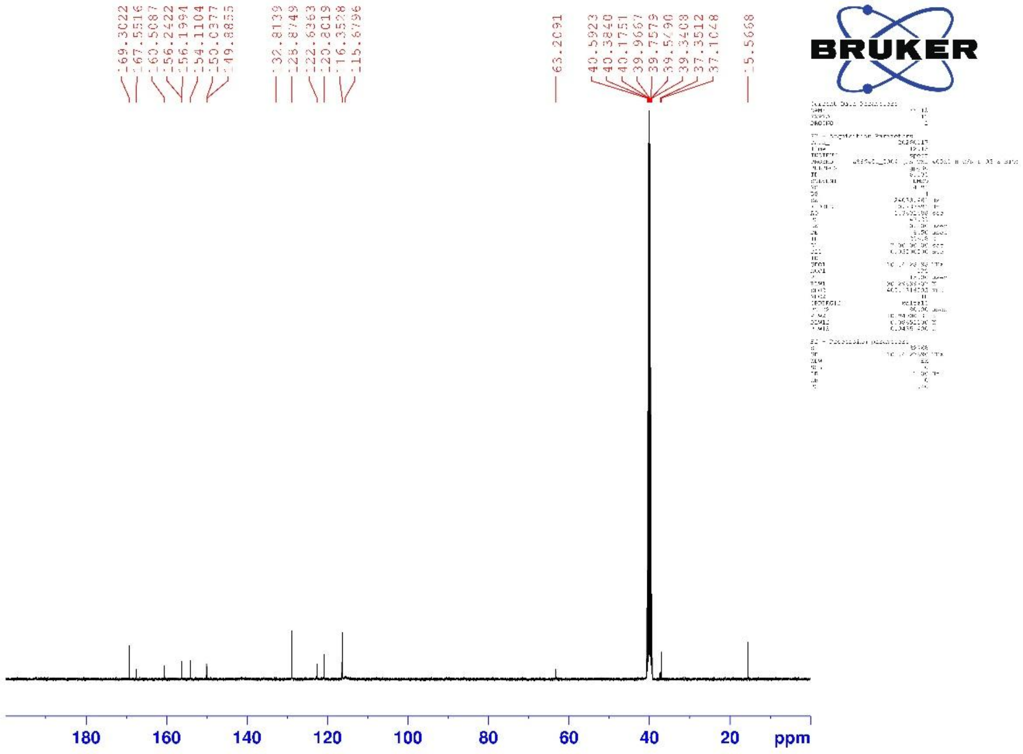

Figure S8. Compound **5c** ^{13}C-NMR spectrum.

2-((4-ethyl-5-(2-(4-hydroxyphenyl)-1H-benzo[d]imidazol-6-yl)-4H-1,2,4-triazol-3-yl)thio)-N-(hydroxymethyl)acetamide **(5c)**

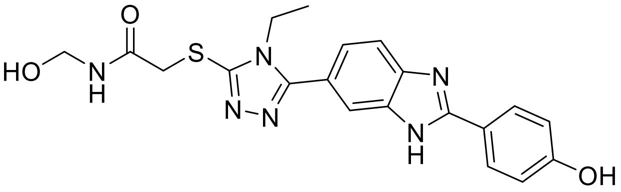

Elmt	Val.	Min	Max	Elmt	Val.	Min	Max	Elmt	Val.	Min	Max	Elmt	Val.	Min	Max	Use Adduct
H	1	10	30	O	2	0	4	S	2	1	1	Ru	2	0	0	H
C	4	12	30	F	1	0	0	Cl	1	0	1	Pd	2	0	0	
N	3	2	6	P	3	0	0	Br	1	0	0	I	3	0	0	

Error Margin (ppm):	5	DBE Range:	10.0 - 20.0	Electron Ions:	both
HC Ratio:	unlimited	Apply N Rule:	yes	Use MSn Info:	yes
Max Isotopes:	5	Isotope RI (%):	1.00	Isotope Res:	9000
MSn Iso RI (%):	10.00	MSn Logic Mode:	AND	Max Results:	50

Event#: 1 MS(E+) Ret. Time : 1.080 Scan# : 163

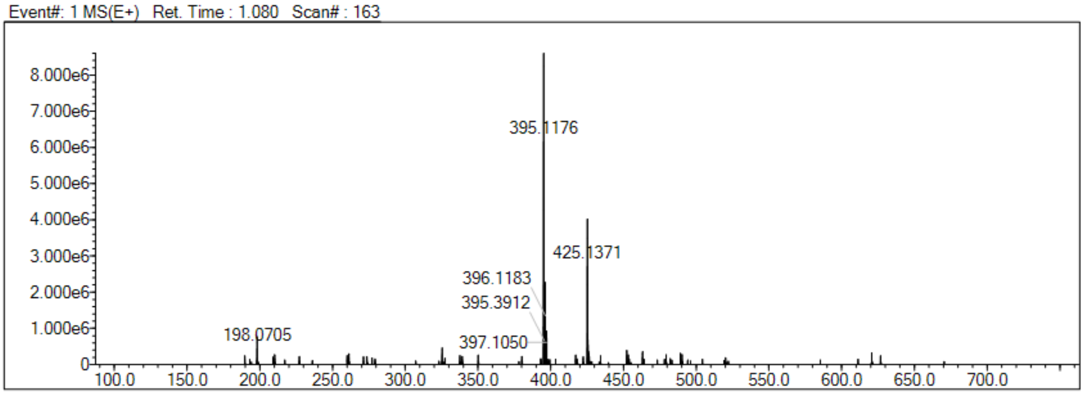

Measured region for 425.1371 m/z

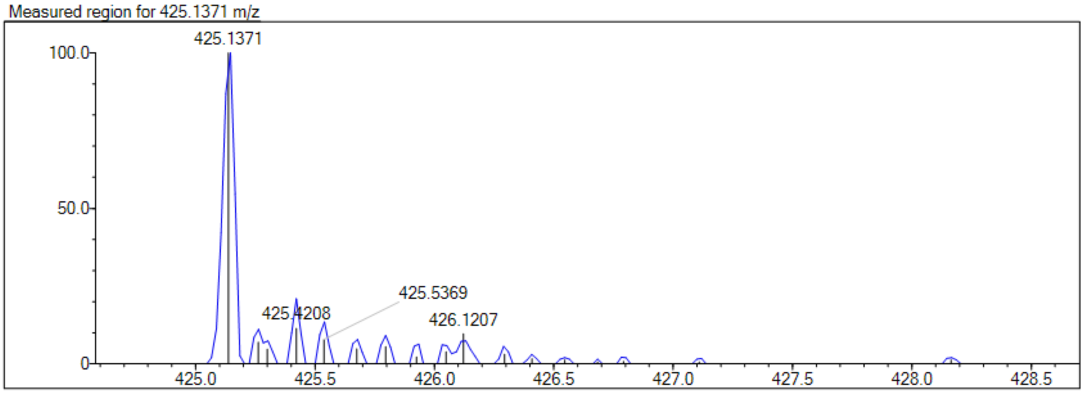

C20 H20 N6 O3 S [M+H]+ : Predicted region for 425.1390 m/z

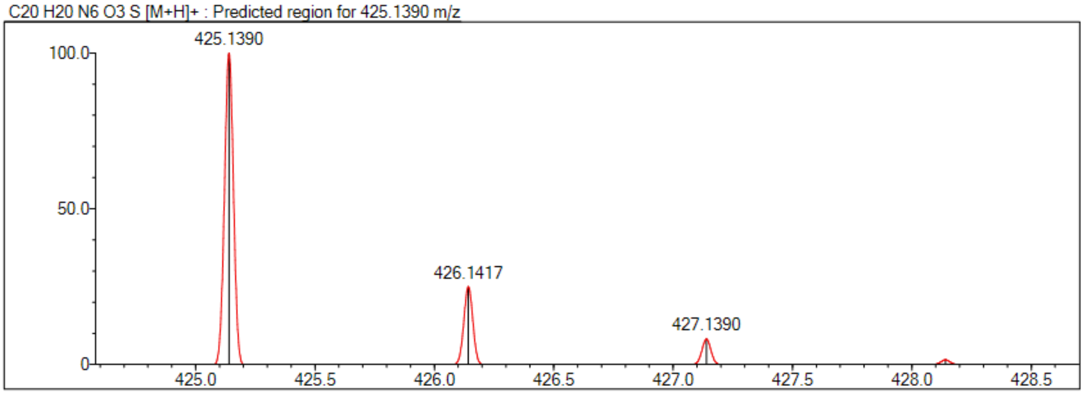

Rank	Score	Formula (M)	Ion	Meas. m/z	Pred. m/z	Df. (mDa)	Df. (ppm)	Iso	DBE
1	20.53	C20 H20 N6 O3 S	[M+H]+	425.1371	425.1390	-1.9	-4.47	22.48	14.0

Figure S9. Compound **5c** HRMS spectrum.

2-((4-Ethyl-5-(2-(4-hydroxyphenyl)-1H-benzo[d]imidazol-6-yl)-4H-1,2,4-triazol-3-yl)thio)-N-methoxy-N-methylacetamide ***(5d)***

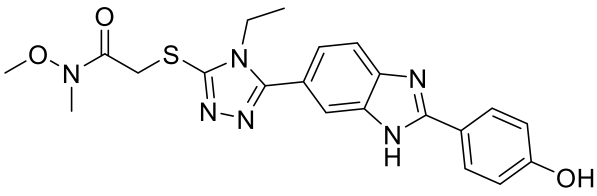

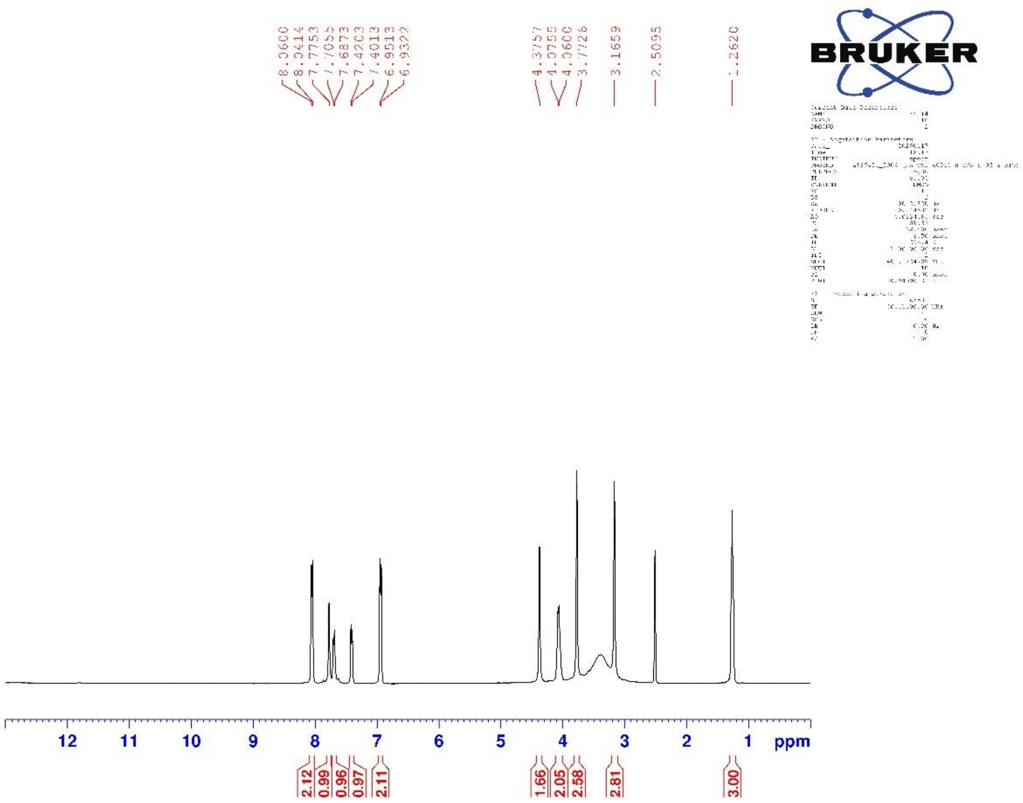

Figure S10. Compound **5d** ^{1}H-NMR spectrum.

2-((4-Ethyl-5-(2-(4-hydroxyphenyl)-1H-benzo[d]imidazol-6-yl)-4H-1,2,4-triazol-3-yl)thio)-N-methoxy-N-methylacetamide ***(5d)***

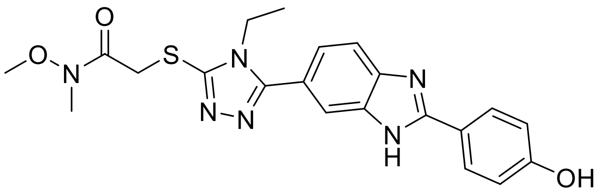

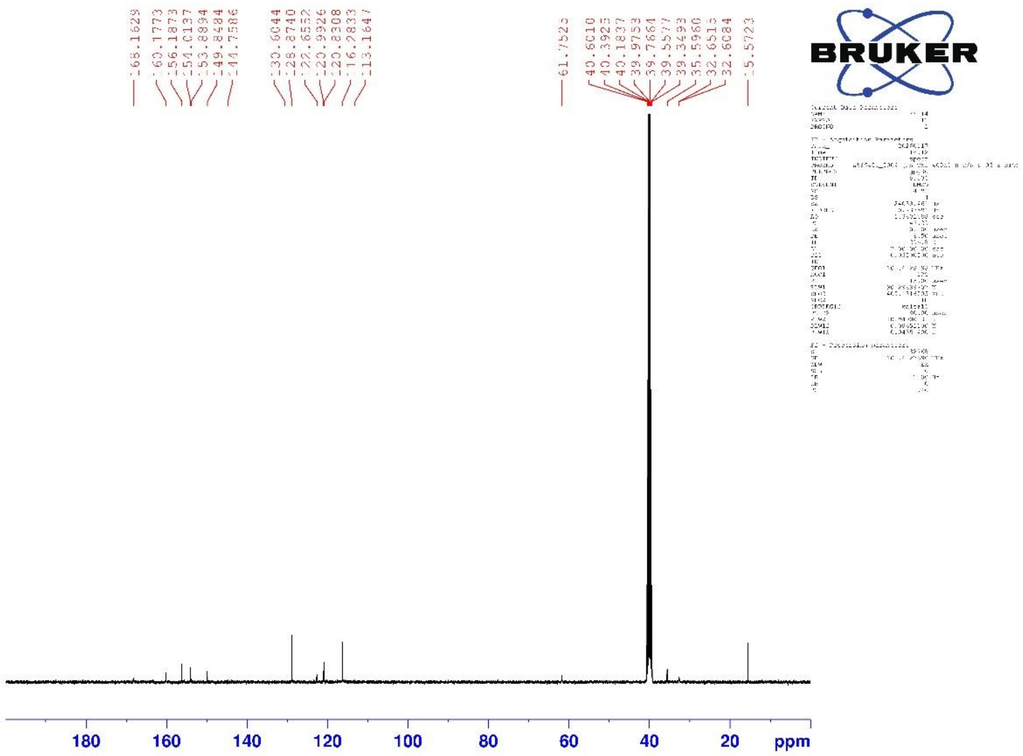

Figure S11. Compound **5d** ^{13}C-NMR spectrum.

2-((4-Ethyl-5-(2-(4-hydroxyphenyl)-1H-benzo[d]imidazol-6-yl)-4H-1,2,4-triazol-3-yl)thio)-N-methoxy-N-methylacetamide ***(5d)***

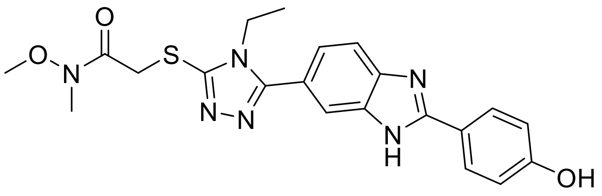

Elmt	Val.	Min	Max	Elmt	Val.	Min	Max	Elmt	Val.	Min	Max	Elmt	Val.	Min	Max	Use Adduct
H	1	10	30	O	2	0	4	S	2	1	1	Ru	2	0	0	H
C	4	12	30	F	1	0	0	Cl	1	0	1	Pd	2	0	0	
N	3	2	6	P	3	0	0	Br	1	0	0	I	3	0	0	

Error Margin (ppm): 5
HC Ratio: unlimited
Max Isotopes: 5
MSn Iso RI (%): 10.00

DBE Range: 10.0 - 20.0
Apply N Rule: yes
Isotope RI (%): 1.00
MSn Logic Mode: AND

Electron Ions: both
Use MSn Info: yes
Isotope Res: 9000
Max Results: 50

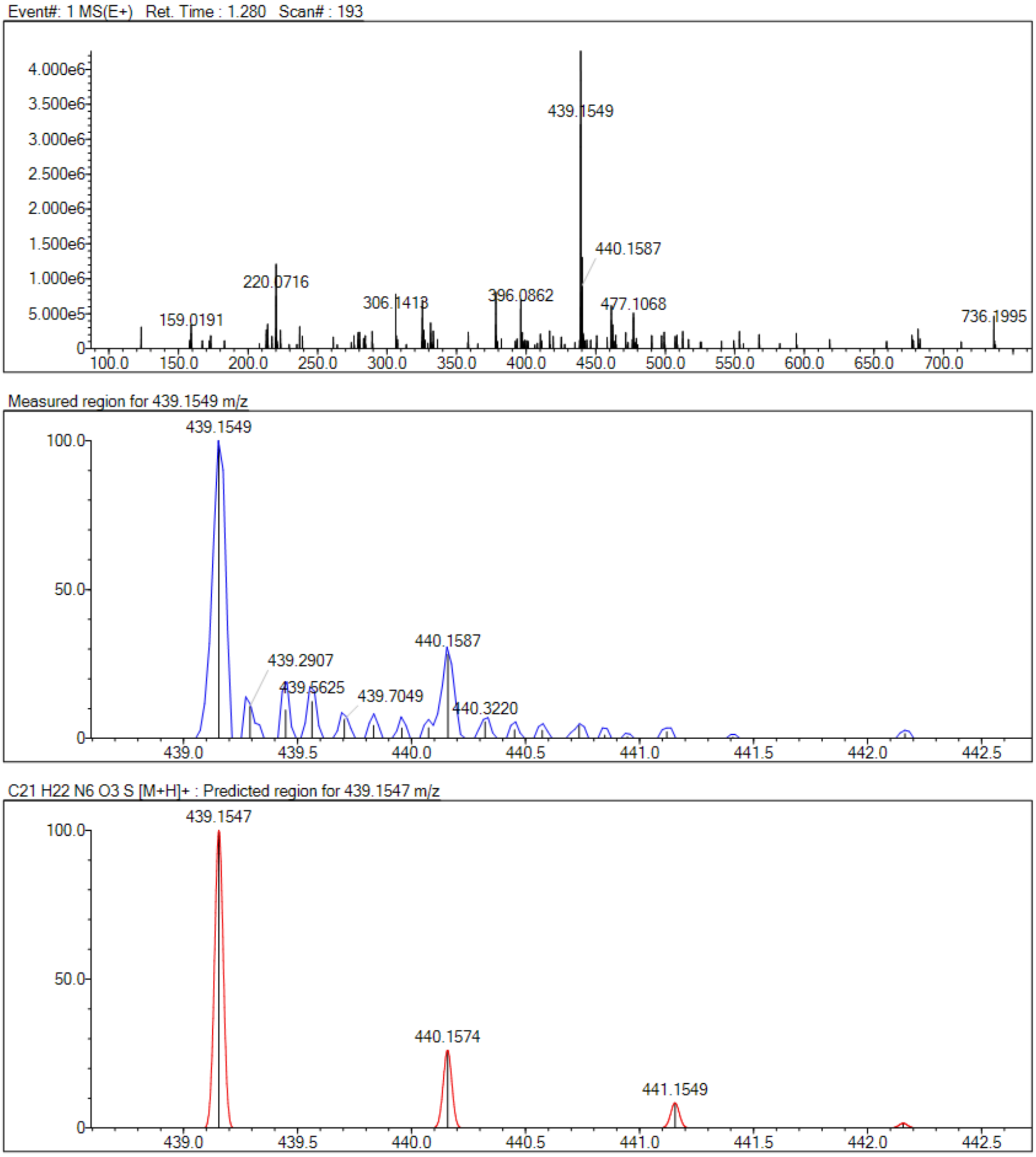

Rank	Score	Formula (M)	Ion	Meas. m/z	Pred. m/z	Df. (mDa)	Df. (ppm)	Iso	DBE
1	38.50	C21 H22 N6 O3 S	[M+H]+	439.1549	439.1547	0.2	0.46	38.50	14.0

Figure S12. Compound **5d** HRMS spectrum.

N-Cyclopropyl-2-((4-ethyl-5-(2-(4-hydroxyphenyl)-1H-benzo[d]imidazol-6-yl)-4H-1,2,4-triazol-3-yl)thio)acetamide ***(5e)***

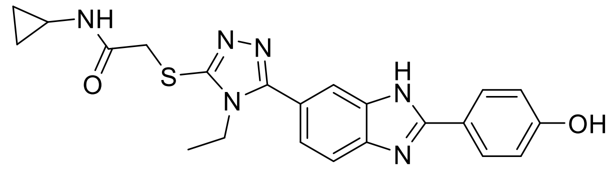

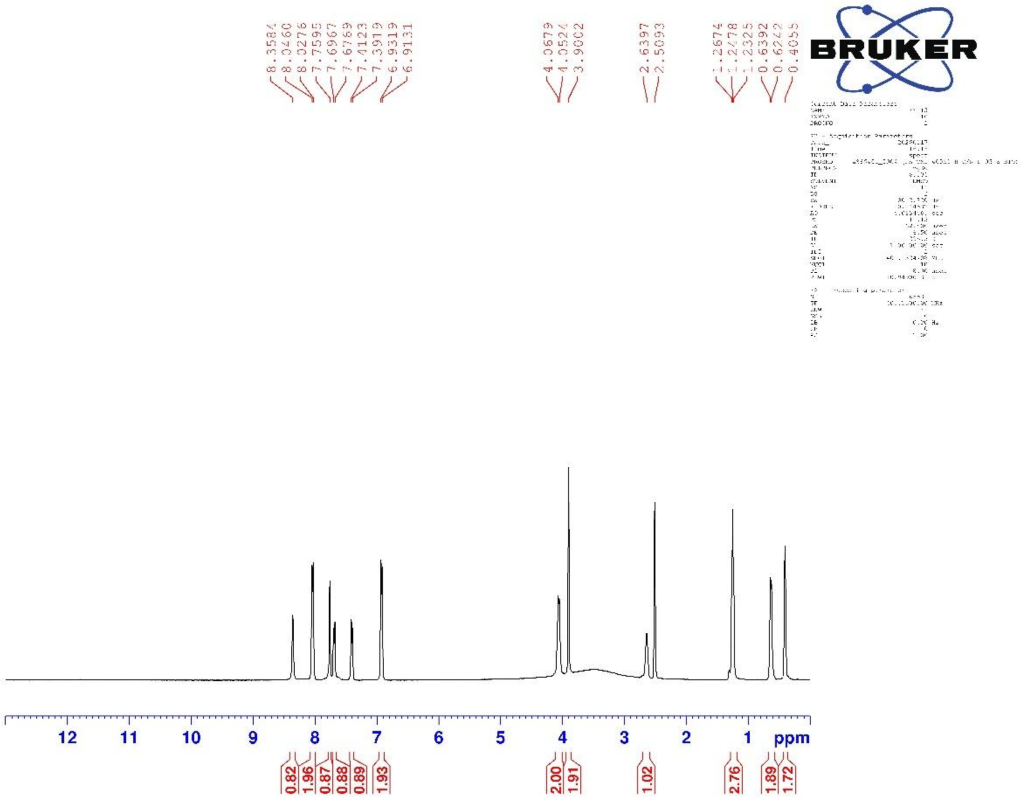

Figure S13. Compound **5e** ^{1}H-NMR spectrum.

N-Cyclopropyl-2-((4-ethyl-5-(2-(4-hydroxyphenyl)-1H-benzo[d]imidazol-6-yl)-4H-1,2,4-triazol-3-yl)thio)acetamide ***(5e)***

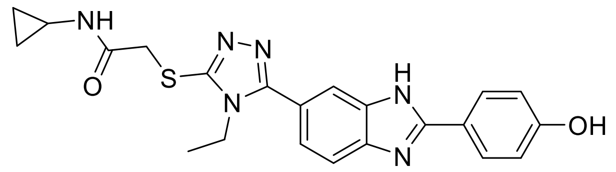

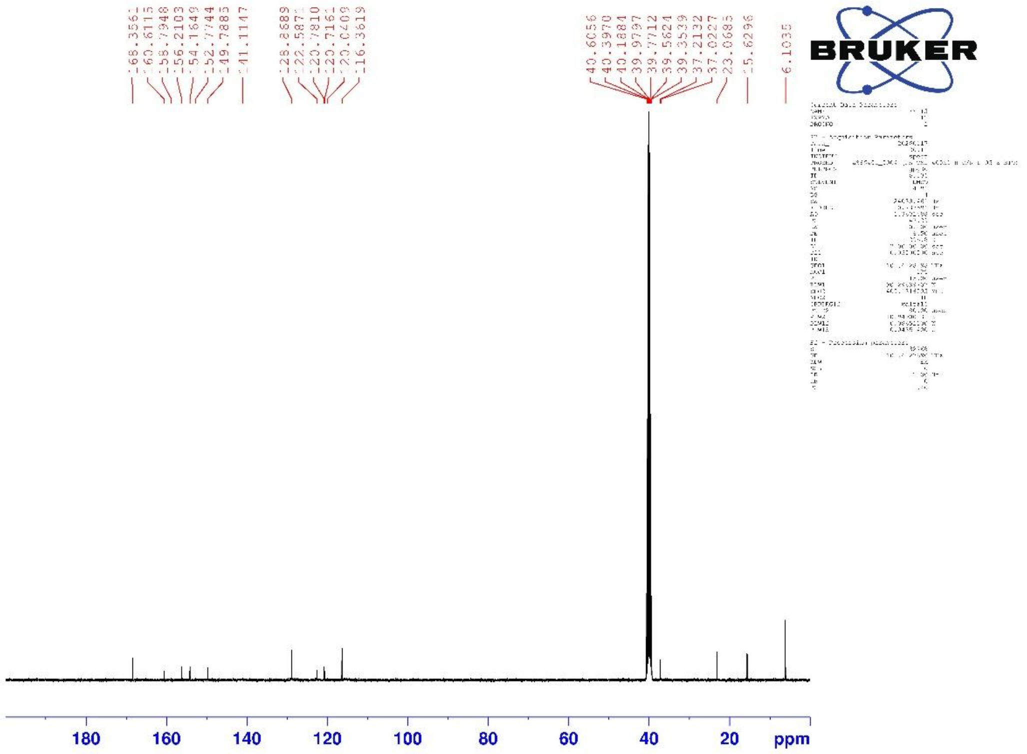

Figure S14. Compound **5e** ^{13}C-NMR spectrum.

N-Cyclopropyl-2-((4-ethyl-5-(2-(4-hydroxyphenyl)-1H-benzo[d]imidazol-6-yl)-4H-1,2,4-triazol-3-yl)thio)acetamide ***(5e)***

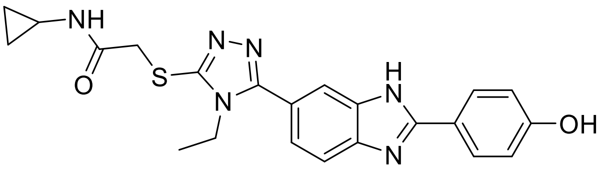

Elmt	Val.	Min	Max	Elmt	Val.	Min	Max	Elmt	Val.	Min	Max	Elmt	Val.	Min	Max	Use Adduct
H	1	10	30	O	2	0	4	S	2	1	1	Ru	2	0	0	H
C	4	12	30	F	1	0	0	Cl	1	0	1	Pd	2	0	0	
N	3	2	6	P	3	0	0	Br	1	0	0	I	3	0	0	

Error Margin (ppm):	5	DBE Range:	11.0 - 20.0	Electron Ions:	both
HC Ratio:	unlimited	Apply N Rule:	yes	Use MSn Info:	yes
Max Isotopes:	5	Isotope RI (%):	1.00	Isotope Res:	9000
MSn Iso RI (%):	10.00	MSn Logic Mode:	AND	Max Results:	50

Event#: 1 MS(E+) Ret. Time : 1.040 Scan# : 157

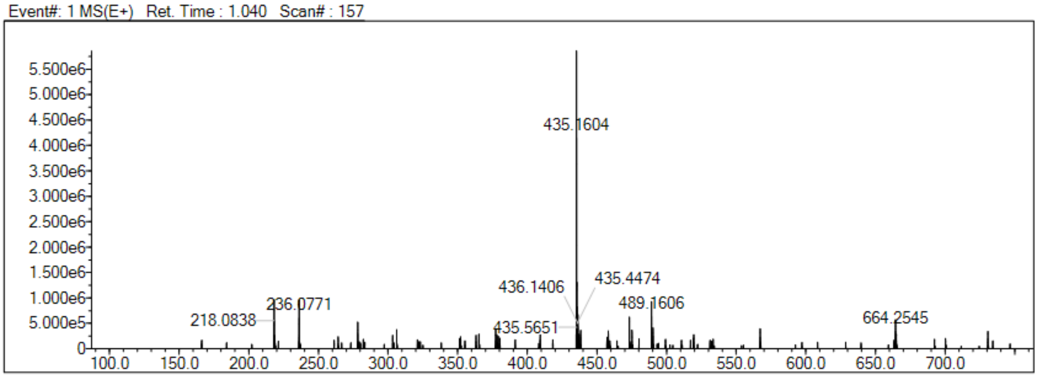

Measured region for 435.1604 m/z

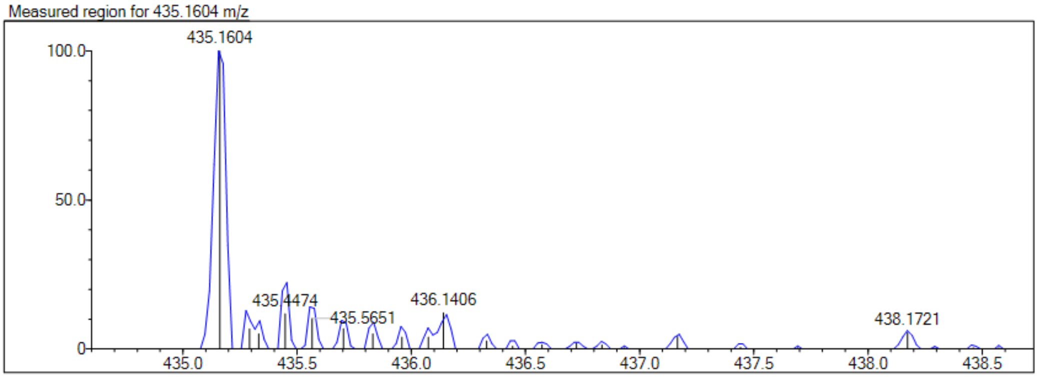

C22 H22 N6 O2 S [M+H]+ : Predicted region for 435.1598 m/z

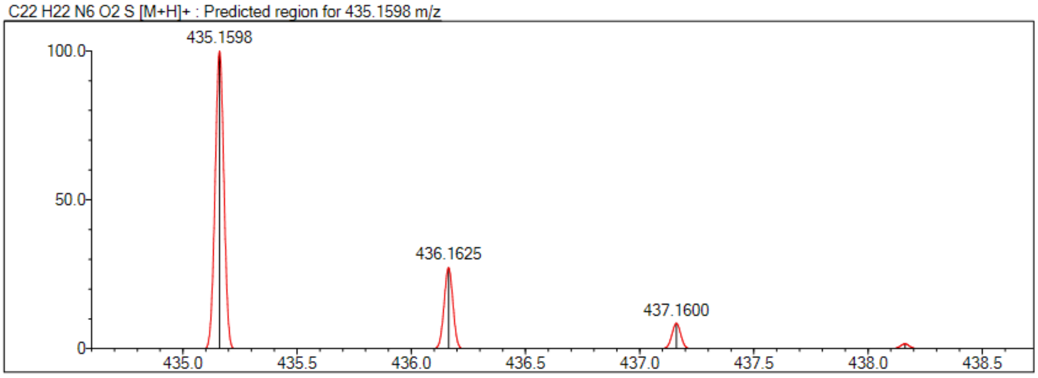

Rank	Score	Formula (M)	Ion	Meas. m/z	Pred. m/z	Df. (mDa)	Df. (ppm)	Iso	DBE
1	34.46	C22 H22 N6 O2 S	[M+H]+	435.1604	435.1598	0.6	1.38	34.79	15.0

Figure S15. Compound **5e** HRMS spectrum.

2-((4-Ethyl-5-(2-(4-hydroxyphenyl)-1H-benzo[d]imidazol-6-yl)-4H-1,2,4-triazol-3-yl)thio)-N,N-dimethylacetamide ***(5f)***

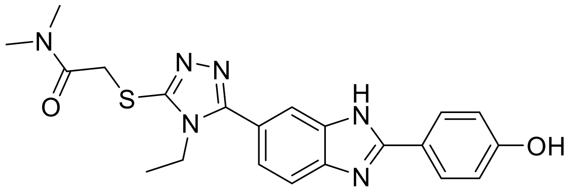

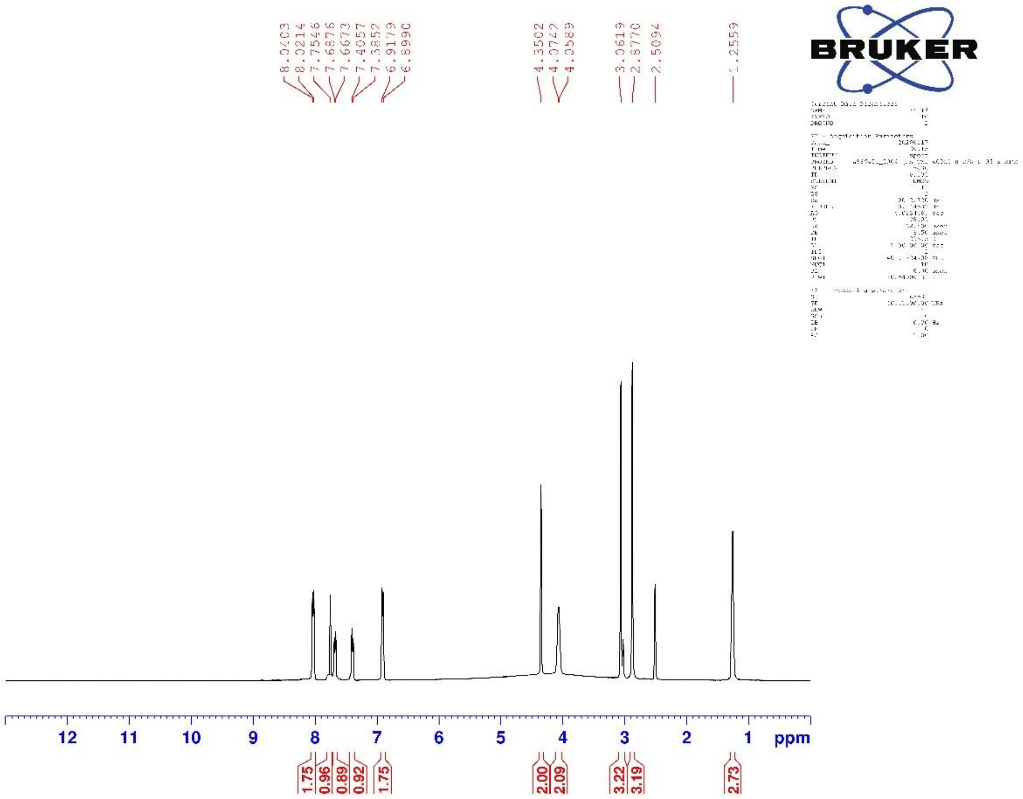

Figure S16. Compound **5f** ^{1}H-NMR spectrum.

2-((4-Ethyl-5-(2-(4-hydroxyphenyl)-1H-benzo[d]imidazol-6-yl)-4H-1,2,4-triazol-3-yl)thio)-N,N-dimethylacetamide ***(5f)***

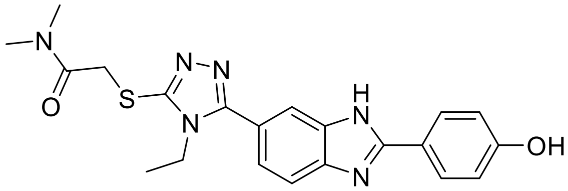

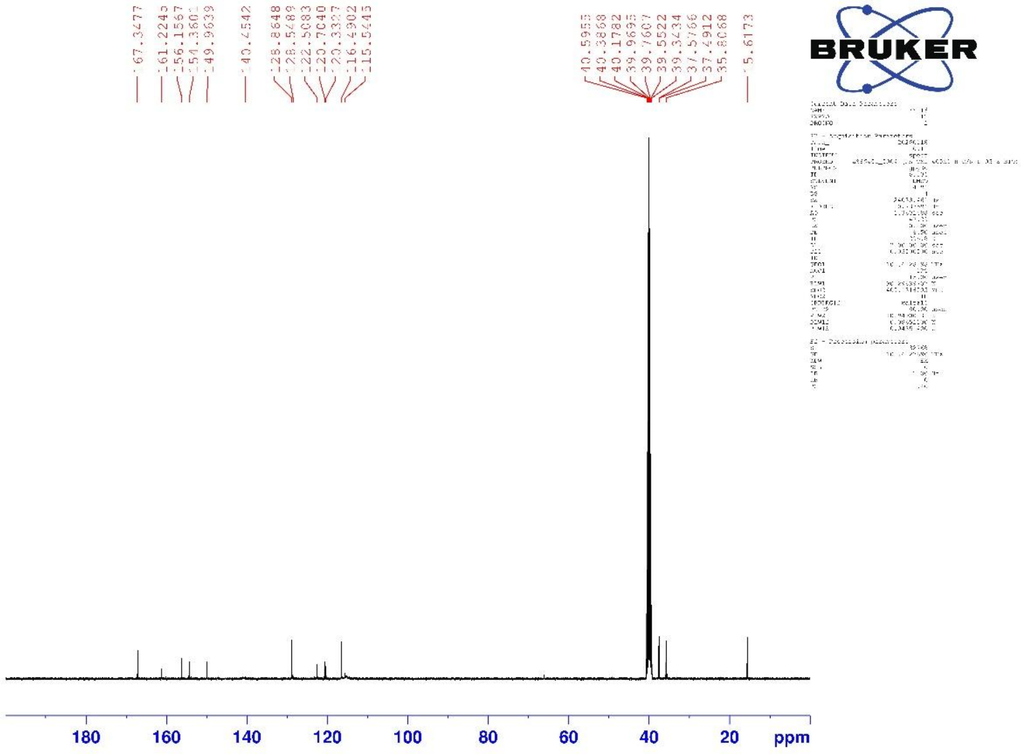

Figure S17. Compound **5f** ^{13}C-NMR spectrum.

2-((4-Ethyl-5-(2-(4-hydroxyphenyl)-1H-benzo[d]imidazol-6-yl)-4H-1,2,4-triazol-3-yl)thio)-N,N-dimethylacetamide **(5f)**

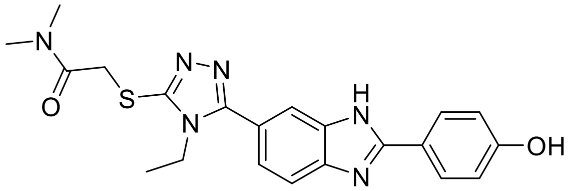

Elmt	Val.	Min	Max	Elmt	Val.	Min	Max	Elmt	Val.	Min	Max	Elmt	Val.	Min	Max	Use Adduct
H	1	10	30	O	2	0	4	S	2	1	1	Ru	2	0	0	H
C	4	12	30	F	1	0	0	Cl	1	0	1	Pd	2	0	0	
N	3	2	6	P	3	0	0	Br	1	0	0	I	3	0	0	

Error Margin (ppm):	9	DBE Range:	11.0 - 20.0	Electron Ions:	both
HC Ratio:	unlimited	Apply N Rule:	yes	Use MSn Info:	yes
Max Isotopes:	5	Isotope RI (%):	1.00	Isotope Res:	9000
MSn Iso RI (%):	10.00	MSn Logic Mode:	AND	Max Results:	50

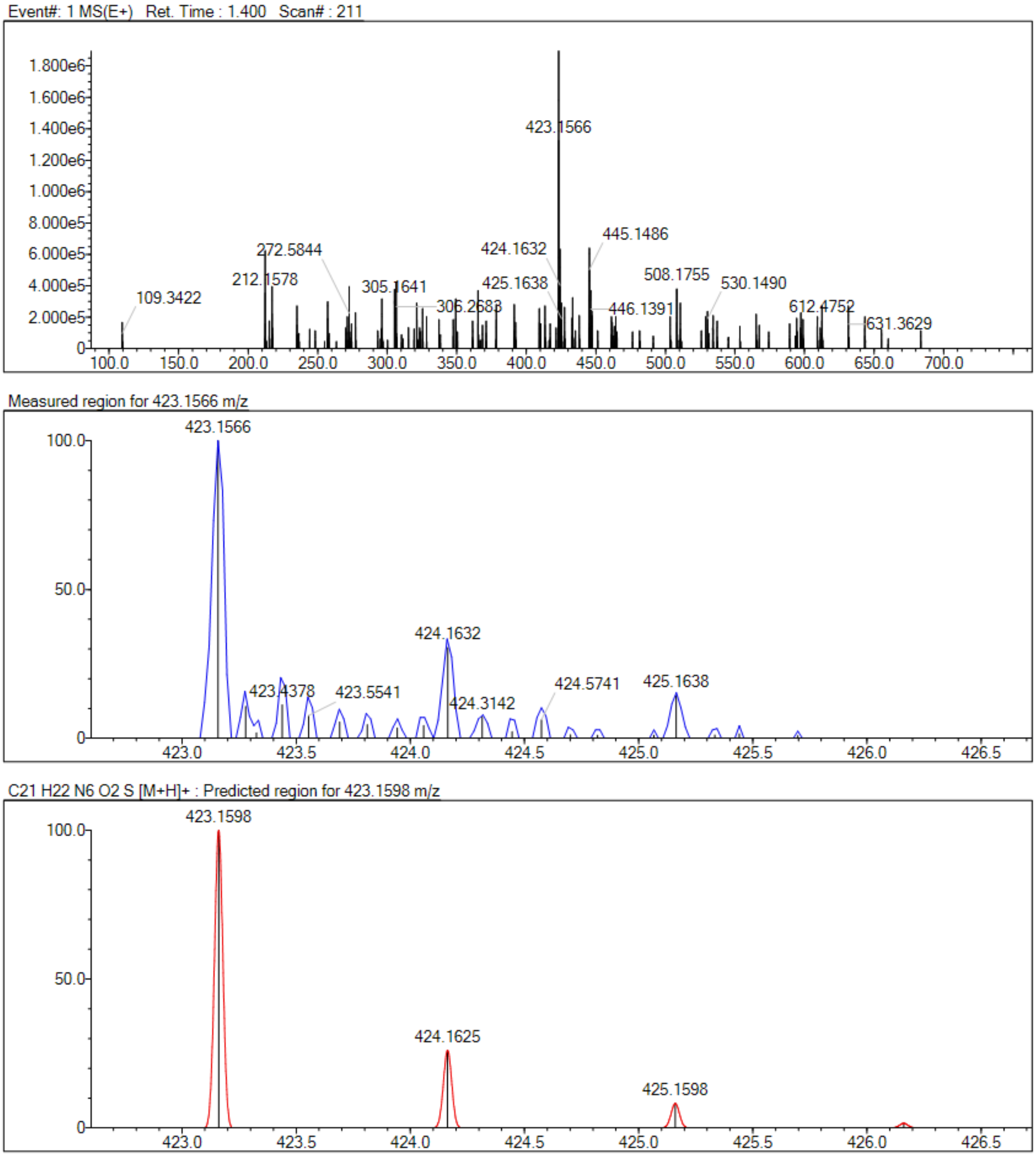

Rank	Score	Formula (M)	Ion	Meas. m/z	Pred. m/z	Df. (mDa)	Df. (ppm)	Iso	DBE
1	45.71	C21 H22 N6 O2 S	[M+H]+	423.1566	423.1598	-3.2	-7.56	70.97	14.0

Figure S18. Compound **5f** HRMS spectrum.

N,N-Diethyl-2-((4-ethyl-5-(2-(4-hydroxyphenyl)-1H-benzo[d]imidazol-6-yl)-4H-1,2,4-triazol-3-yl)thio)acetamide ***(5g)***

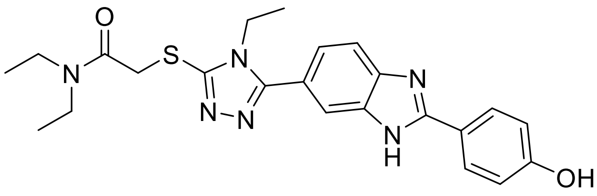

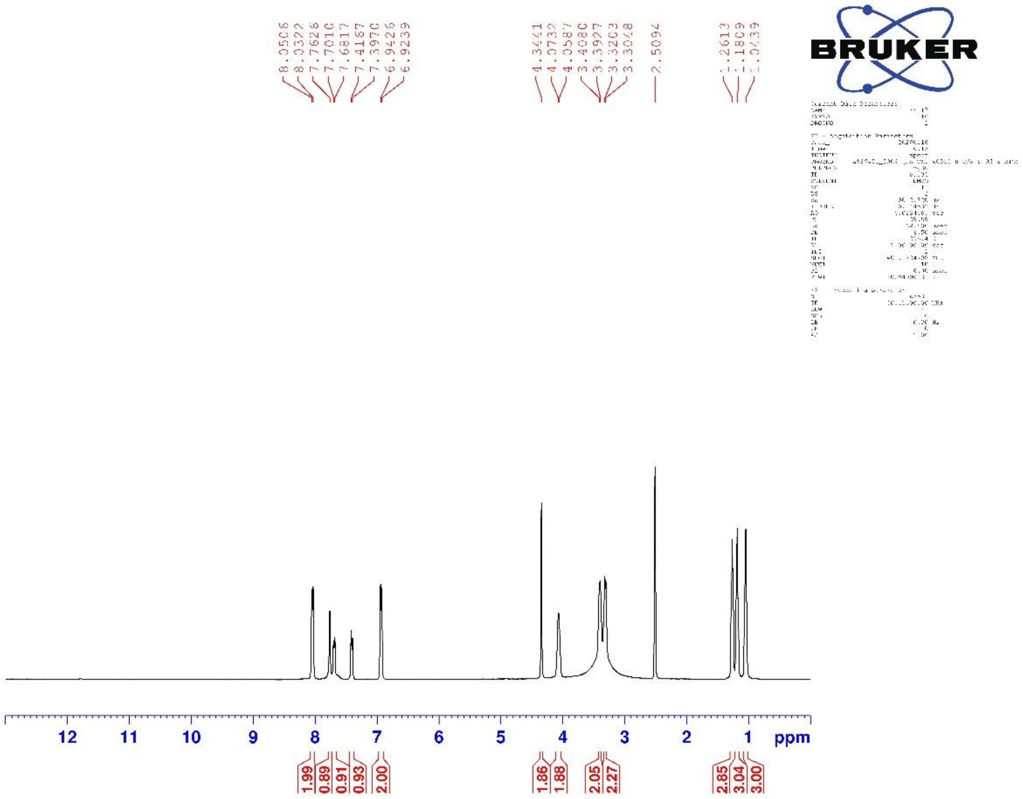

Figure S19. Compound **5g** ^{1}H-NMR spectrum.

N,N-Diethyl-2-((4-ethyl-5-(2-(4-hydroxyphenyl)-1H-benzo[d]imidazol-6-yl)-4H-1,2,4-triazol-3-yl)thio)acetamide ***(5g)***

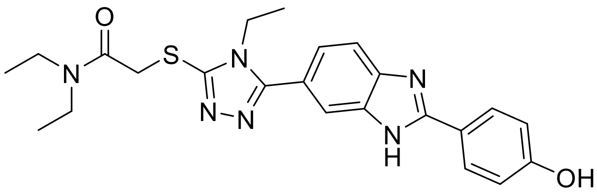

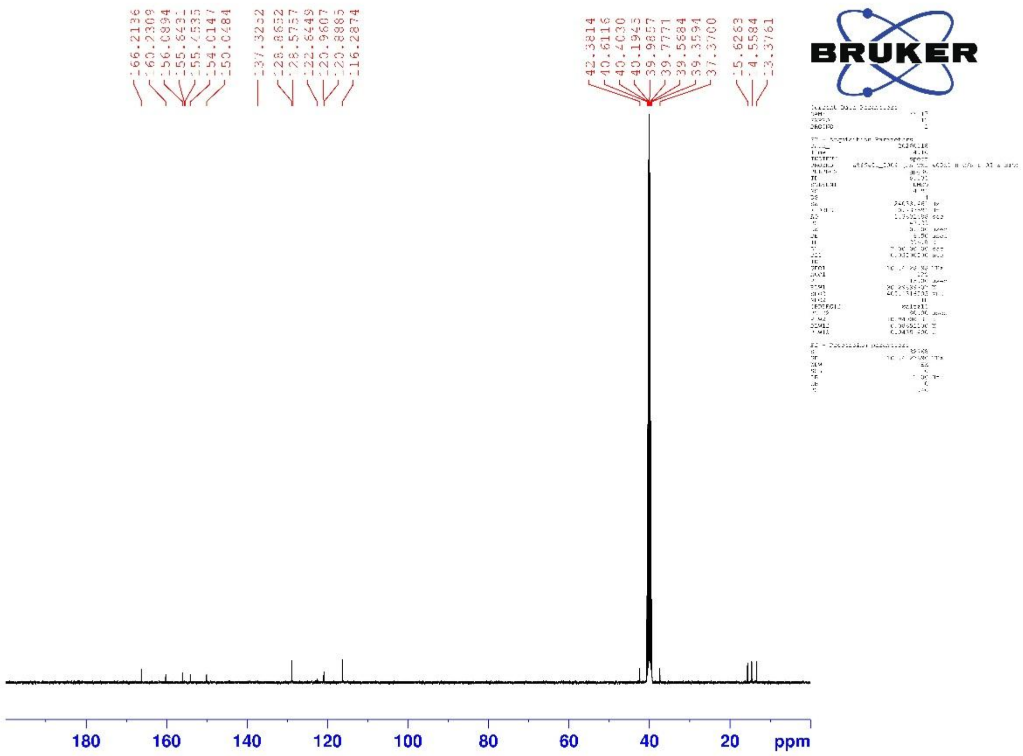

Figure S20. Compound **5g** ^{13}C-NMR spectrum.

N,N-Diethyl-2-((4-ethyl-5-(2-(4-hydroxyphenyl)-1H-benzo[d]imidazol-6-yl)-4H-1,2,4-triazol-3-yl)thio)acetamide ***(5g)***

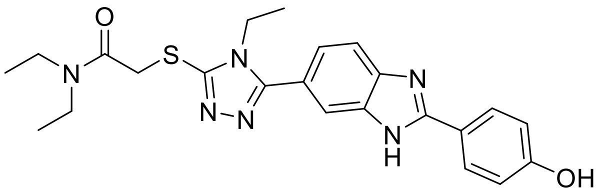

Elmt	Val.	Min	Max	Elmt	Val.	Min	Max	Elmt	Val.	Min	Max	Elmt	Val.	Min	Max	Use Adduct
H	1	10	30	O	2	0	4	S	2	1	1	Ru	2	0	0	H
C	4	12	30	F	1	0	0	Cl	1	0	1	Pd	2	0	0	
N	3	2	6	P	3	0	0	Br	1	0	0	I	3	0	0	

Error Margin (ppm): 15
HC Ratio: unlimited
Max Isotopes: 5
MSn Iso RI (%): 10.00

DBE Range: 11.0 - 17.0
Apply N Rule: yes
Isotope RI (%): 1.00
MSn Logic Mode: AND

Electron Ions: both
Use MSn Info: yes
Isotope Res: 9000
Max Results: 50

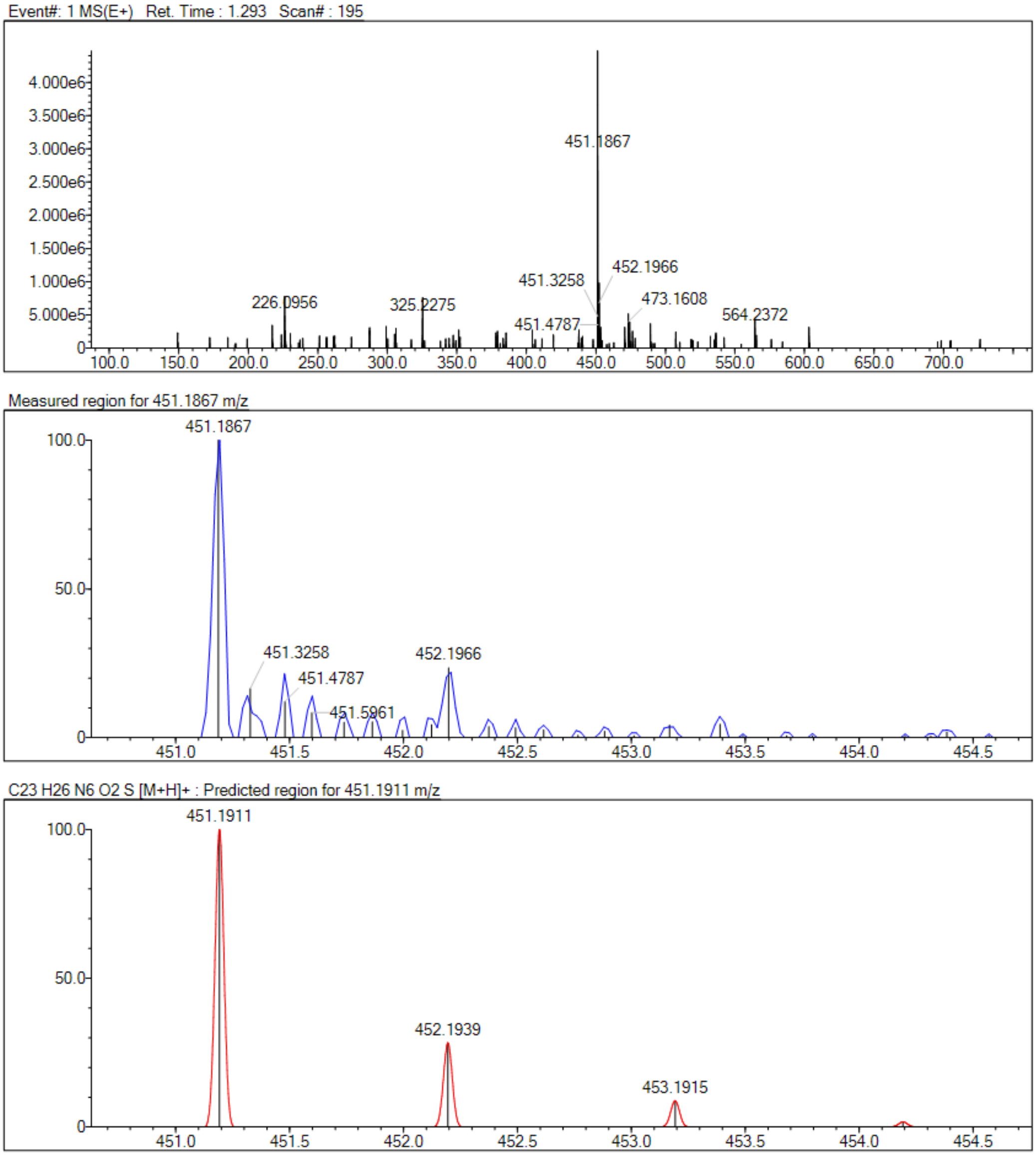

Rank	Score	Formula (M)	Ion	Meas. m/z	Pred. m/z	Df. (mDa)	Df. (ppm)	Iso	DBE
1	19.27	C23 H26 N6 O2 S	[M+H]+	451.1867	451.1911	-4.4	-9.75	45.34	14.0

Figure S21. Compound **5g** HRMS spectrum.

*2-((4-Ethyl-5-(2-(4-hydroxyphenyl)-1H-benzo[d]imidazol-6-yl)-4H-1,2,4-triazol-3-yl)thio)-1-morpholinoethan-1-one **(5h)***

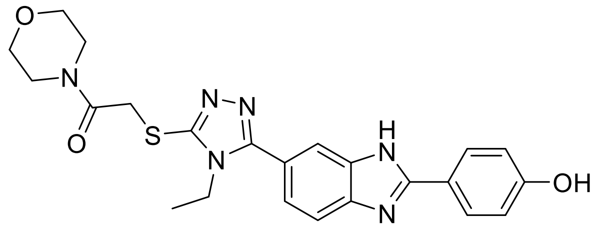

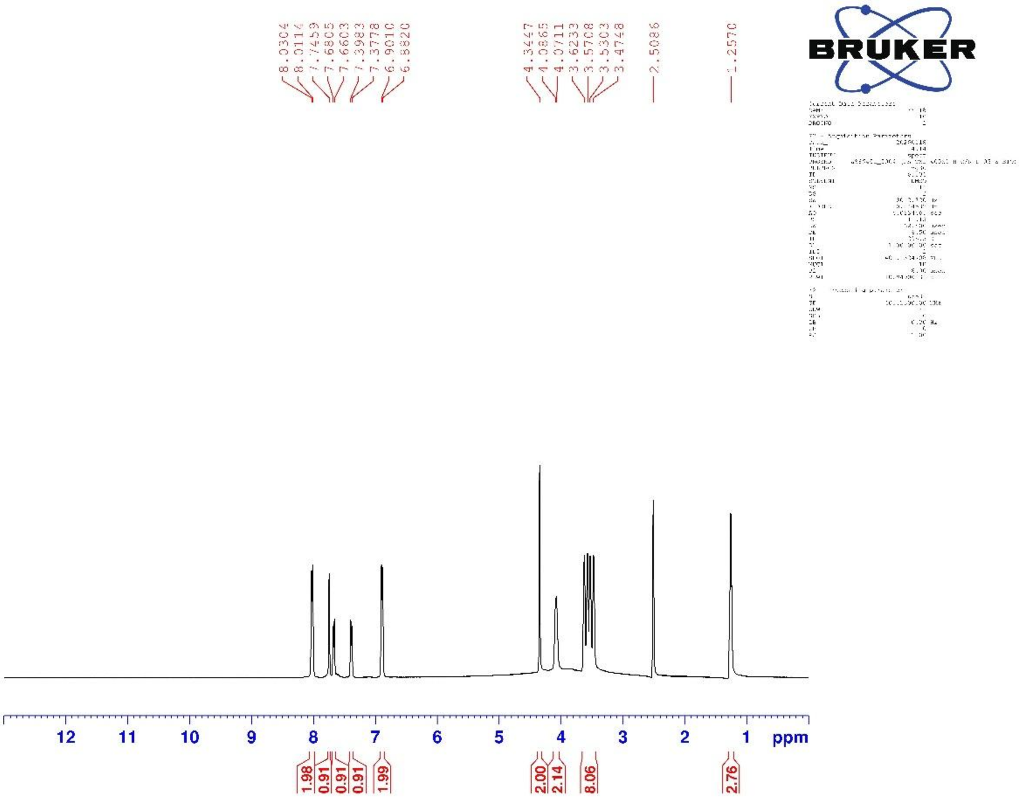

Figure S22. Compound **5h** ^{1}H-NMR spectrum.

2-((4-Ethyl-5-(2-(4-hydroxyphenyl)-1H-benzo[d]imidazol-6-yl)-4H-1,2,4-triazol-3-yl)thio)-1-morpholinoethan-1-one ***(5h)***

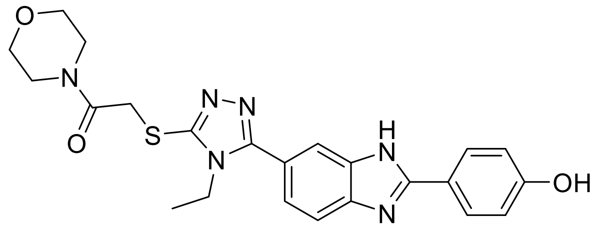

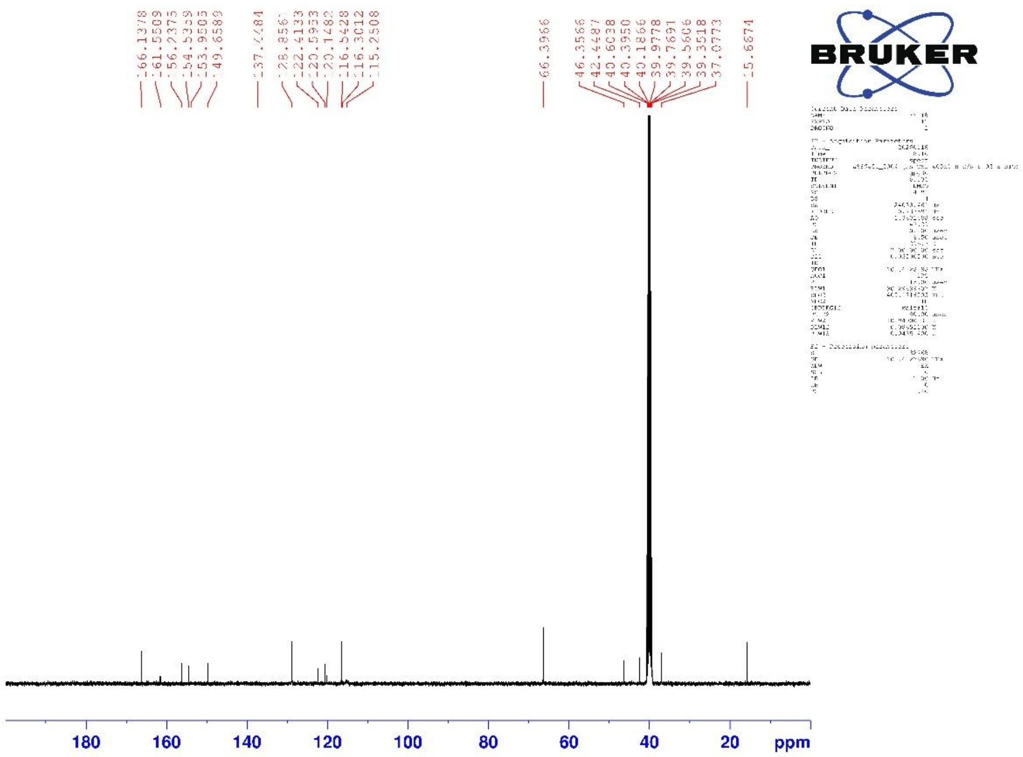

Figure S23. Compound **5h** ^{13}C-NMR spectrum.

2-((4-Ethyl-5-(2-(4-hydroxyphenyl)-1H-benzo[d]imidazol-6-yl)-4H-1,2,4-triazol-3-yl)thio)-1-morpholinoethan-1-one ***(5h)***

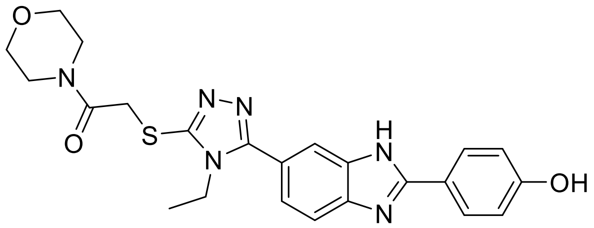

Elmt	Val.	Min	Max	Elmt	Val.	Min	Max	Elmt	Val.	Min	Max	Elmt	Val.	Min	Max	Use Adduct
H	1	10	30	O	2	1	4	S	2	1	1	Ru	2	0	0	H
C	4	12	30	F	1	0	0	Cl	1	0	1	Pd	2	0	0	
N	3	2	6	P	3	0	0	Br	1	0	0	I	3	0	0	

Error Margin (ppm):	15	DBE Range:	11.0 - 17.0	Electron Ions:	both
HC Ratio:	unlimited	Apply N Rule:	yes	Use MSn Info:	yes
Max Isotopes:	5	Isotope RI (%):	1.00	Isotope Res:	9000
MSn Iso RI (%):	10.00	MSn Logic Mode:	AND	Max Results:	50

Event#: 1 MS(E+) Ret. Time : 1.093 Scan# : 165

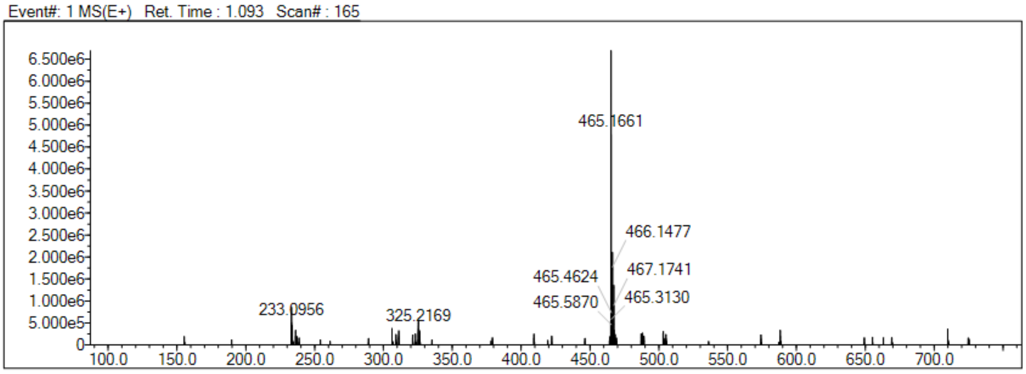

Measured region for 465.1661 m/z

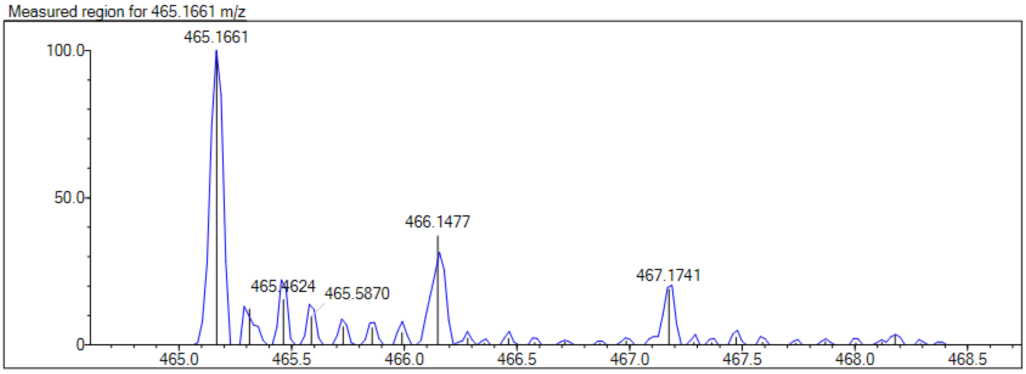

C23 H24 N6 O3 S [M+H]+ : Predicted region for 465.1703 m/z

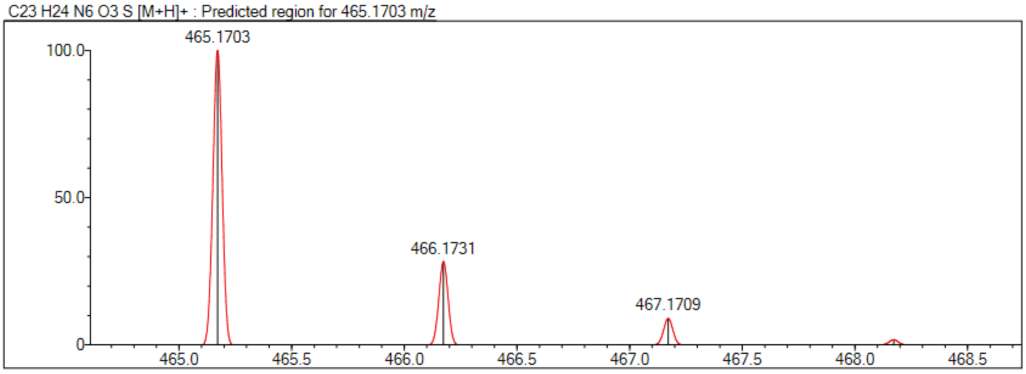

Rank	Score	Formula (M)	Ion	Meas. m/z	Pred. m/z	Df. (mDa)	Df. (ppm)	Iso	DBE
1	23.40	C23 H24 N6 O3 S	[M+H]+	465.1661	465.1703	-4.2	-9.03	47.07	15.0

Figure S24. Compound **5h** HRMS spectrum.

*2-((4-Ethyl-5-(2-(4-hydroxyphenyl)-1H-benzo[d]imidazol-6-yl)-4H-1,2,4-triazol-3-yl)thio)-N-phenylacetamide **(5i)***

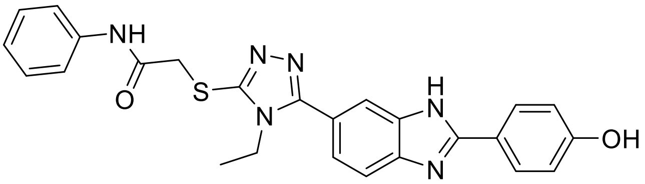

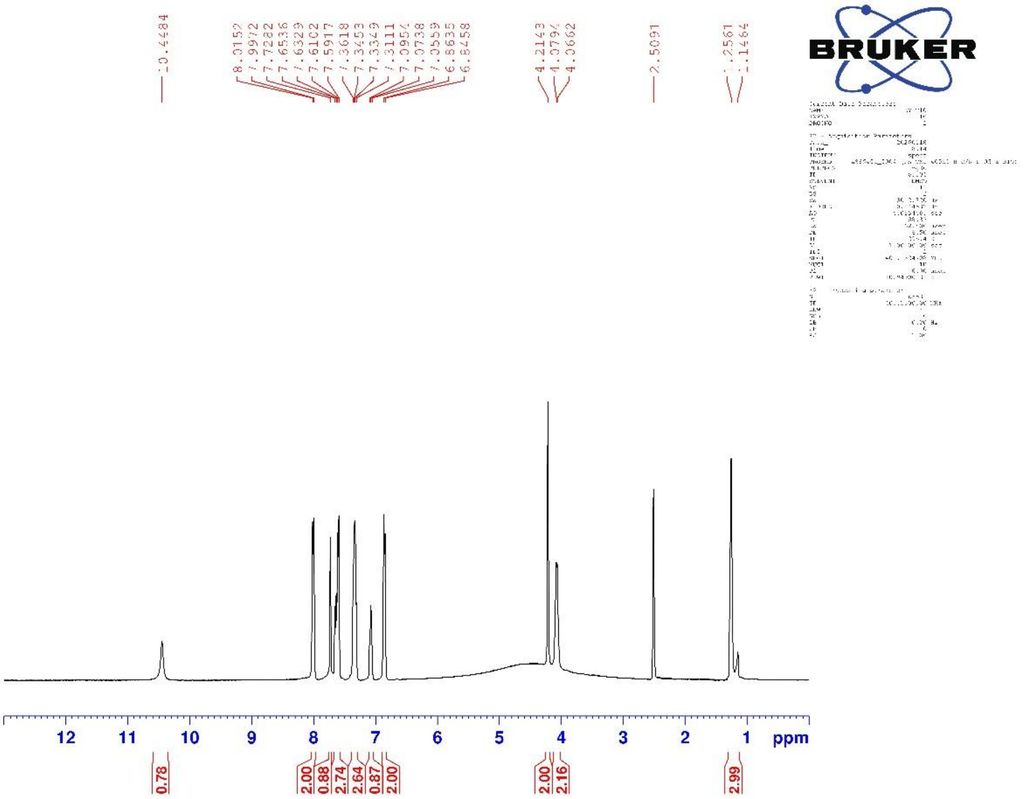

Figure S25. Compound **5i** ^{1}H-NMR spectrum.

2-((4-Ethyl-5-(2-(4-hydroxyphenyl)-1H-benzo[d]imidazol-6-yl)-4H-1,2,4-triazol-3-yl)thio)-N-phenylacetamide ***(5i)***

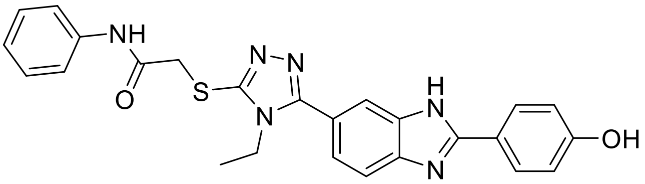

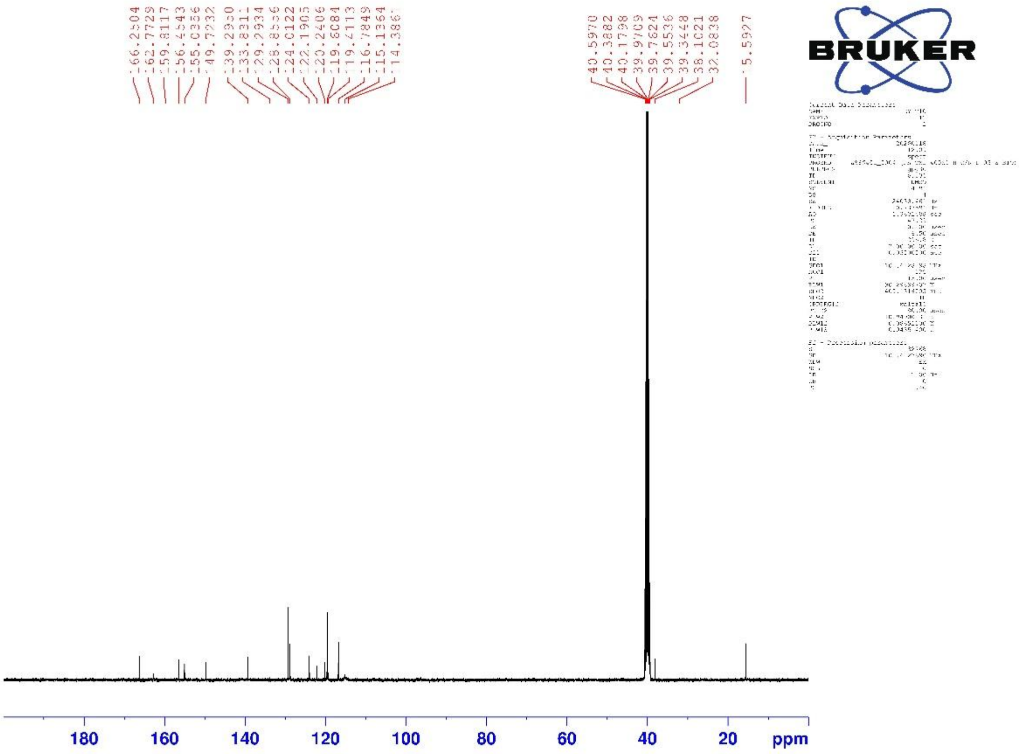

Figure S26. Compound **5i** ^{13}C-NMR spectrum.

2-((4-Ethyl-5-(2-(4-hydroxyphenyl)-1H-benzo[d]imidazol-6-yl)-4H-1,2,4-triazol-3-yl)thio)-N-phenylacetamide **(5i)**

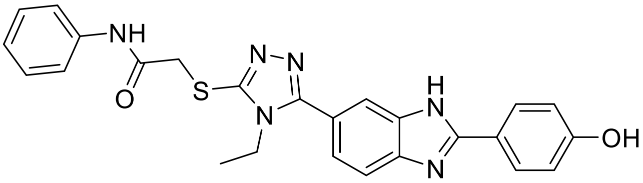

Elmt	Val.	Min	Max	Elmt	Val.	Min	Max	Elmt	Val.	Min	Max	Elmt	Val.	Min	Max	Use Adduct
H	1	10	30	O	2	1	4	S	2	1	1	Ru	2	0	0	H
C	4	12	30	F	1	0	0	Cl	1	0	1	Pd	2	0	0	
N	3	2	6	P	3	0	0	Br	1	0	0	I	3	0	0	

Error Margin (ppm):	11	DBE Range:	14.0 - 20.0	Electron Ions:	both
HC Ratio:	unlimited	Apply N Rule:	yes	Use MSn Info:	yes
Max Isotopes:	5	Isotope RI (%):	1.00	Isotope Res:	9000
MSn Iso RI (%):	10.00	MSn Logic Mode:	AND	Max Results:	50

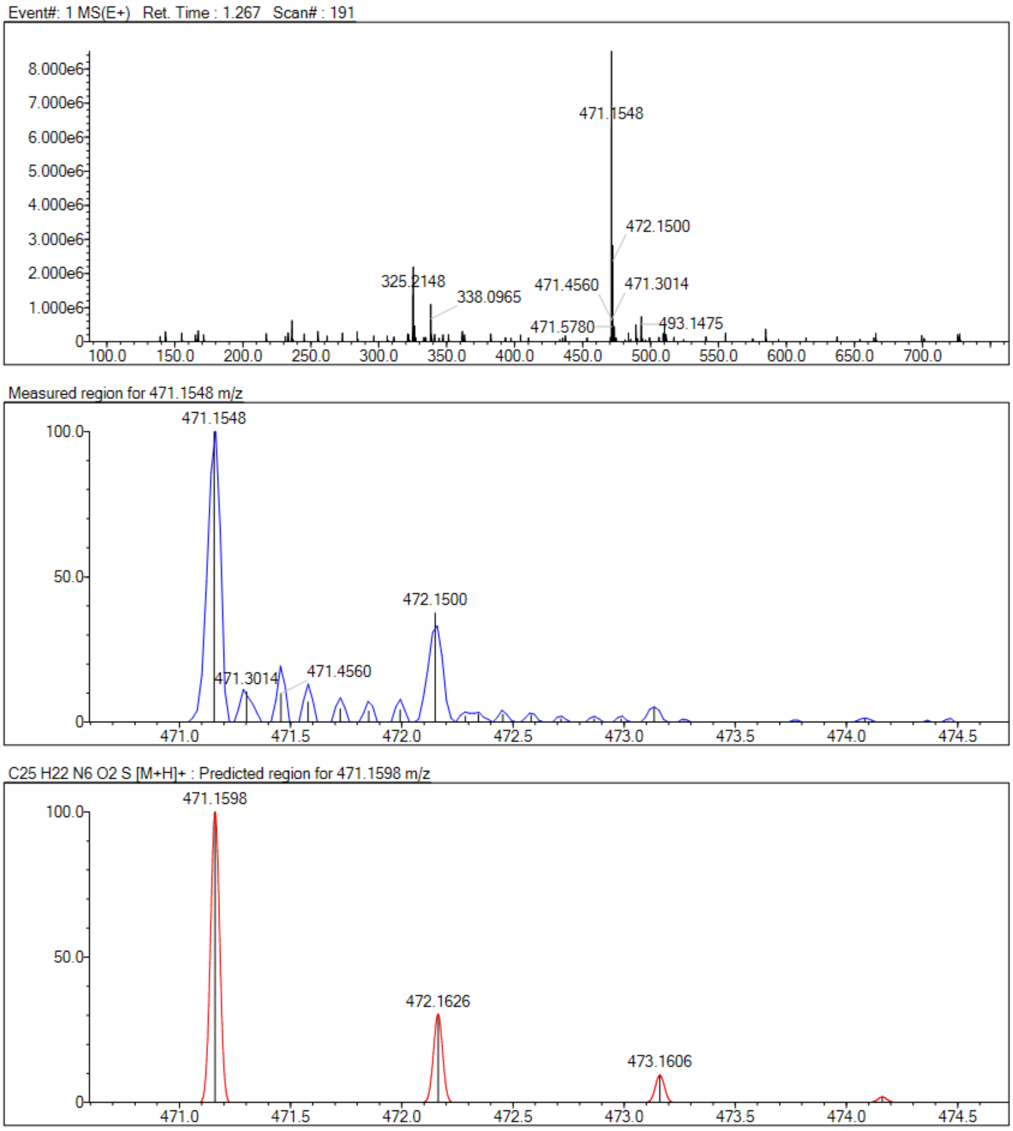

Rank	Score	Formula (M)	Ion	Meas. m/z	Pred. m/z	Df. (mDa)	Df. (ppm)	Iso	DBE
1	17.16	C25 H22 N6 O2 S	[M+H]+	471.1548	471.1598	-5.0	-10.61	44.73	18.0

Figure S27. Compound **5i** HRMS spectrum.

N-(4-chlorophenyl)-2-((4-ethyl-5-(2-(4-hydroxyphenyl)-1H-benzo[d]imidazol-6-yl)-4H-1,2,4-triazol-3-yl)thio)acetamide ***(5j)***

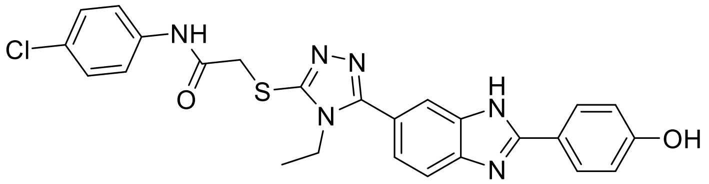

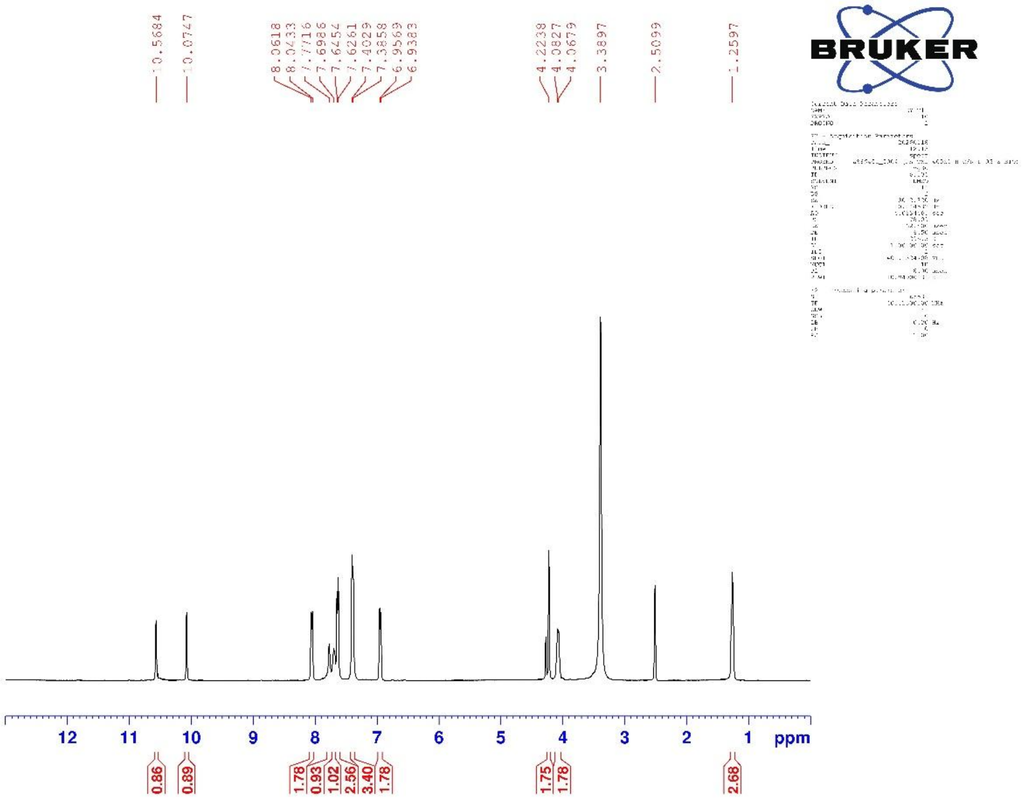

Figure S28. Compound **5j** ^{1}H-NMR spectrum.

N-(4-chlorophenyl)-2-((4-ethyl-5-(2-(4-hydroxyphenyl)-1H-benzo[d]imidazol-6-yl)-4H-1,2,4-triazol-3-yl)thio)acetamide ***(5j)***

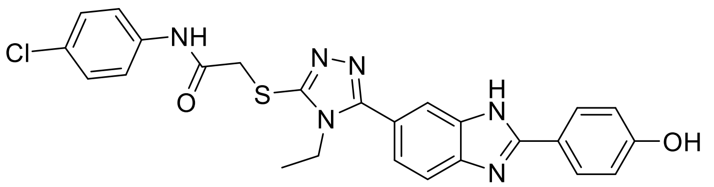

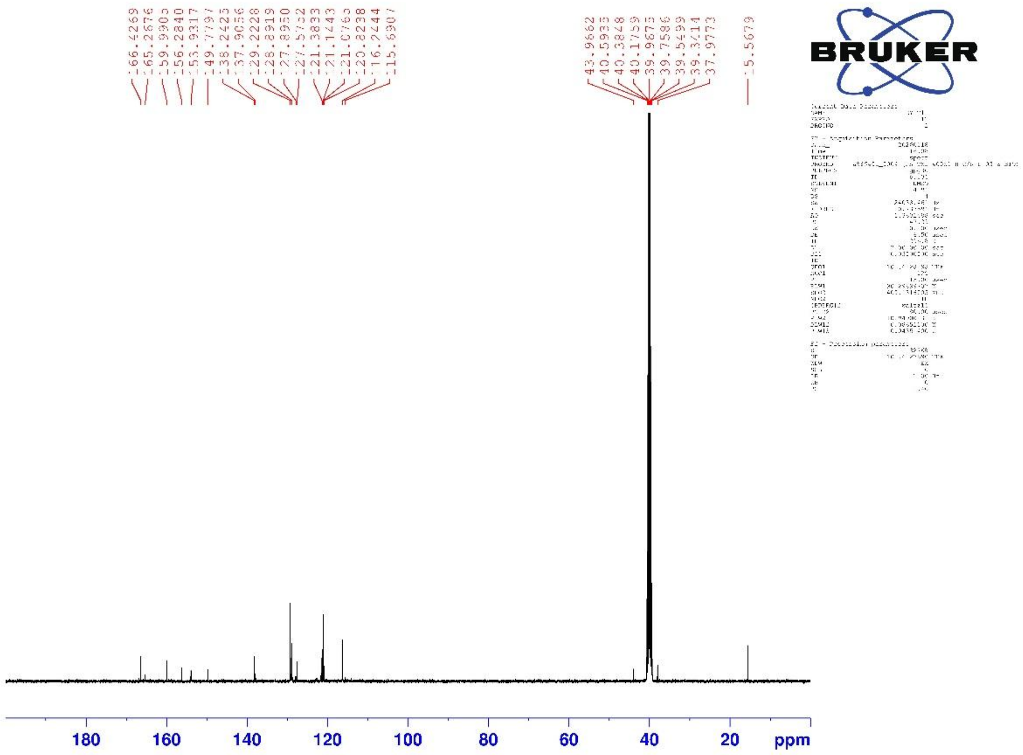

Figure S29. Compound **5j** ^{13}C-NMR spectrum.

N-(4-chlorophenyl)-2-((4-ethyl-5-(2-(4-hydroxyphenyl)-1H-benzo[d]imidazol-6-yl)-4H-1,2,4-triazol-3-yl)thio)acetamide ***(5j)***

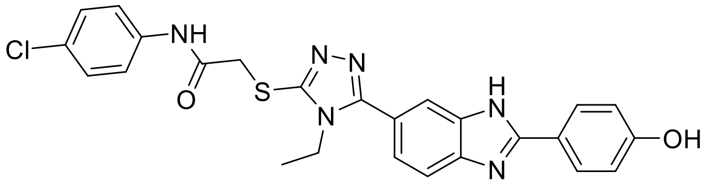

Elmt	Val.	Min	Max	Elmt	Val.	Min	Max	Elmt	Val.	Min	Max	Elmt	Val.	Min	Max	Use Adduct
H	1	10	30	O	2	1	4	S	2	1	1	Ru	2	0	0	H
C	4	12	30	F	1	0	0	Cl	1	0	1	Pd	2	0	0	
N	3	2	6	P	3	0	0	Br	1	0	0	I	3	0	0	

Error Margin (ppm): 11
HC Ratio: unlimited
Max Isotopes: 5
MSn Iso RI (%): 10.00

DBE Range: 14.0 - 20.0
Apply N Rule: yes
Isotope RI (%): 1.00
MSn Logic Mode: AND

Electron Ions: both
Use MSn Info: yes
Isotope Res: 9000
Max Results: 50

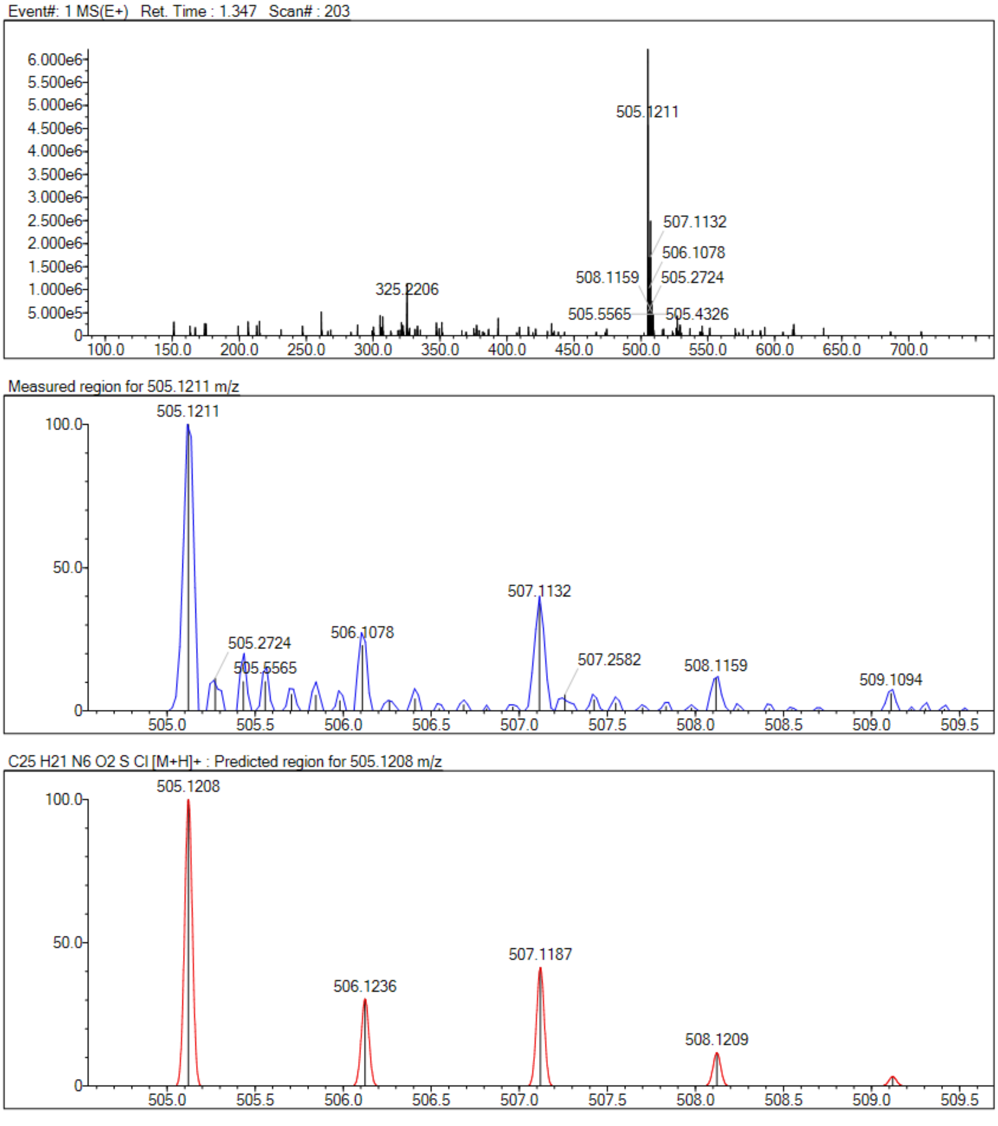

Rank	Score	Formula (M)	Ion	Meas. m/z	Pred. m/z	Df. (mDa)	Df. (ppm)	Iso	DBE
1	56.88	C25 H21 N6 O2 S Cl	[M+H]+	505.1211	505.1208	0.3	0.59	56.88	18.0

Figure S30. Compound **5j** HRMS spectrum.

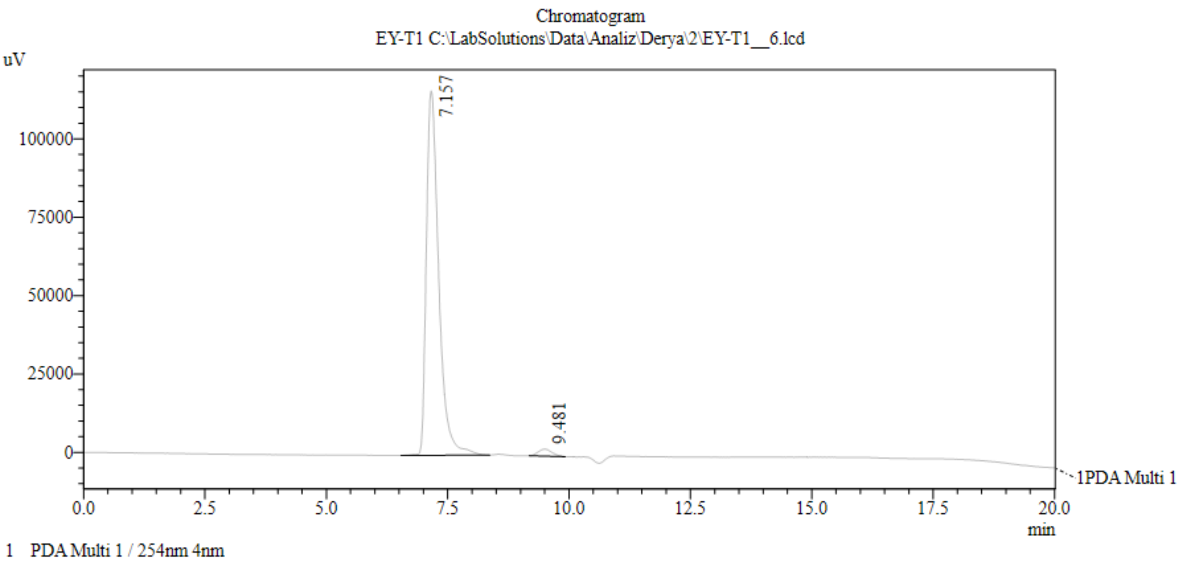

PeakTable C:\LabSolutions\Data\Analiz\Derya\2\EY-T1__6.lcd

PDA Ch1 254nm 4nm

Peak#	Ret. Time	Area	Height	Area %	Height %
1	7.157	2059859	116135	97.896	98.122
2	9.481	44279	2223	2.104	1.878
Total		2104138	118358	100.000	100.000

Figure S31. Compound **5a** HPLC chromatogram.

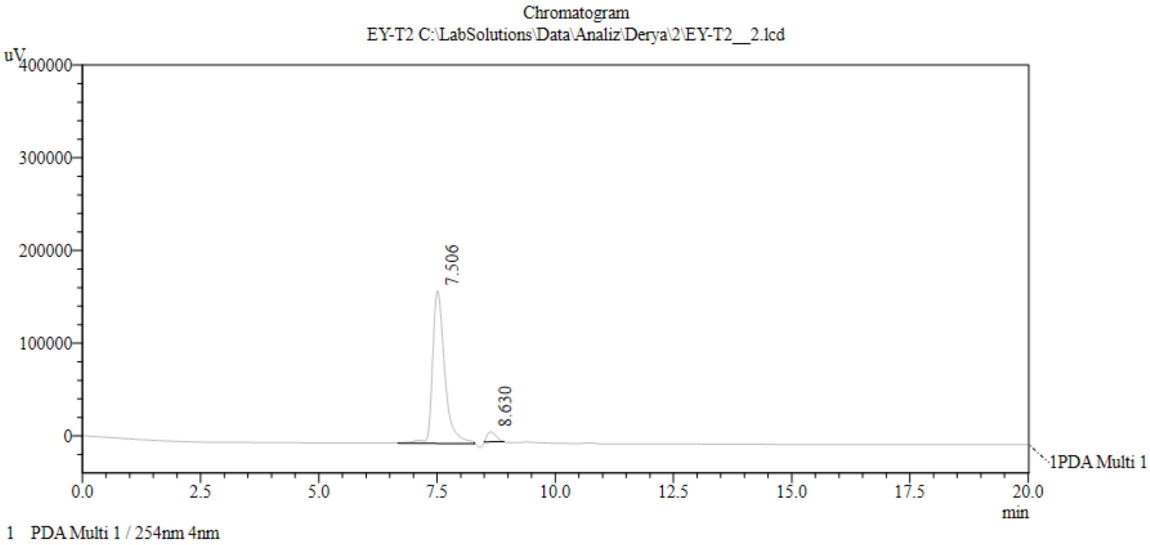

PeakTable C:\LabSolutions\Data\Analiz\Derya\2\EY-T2__2.lcd

PDA Ch1 254nm 4nm

Peak#	Ret. Time	Area	Height	Area %	USP Width
1	7.506	2951297	164874	95.460	0.445
2	8.630	140351	10572	4.540	0.361
Total		3091648	175446	100.000	

Figure S32. Compound **5b** HPLC chromatogram.

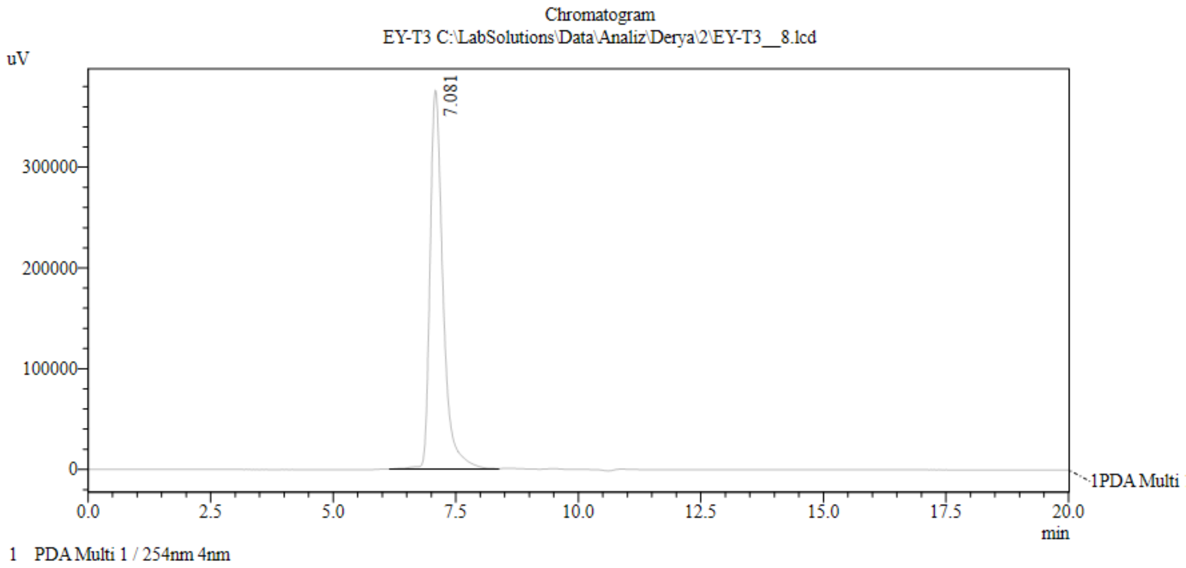

PeakTable C:\LabSolutions\Data\Analiz\Derya\2\EY-T3__8.lcd

PDA Ch1 254nm 4nm

Peak#	Ret. Time	Area	Height	Area %	Height %
1	7.081	6928873	375856	100.000	100.000
Total		6928873	375856	100.000	100.000

Figure S33. Compound **5c** HPLC chromatogram.

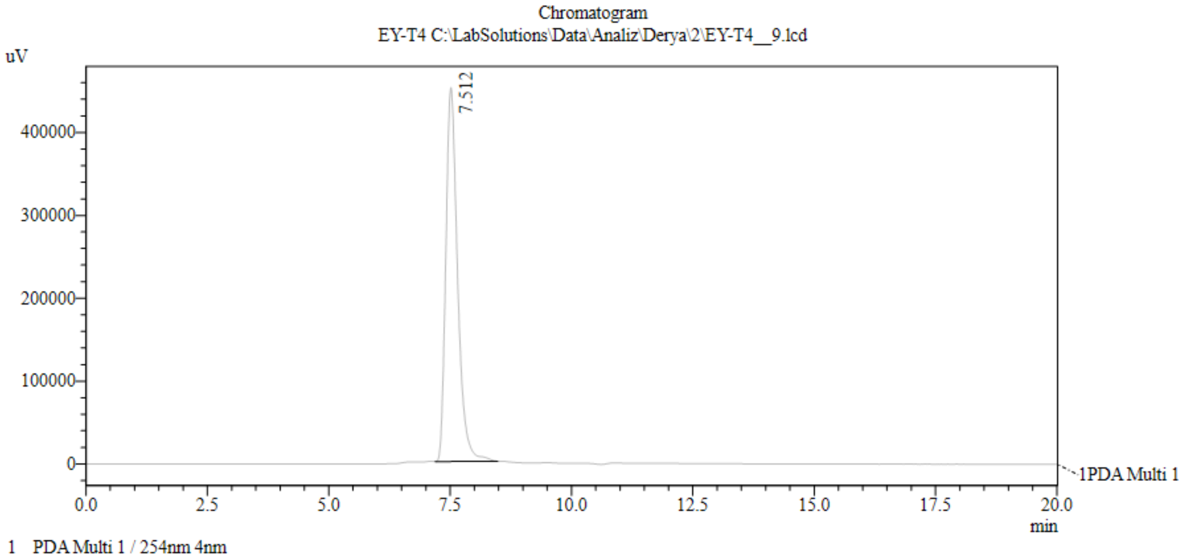

PeakTable C:\LabSolutions\Data\Analiz\Derya\2\EY-T4__9.lcd

PDA Ch1 254nm 4nm

Peak#	Ret. Time	Area	Height	Area %	Height %
1	7.512	7606580	451022	100.000	100.000
Total		7606580	451022	100.000	100.000

Figure S34. Compound **5d** HPLC chromatogram.

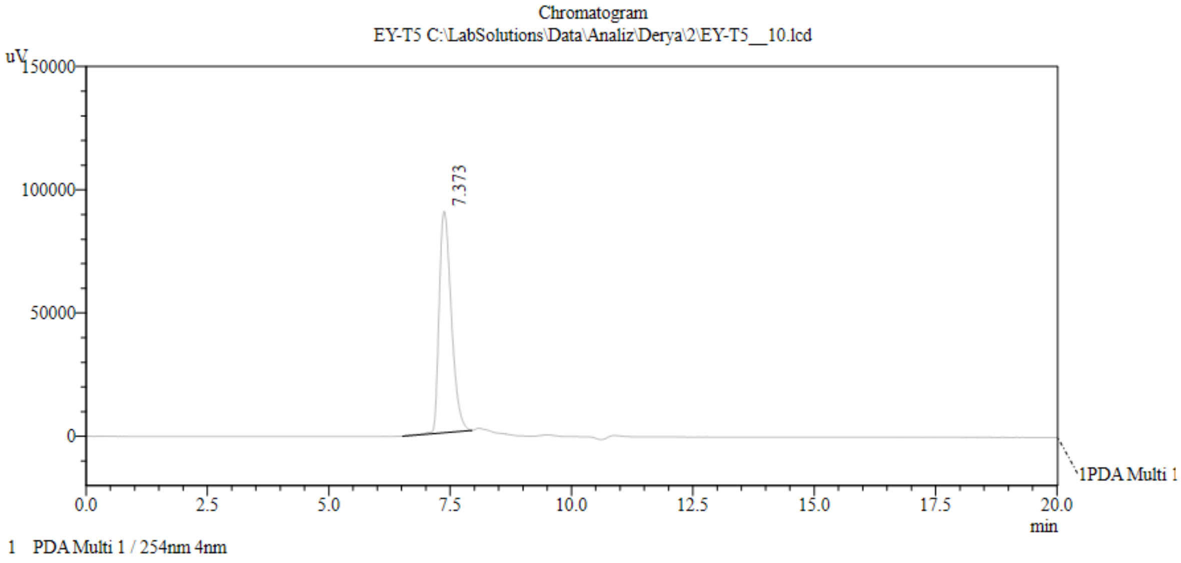

PeakTable C:\LabSolutions\Data\Analiz\Derya\2\EY-T5__10.lcd

PDA Ch1 254nm 4nm

Peak#	Ret. Time	Area	Height	Area %	Height %
1	7.373	1569686	89897	100.000	100.000
Total		1569686	89897	100.000	100.000

Figure S35. Compound **5e** HPLC chromatogram.

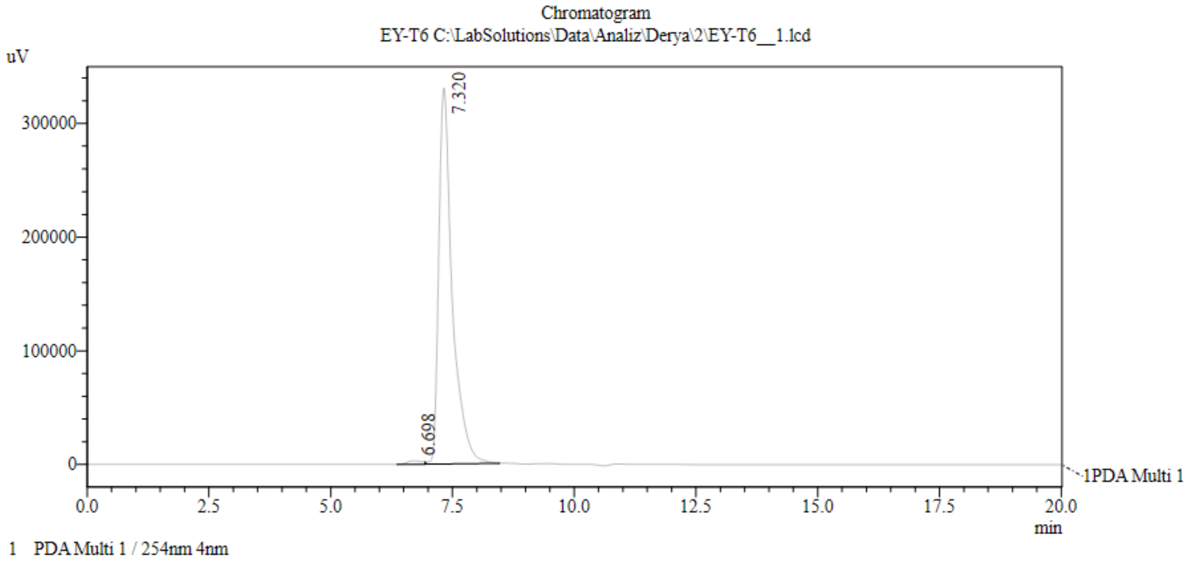

PeakTable C:\LabSolutions\Data\Analiz\Derya\2\EY-T6__1.lcd

PDA Ch1 254nm 4nm

Peak#	Ret. Time	Area	Height	Area %	Height %
1	6.698	63456	2995	0.987	0.898
2	7.320	6362494	330590	99.013	99.102
Total		6425950	333585	100.000	100.000

Figure S36. Compound **5f** HPLC chromatogram.

Chromatogram
EY-T7 C:\LabSolutions\Data\Analiz\Derya\2\EY-T7__2.lcd

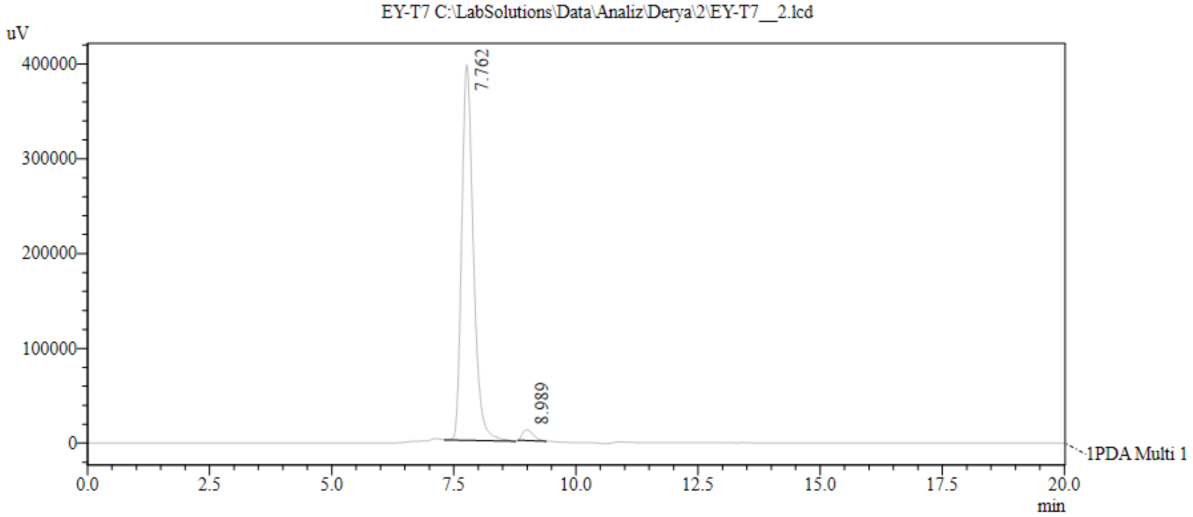

1 PDA Multi 1 / 254nm 4nm

PeakTable C:\LabSolutions\Data\Analiz\Derya\2\EY-T7__2.lcd

PDA Ch1 254nm 4nm

Peak#	Ret. Time	Area	Height	Area %	Height %
1	7.762	6598709	396214	97.391	97.216
2	8.989	176768	11348	2.609	2.784
Total		6775477	407561	100.000	100.000

Figure S37. Compound **5g** HPLC chromatogram.

Chromatogram
EY-T8 C:\LabSolutions\Data\Analiz\Derya\2\EY-T8__3.lcd

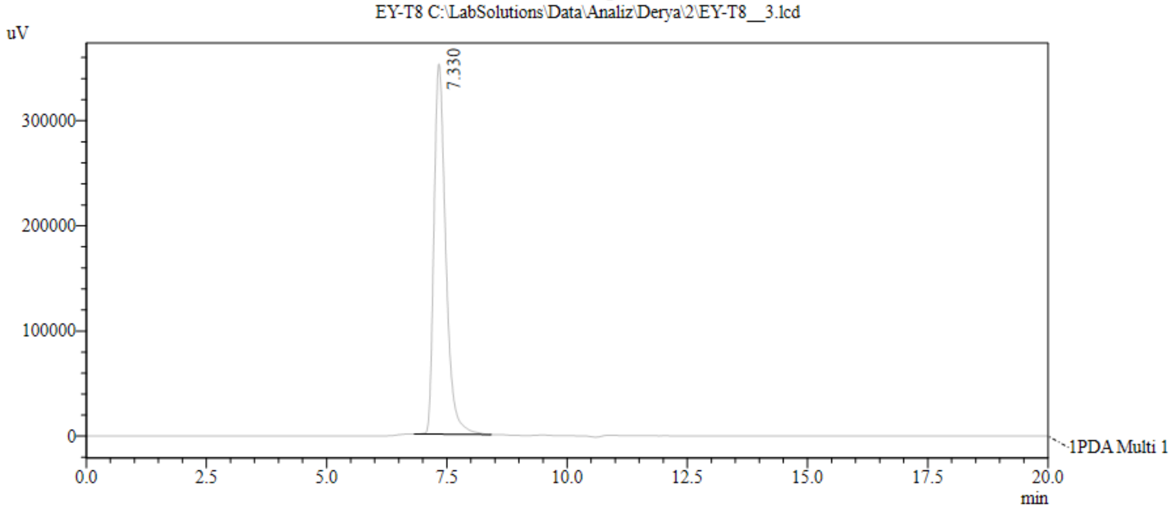

1 PDA Multi 1 / 254nm 4nm

PeakTable C:\LabSolutions\Data\Analiz\Derya\2\EY-T8__3.lcd

PDA Ch1 254nm 4nm

Peak#	Ret. Time	Area	Height	Area %	Height %
1	7.330	6002523	352045	100.000	100.000
Total		6002523	352045	100.000	100.000

Figure S38. Compound **5h** HPLC chromatogram.

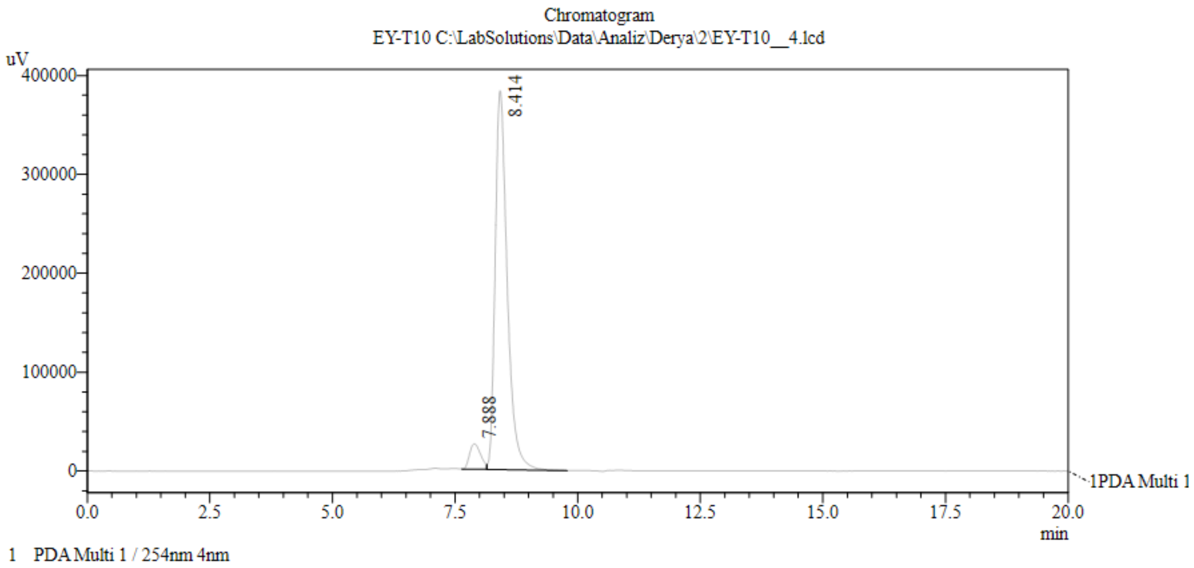

PeakTable C:\LabSolutions\Data\Analiz\Derya\2\EY-T10__4.lcd

PDA Ch1 254nm 4nm

Peak#	Ret. Time	Area	Height	Area %	Height %
1	7.888	409123	25805	5.870	6.312
2	8.414	6561163	383036	94.130	93.688
Total		6970287	408842	100.000	100.000

Figure S39. Compound **5i** HPLC chromatogram.

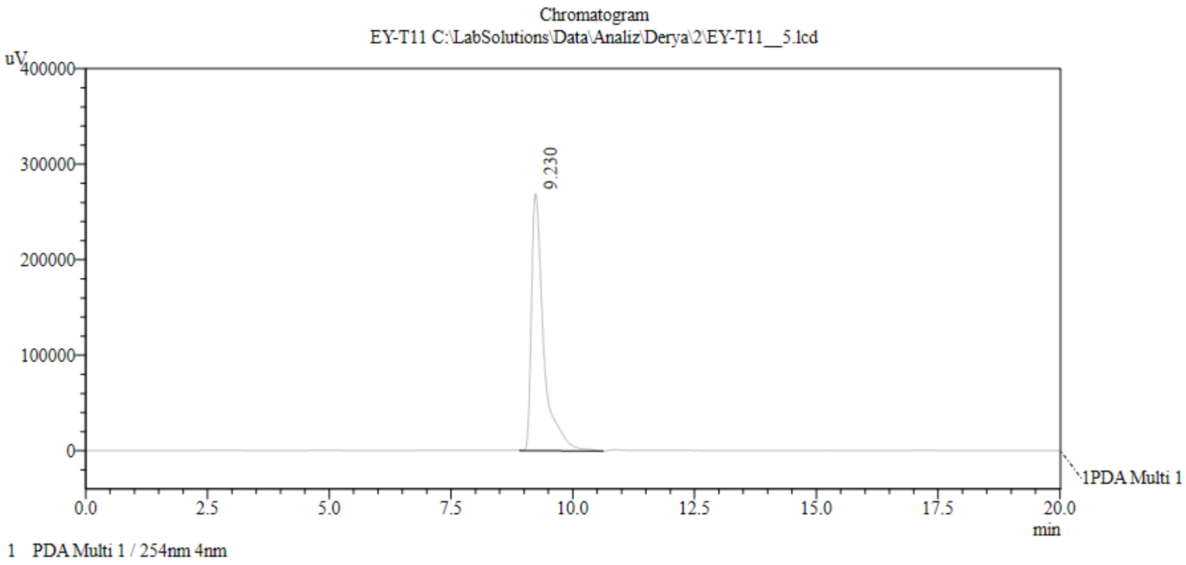

PeakTable C:\LabSolutions\Data\Analiz\Derya\2\EY-T11__5.lcd

PDA Ch1 254nm 4nm

Peak#	Ret. Time	Area	Height	Area %	Height %
1	9.230	4708164	269347	100.000	100.000
Total		4708164	269347	100.000	100.000

Figure S40. Compound **5j** HPLC chromatogram.

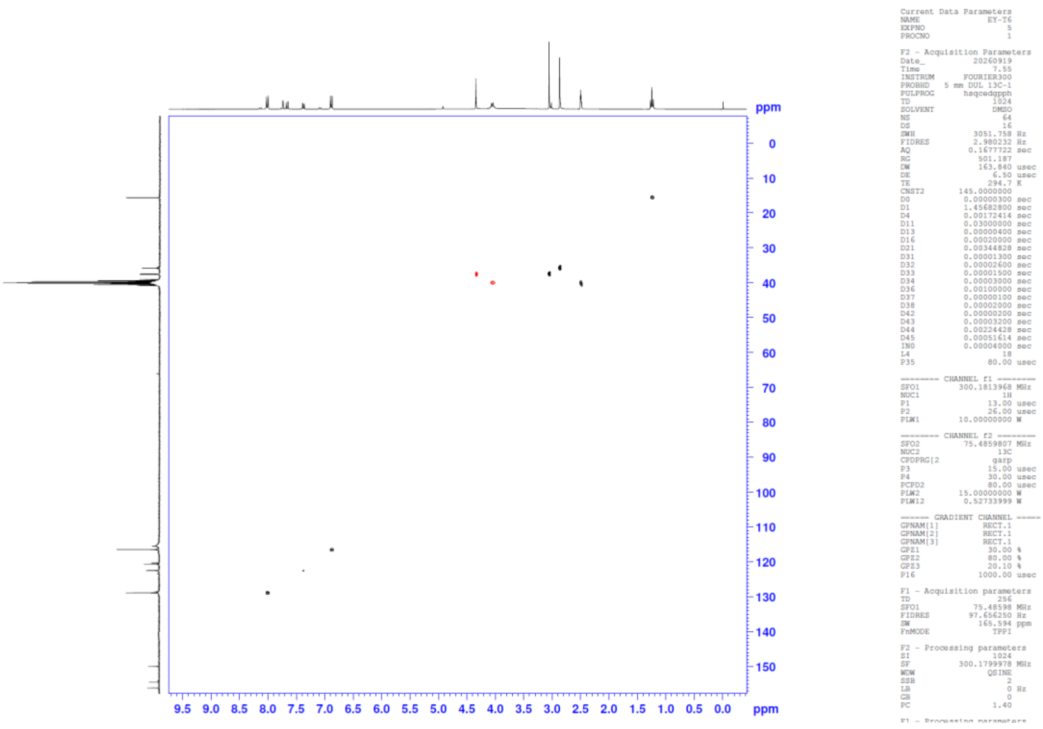

Figure S41. Compound **5f** HMQC spectrum.

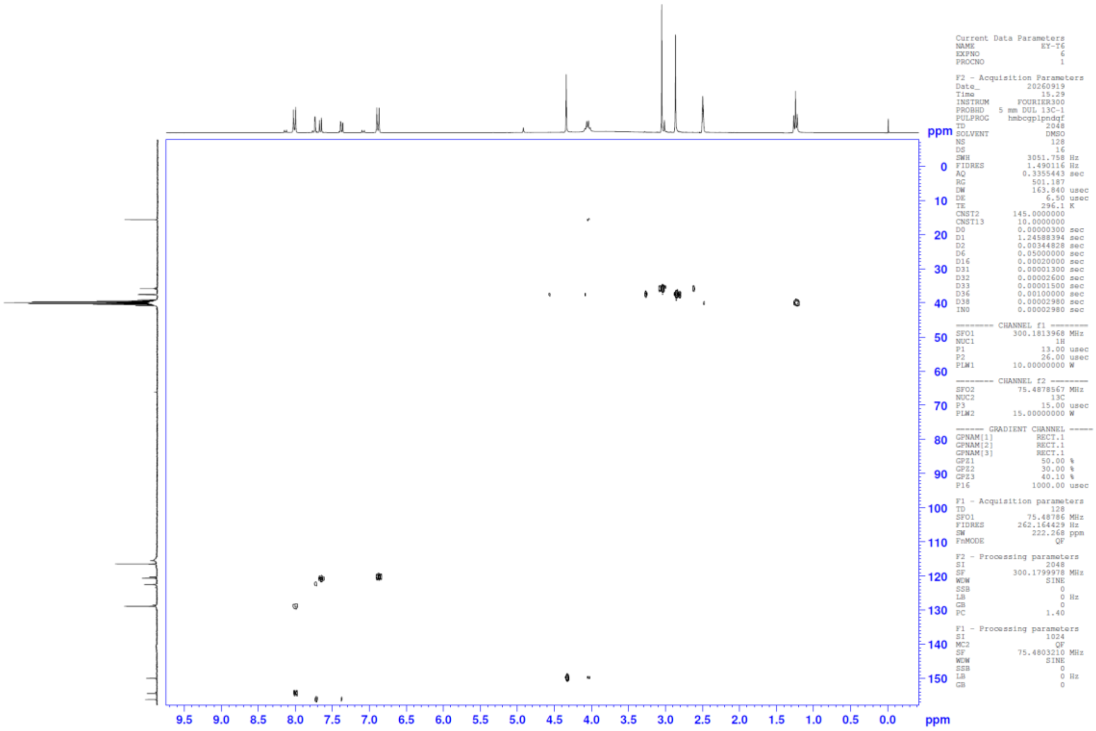

Figure S42. Compound **5f** HMBC spectrum.

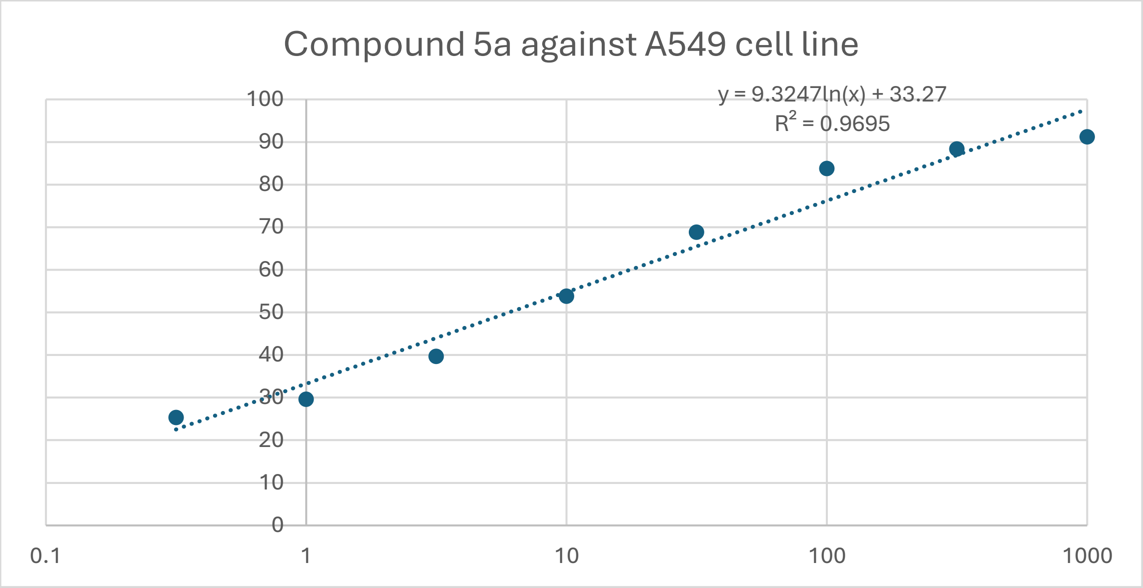

Figure S43. Dose–response curve of compound **5a** in A549 cells determined by the MTT assay.

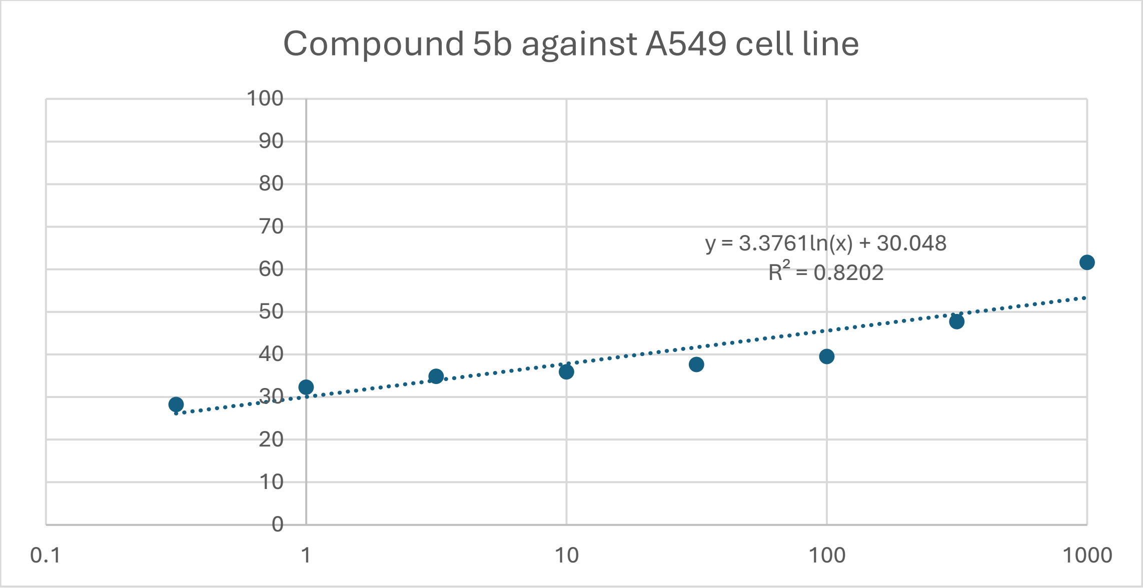

Figure S44. Dose–response curve of compound **5b** in A549 cells determined by the MTT assay.

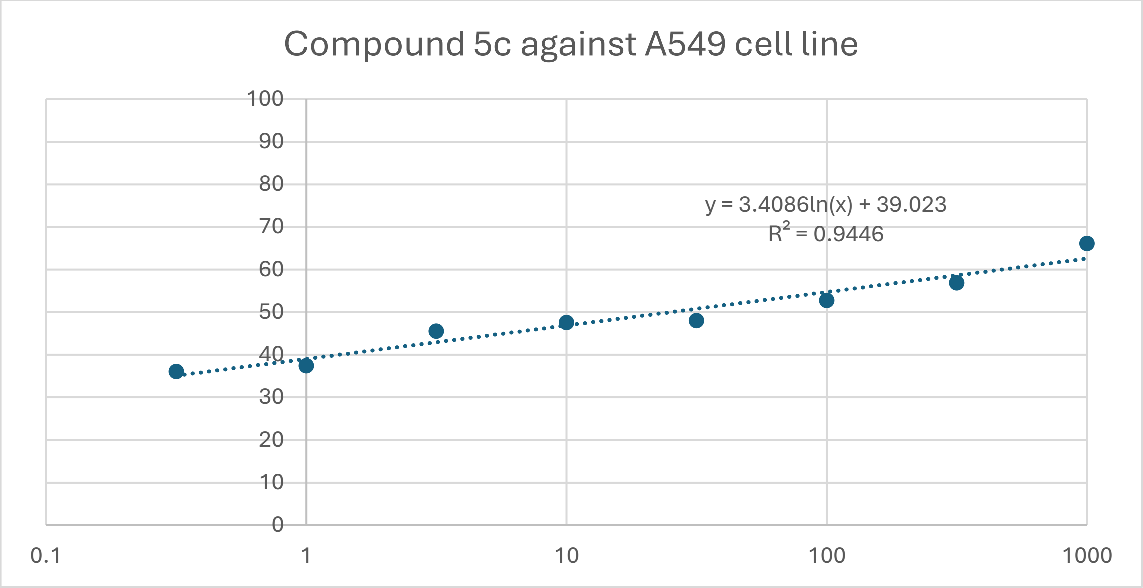

Figure S45. Dose–response curve of compound **5c** in A549 cells determined by the MTT assay.

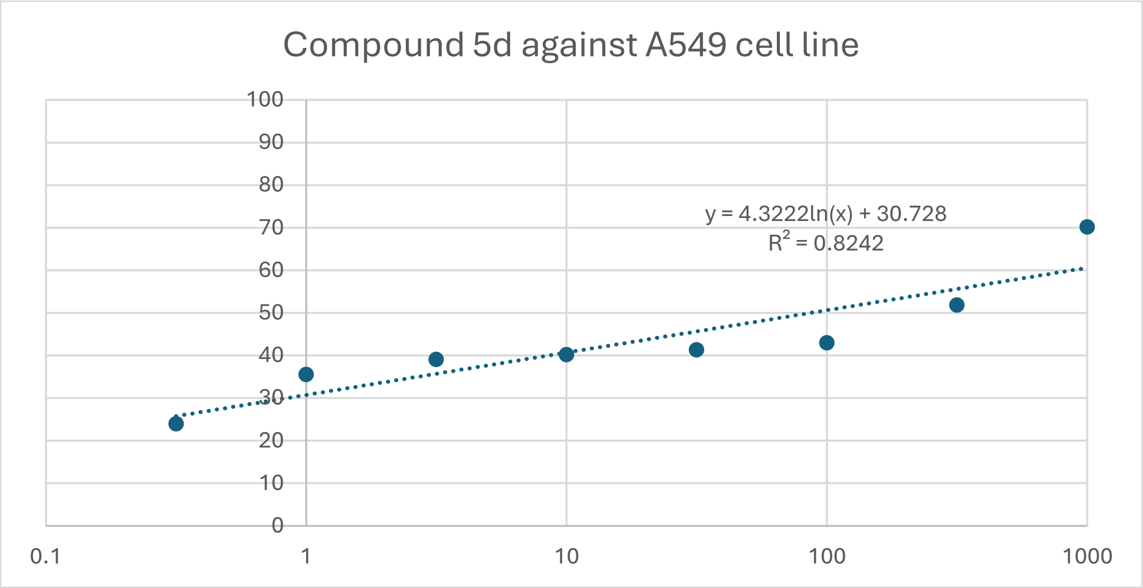

Figure S46. Dose–response curve of compound **5d** in A549 cells determined by the MTT assay.

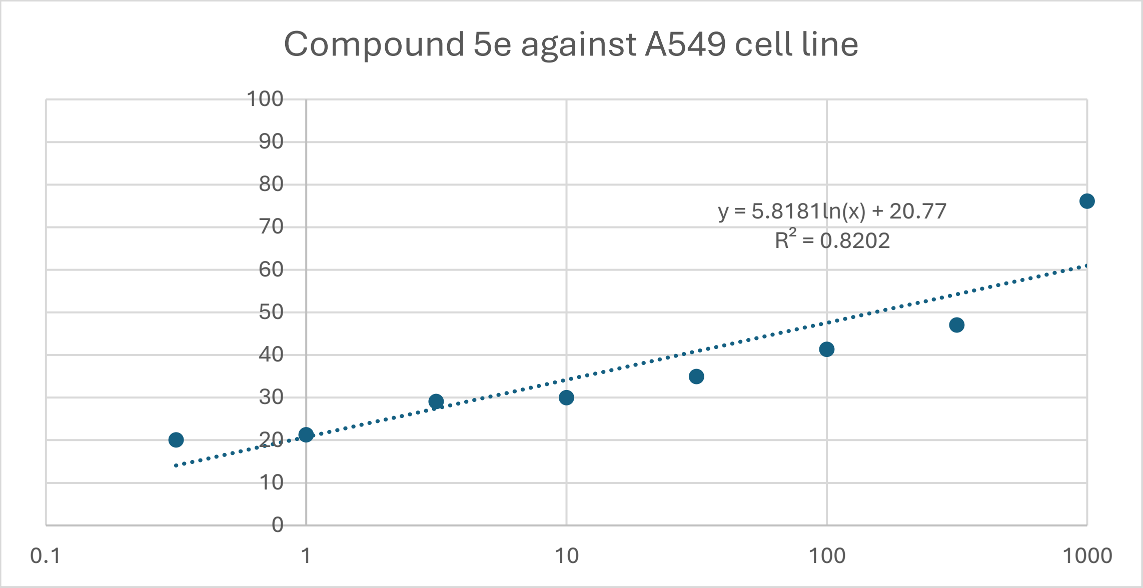

Figure S47. Dose–response curve of compound **5e** in A549 cells determined by the MTT assay.

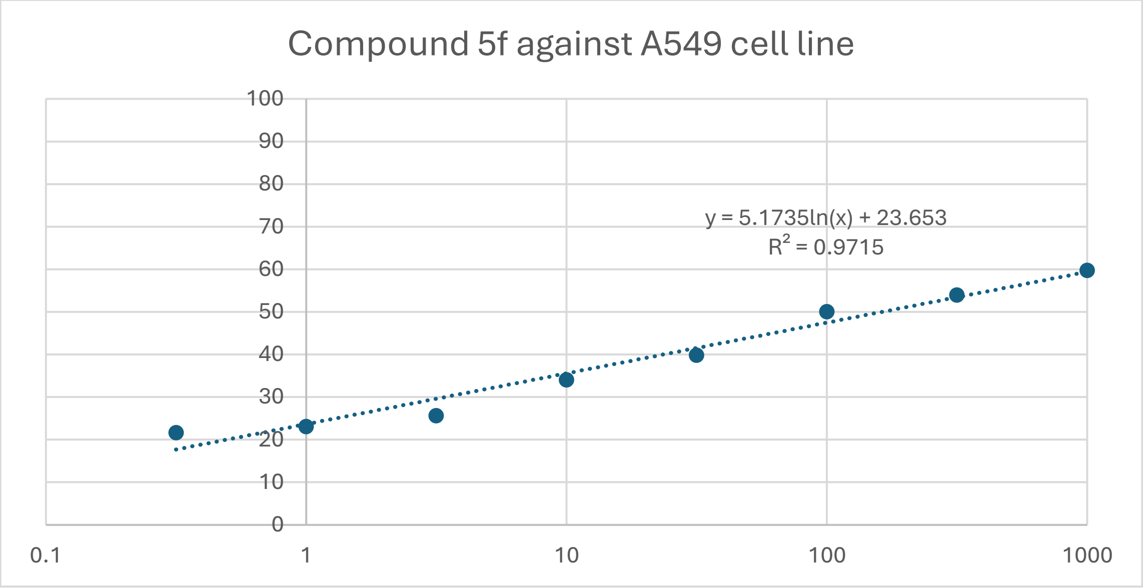

Figure S48. Dose–response curve of compound **5f** in A549 cells determined by the MTT assay.

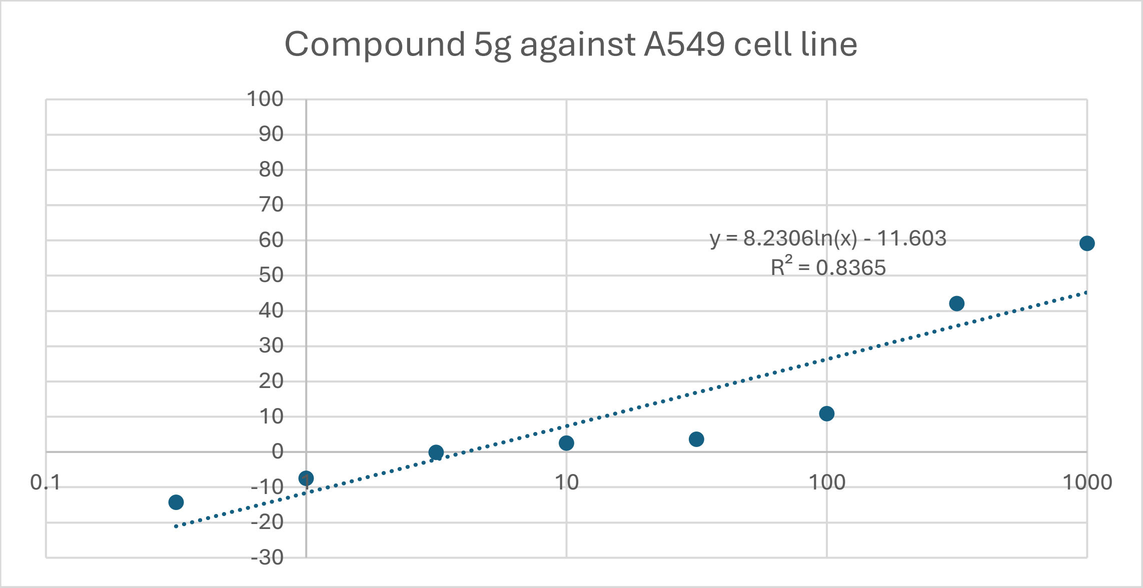

Figure S49. Dose–response curve of compound **5g** in A549 cells determined by the MTT assay.

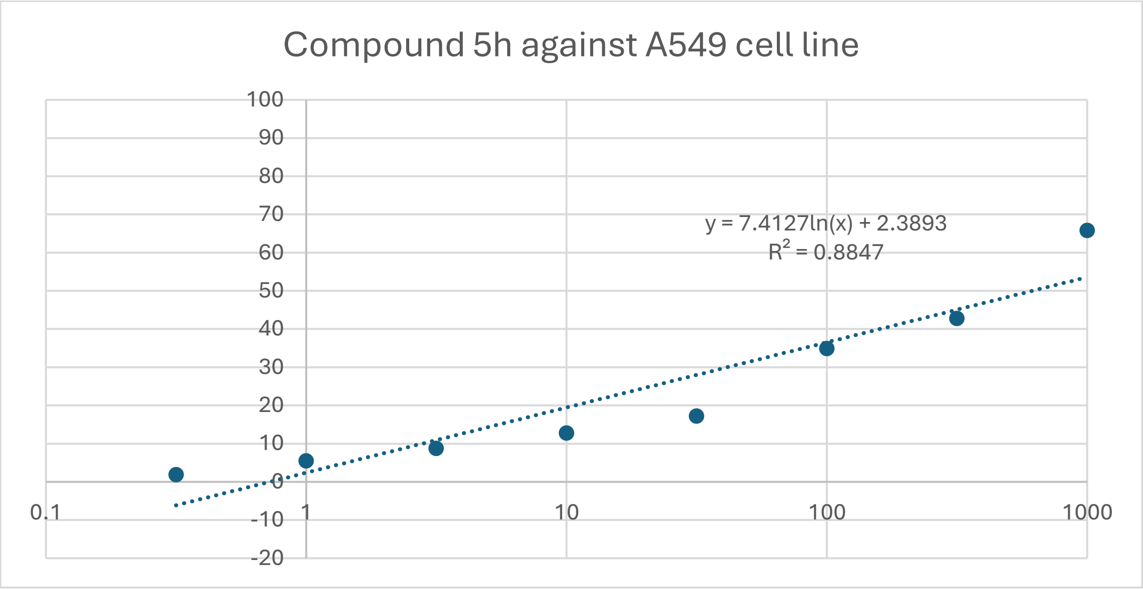

Figure S50. Dose–response curve of compound **5h** in A549 cells determined by the MTT assay.

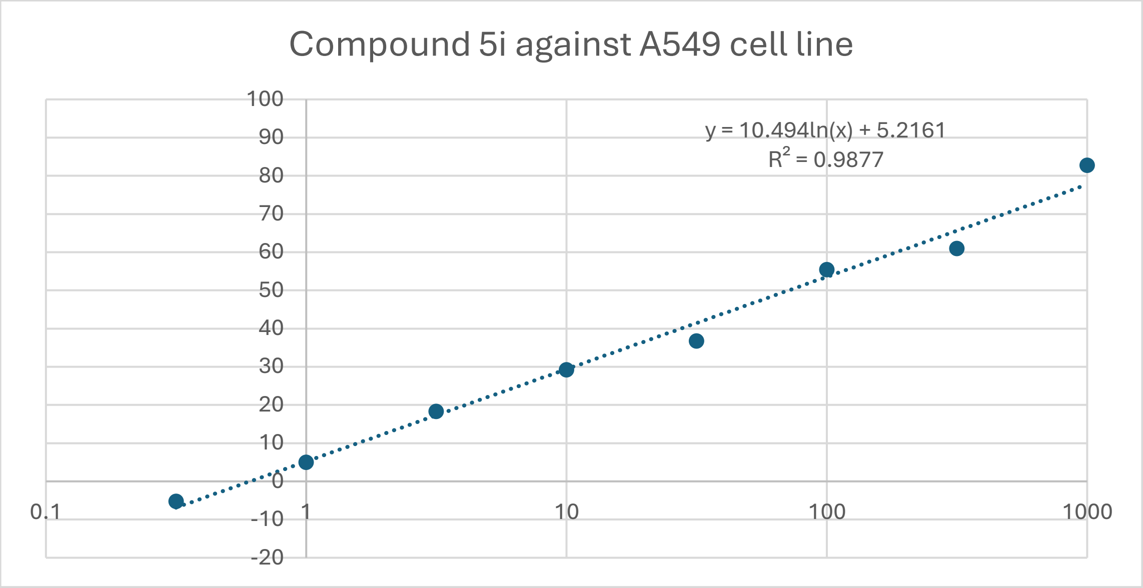

Figure S51. Dose–response curve of compound **5i** in A549 cells determined by the MTT assay.

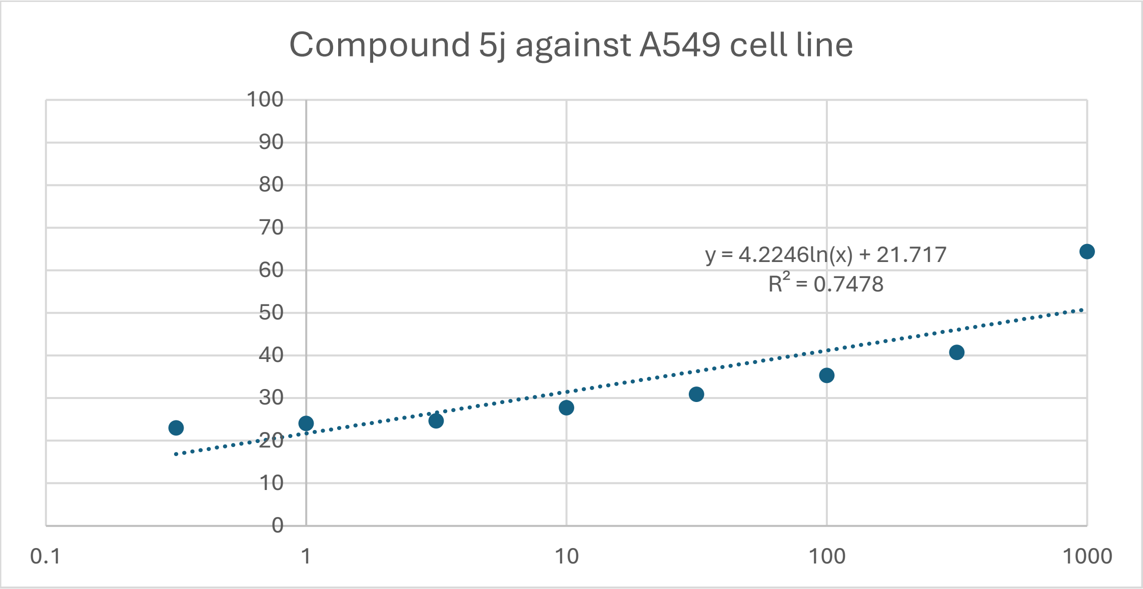

Figure S52. Dose–response curve of compound **5j** in A549 cells determined by the MTT assay.

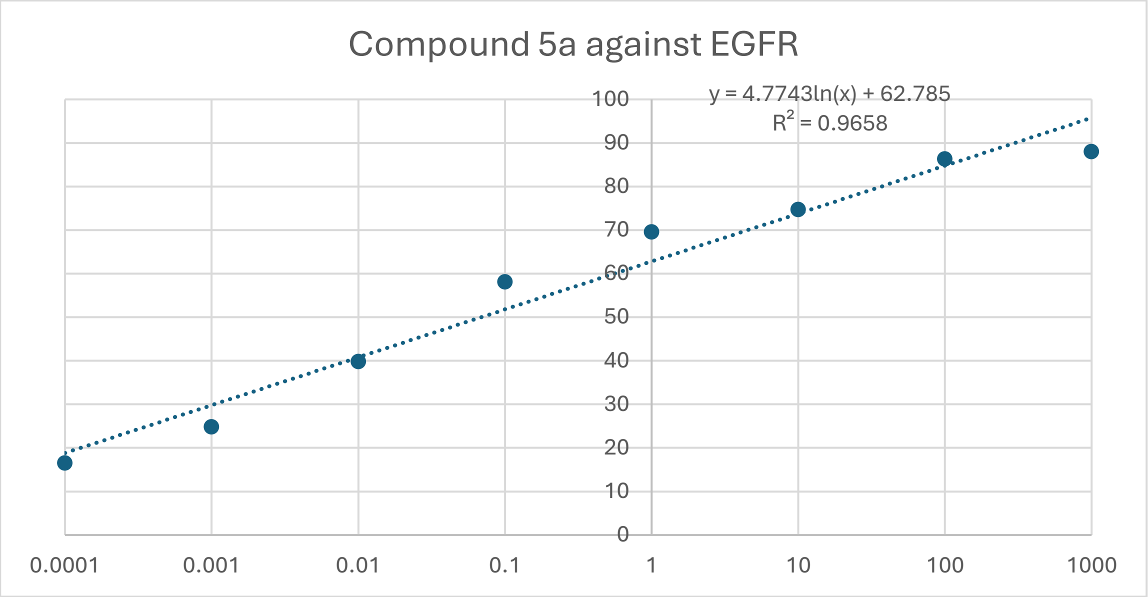

Figure S53. Dose–response curve of compound **5a** against EGFR enzyme.

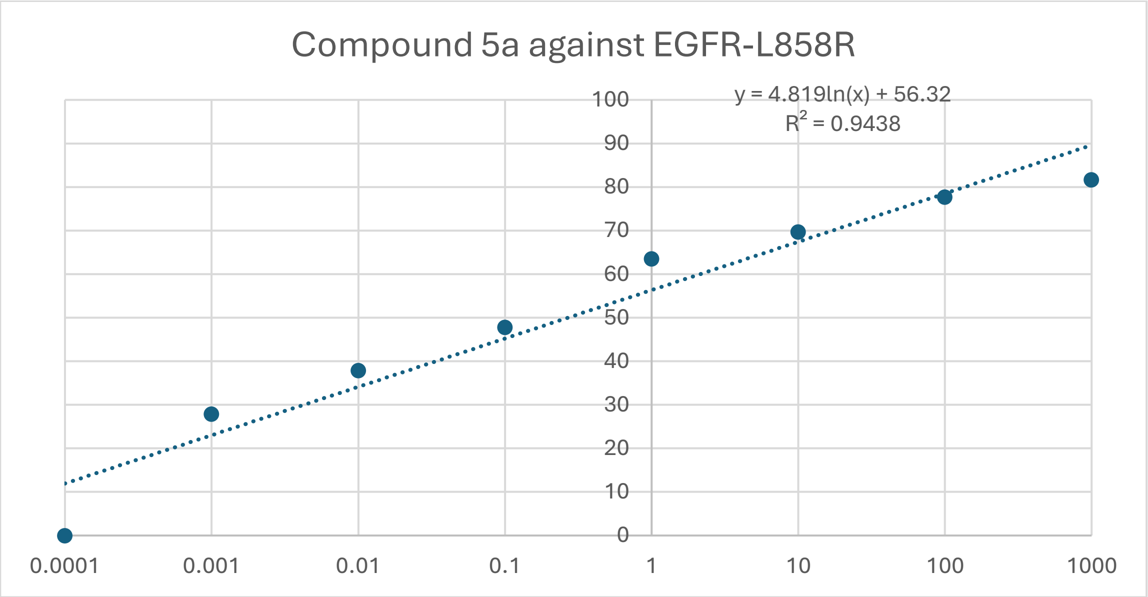

Figure S54. Dose–response curve of compound **5a** against EGFR-L858R enzyme.

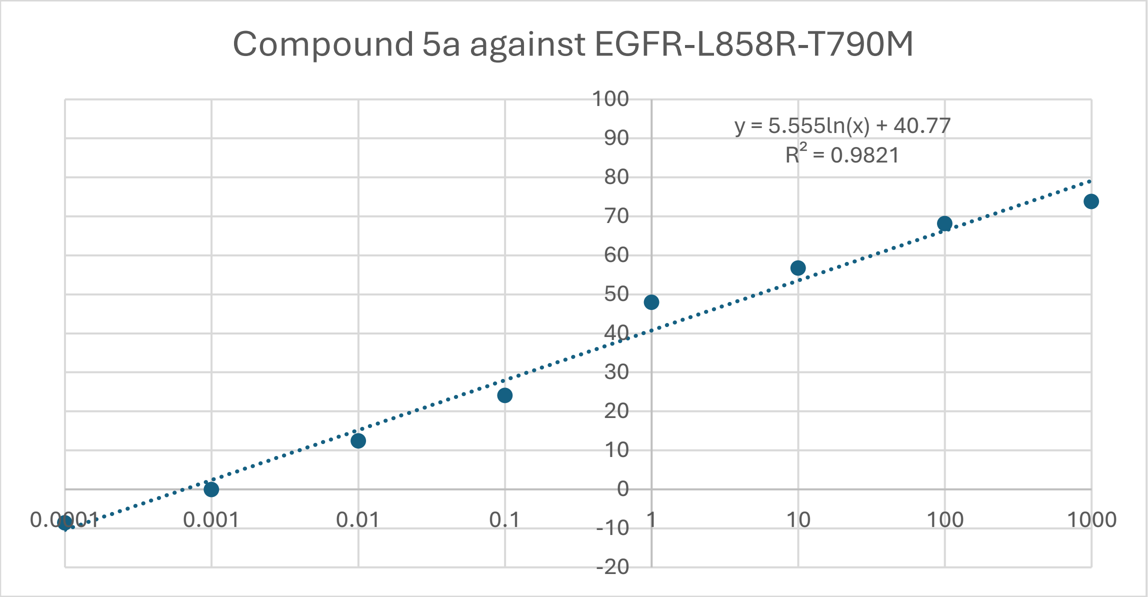

Figure S55. Dose–response curve of compound **5a** against EGFR-L858R-T790M enzyme.

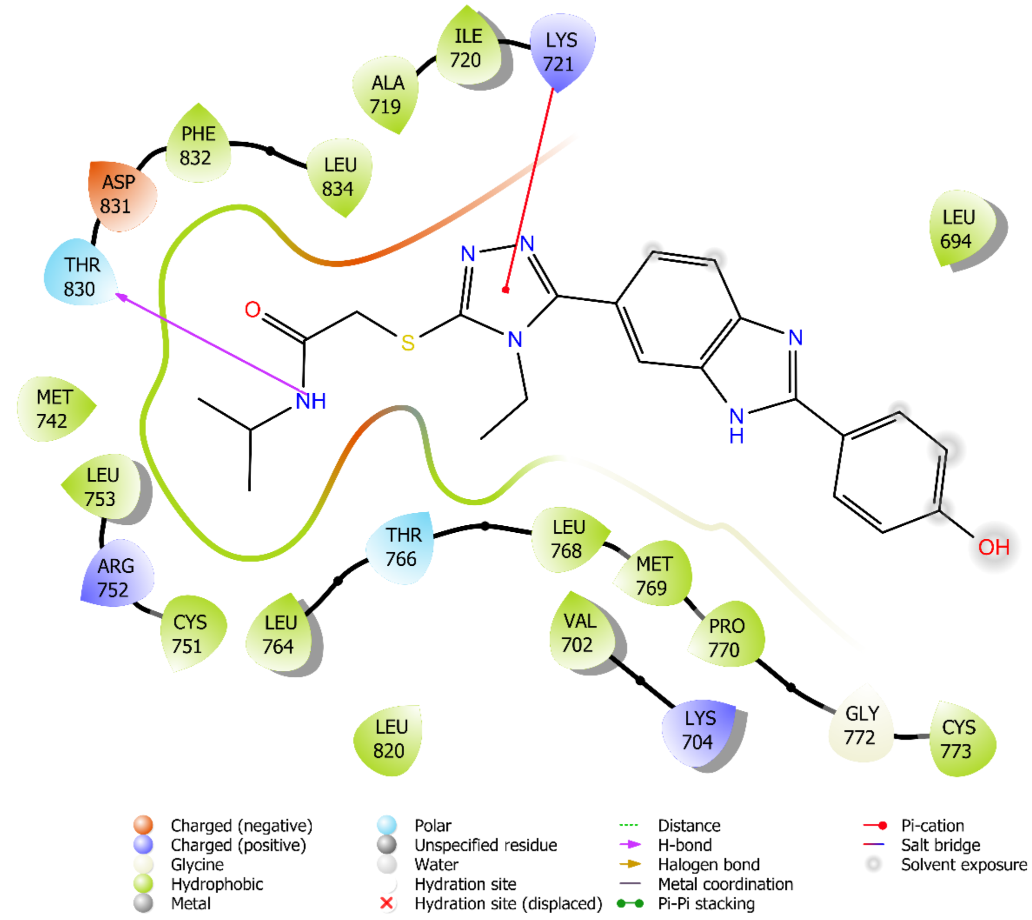

Figure S56. 2D pose of compound **5b** with EGFR enzyme active site (PDB ID:4HJO).

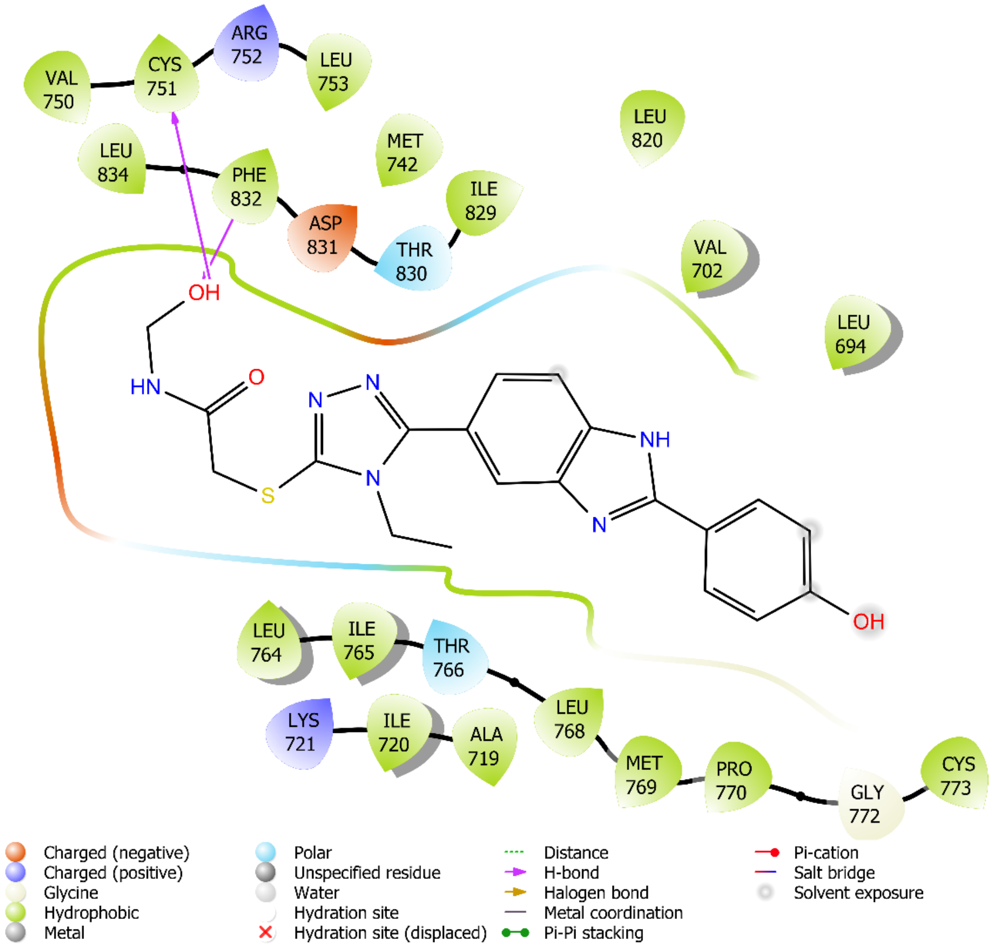

Figure S57. 2D pose of compound **5c** with EGFR enzyme active site (PDB ID:4HJO).

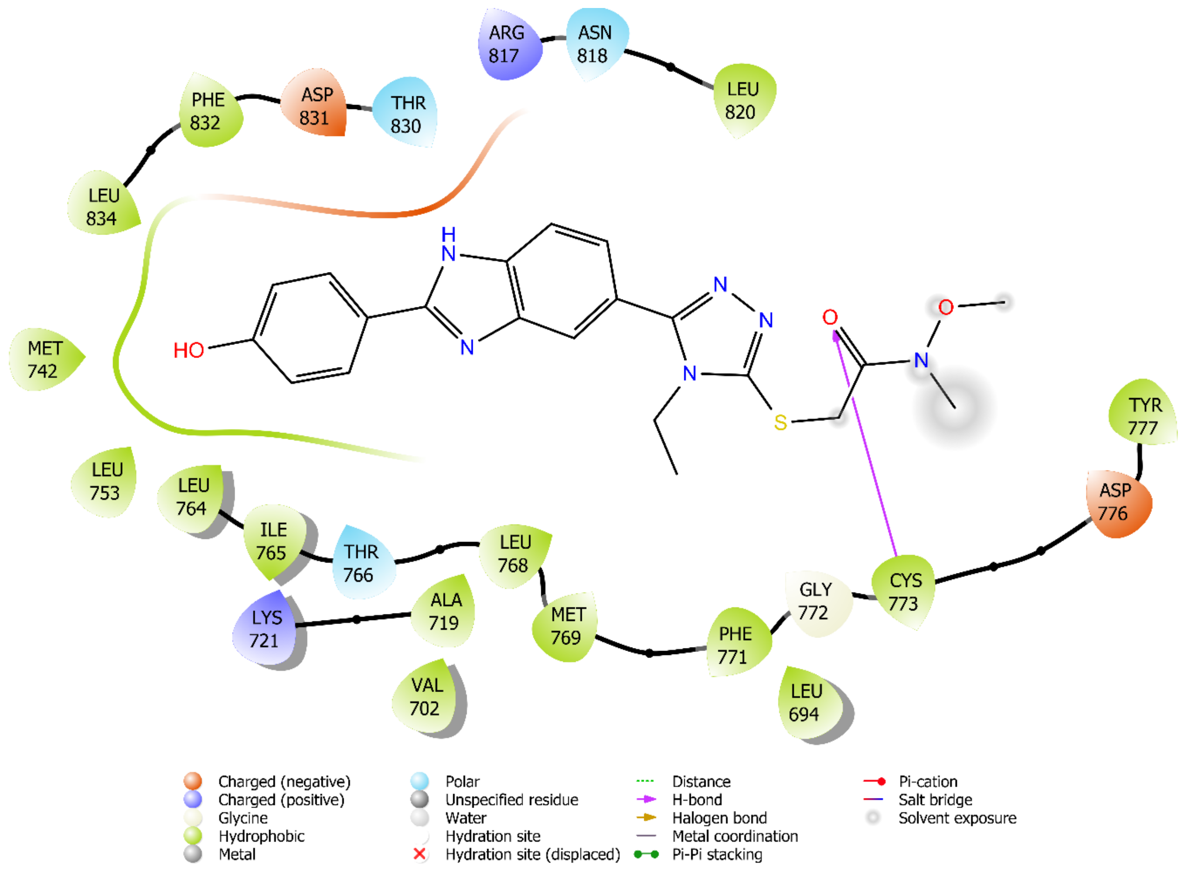

Figure S58. 2D pose of compound **5d** with EGFR enzyme active site (PDB ID:4HJO).

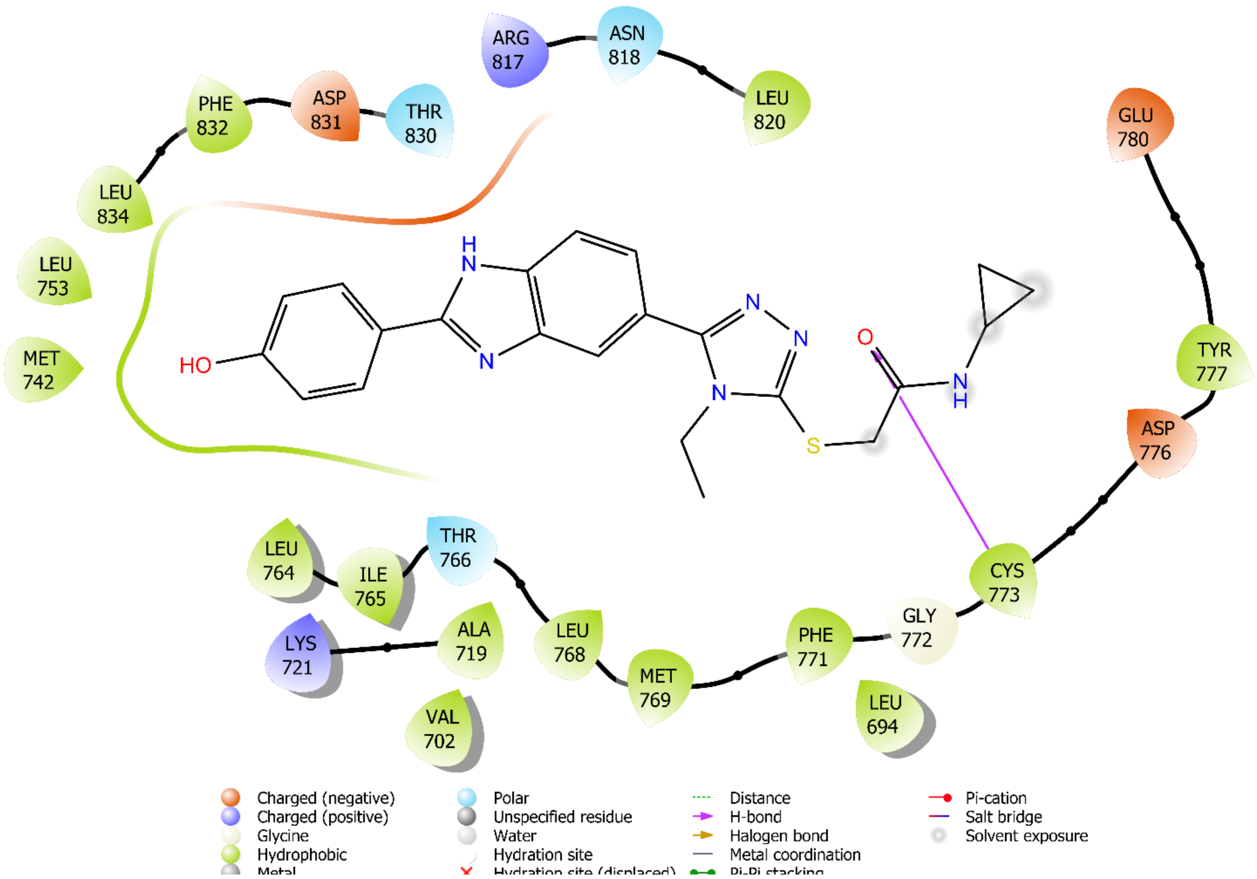

Figure S59. 2D pose of compound **5e** with EGFR enzyme active site (PDB ID:4HJO).

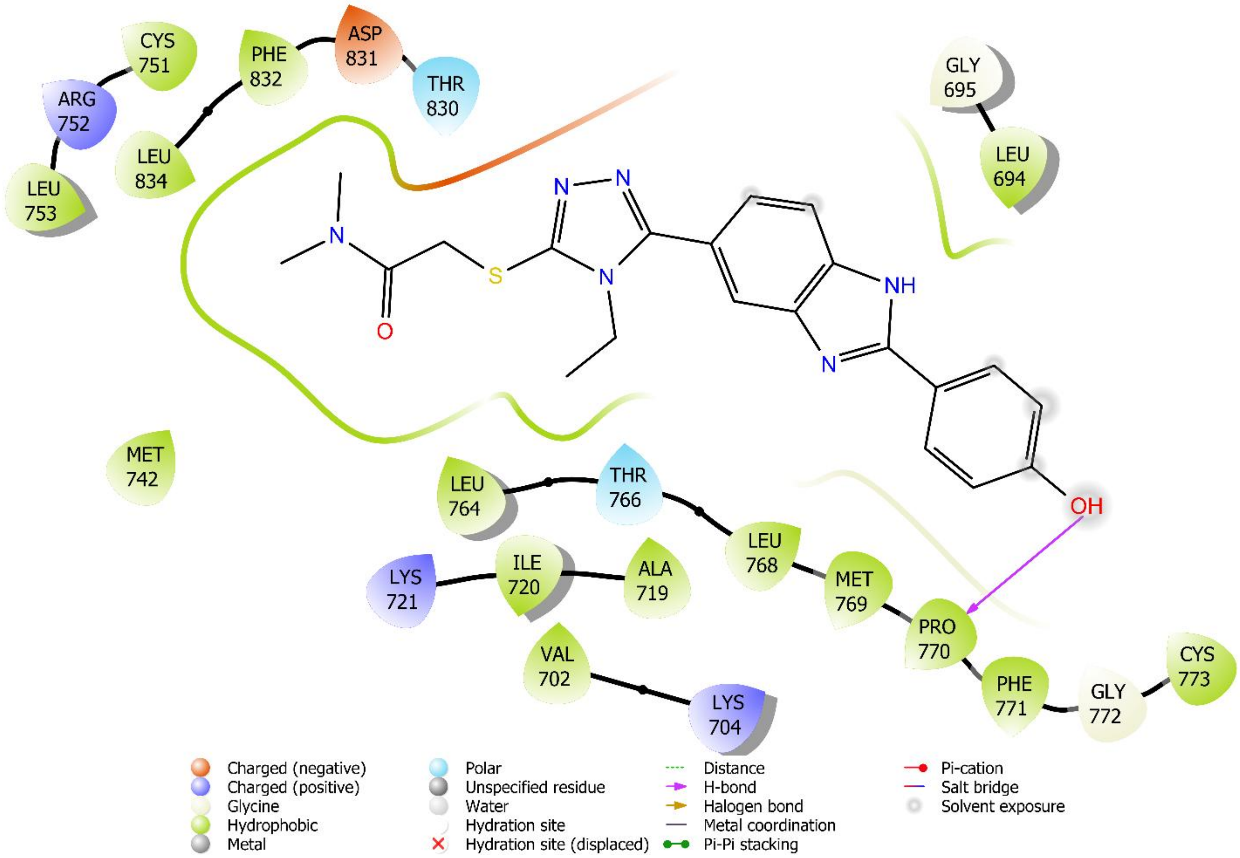

Figure S60. 2D pose of compound **5f** with EGFR enzyme active site (PDB ID:4HJO).

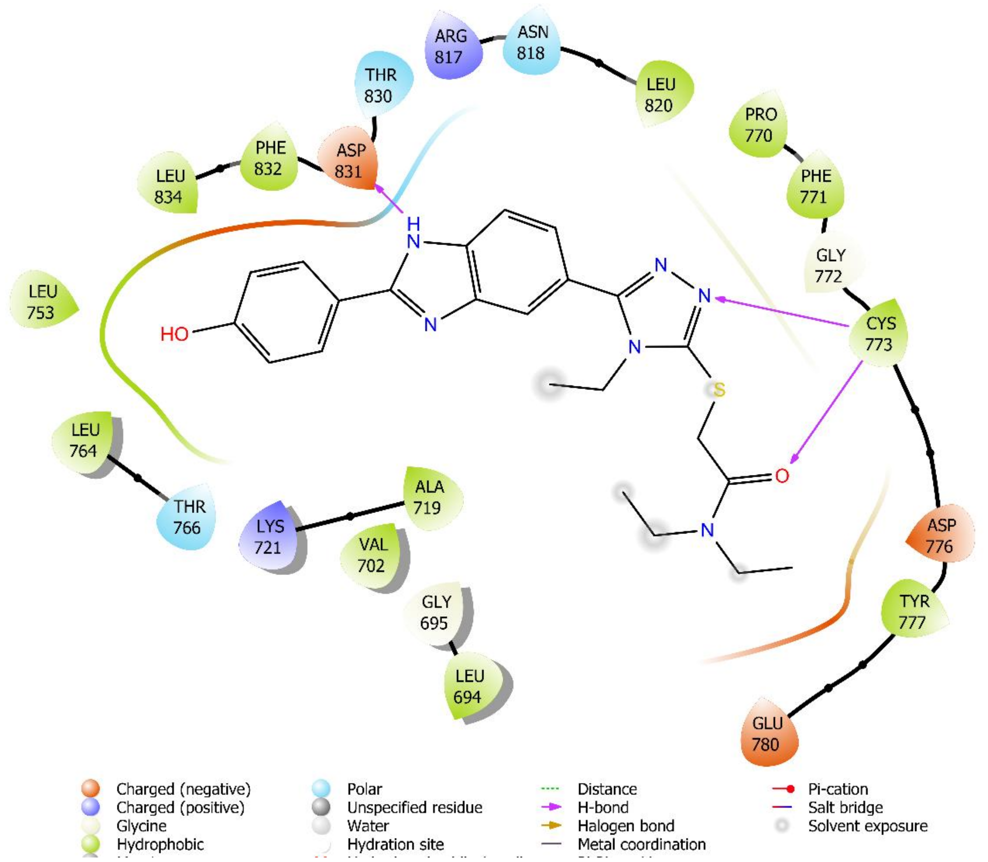

Figure S61. 2D pose of compound **5g** with EGFR enzyme active site (PDB ID:4HJO).

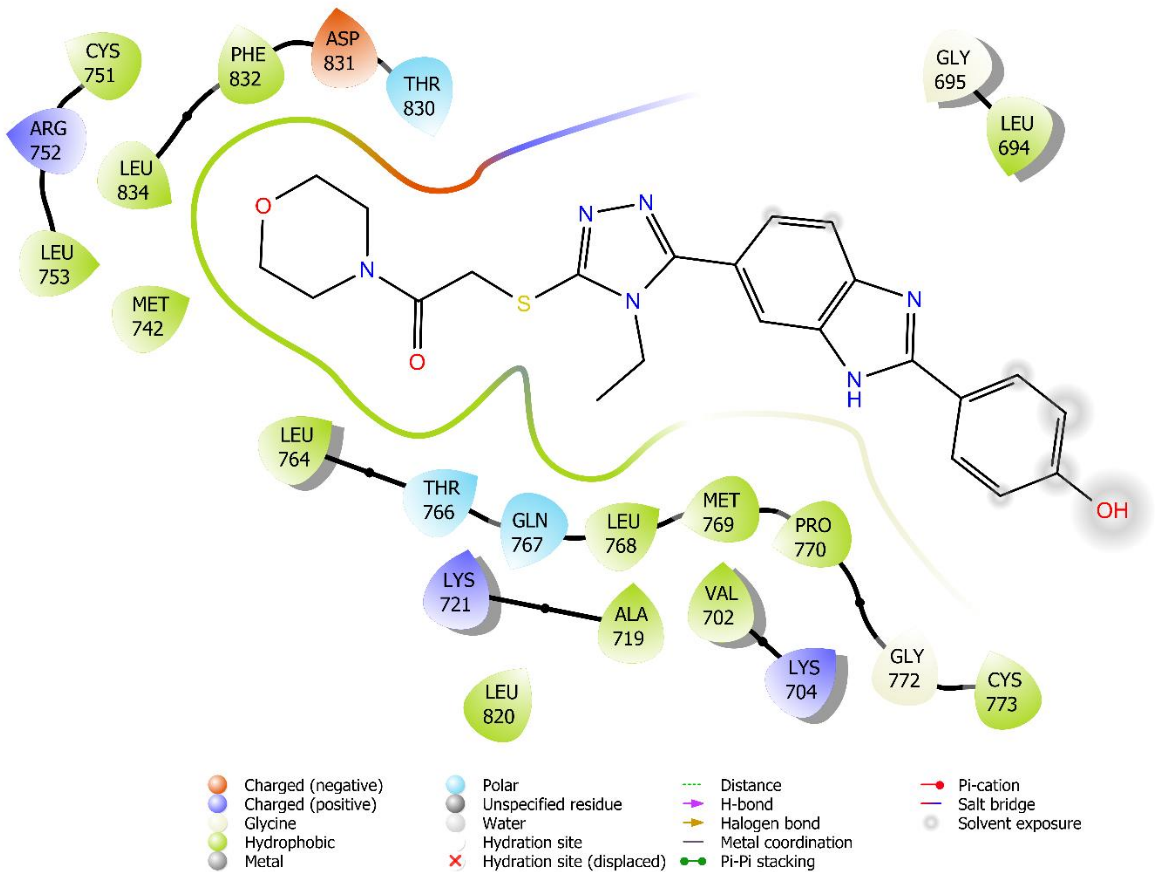

Figure S62. 2D pose of compound **5h** with EGFR enzyme active site (PDB ID:4HJO).

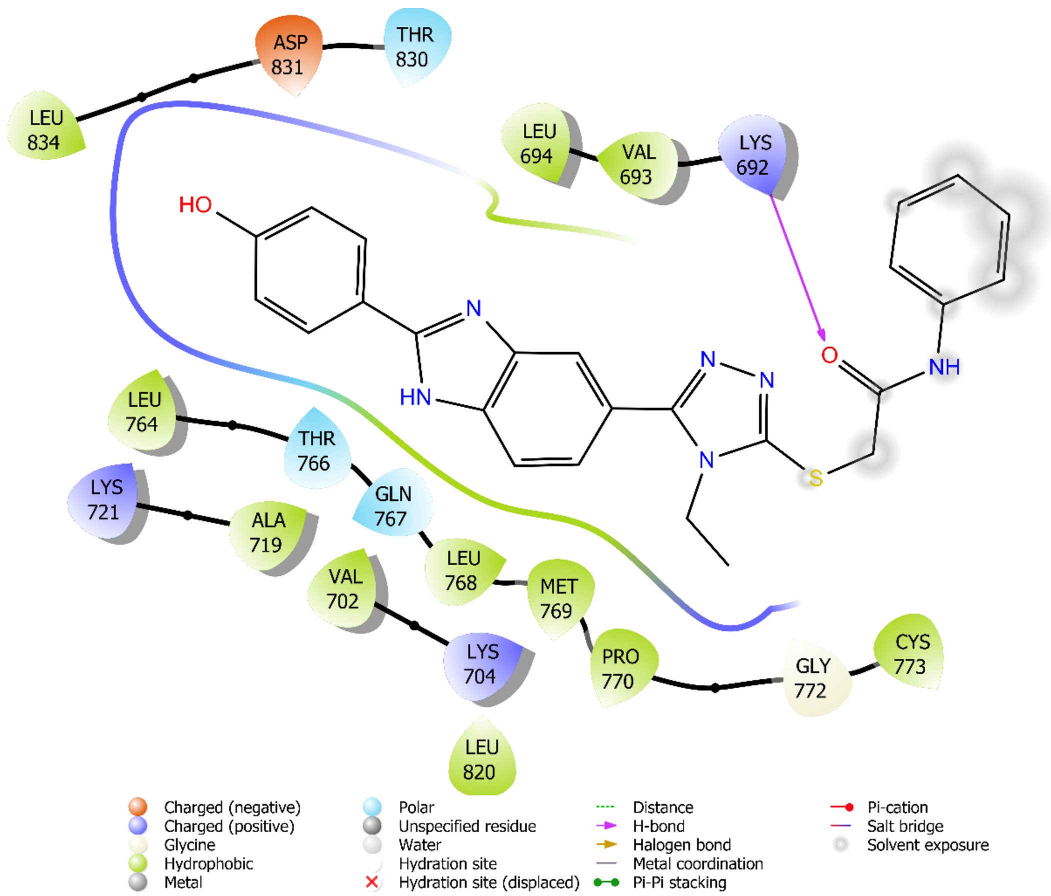

Figure S63. 2D pose of compound **5i** with EGFR enzyme active site (PDB ID:4HJO).

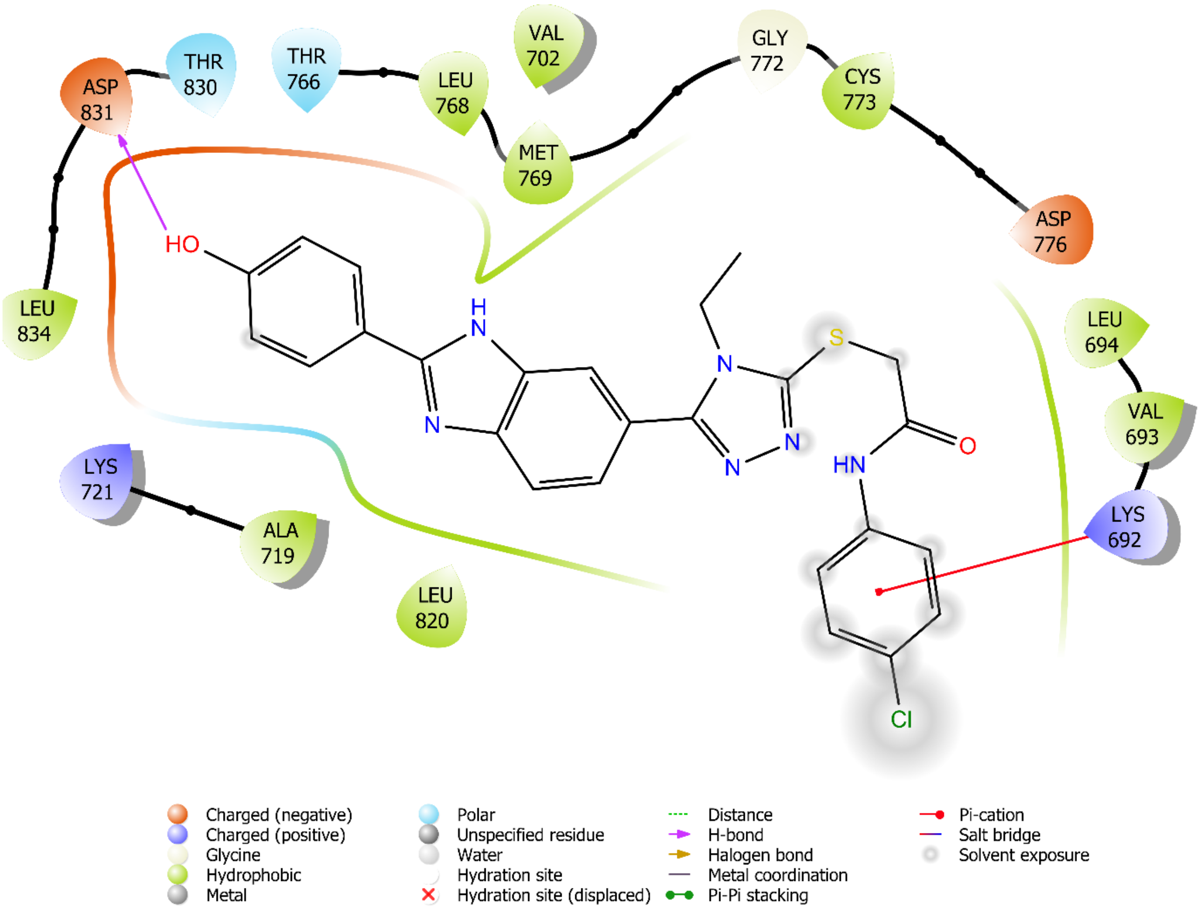

Figure S64. 2D pose of compound **5j** with EGFR enzyme active site (PDB ID:4HJO).

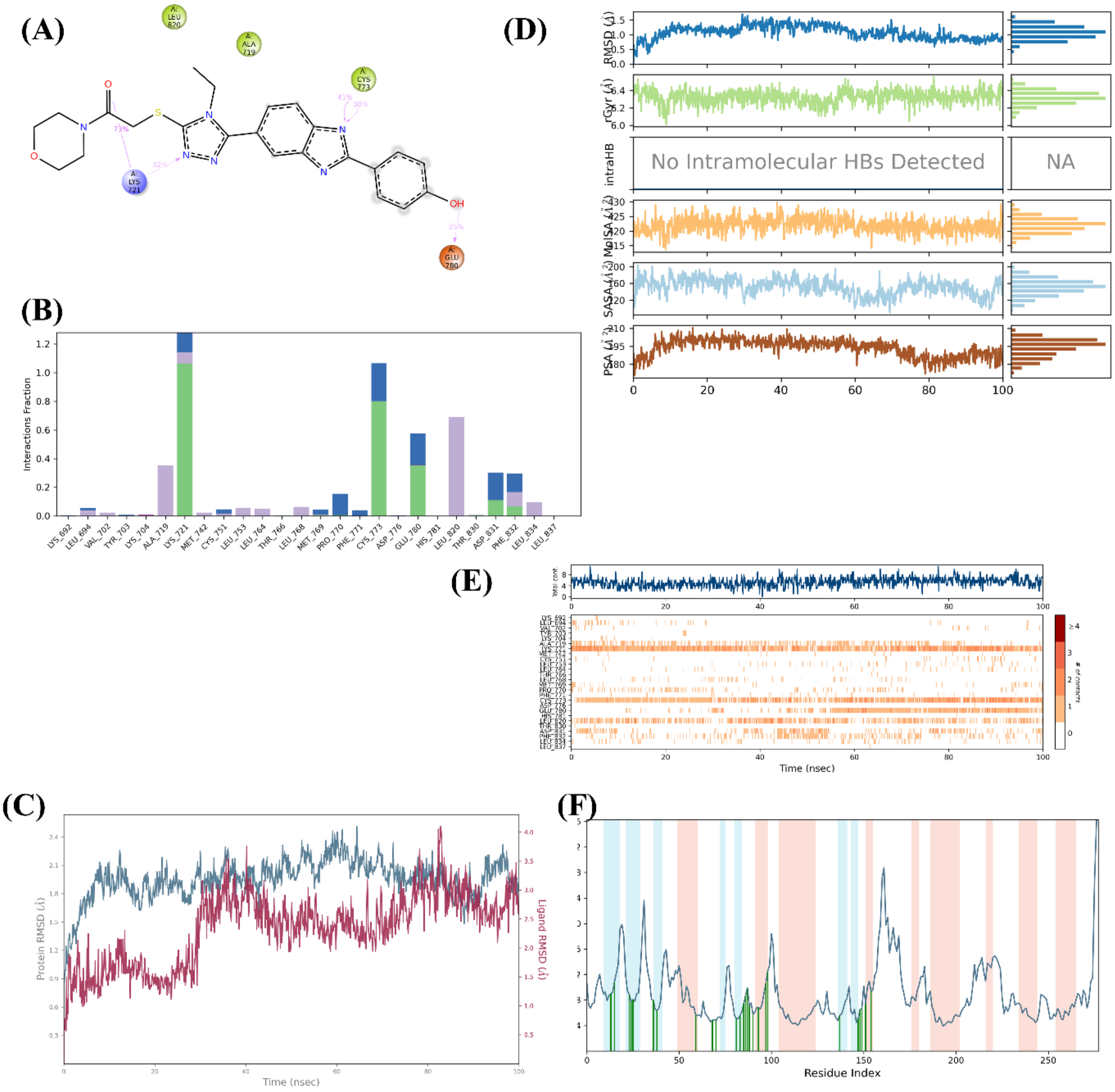

Figure S65. Molecular dynamic studies for compound **5i.**

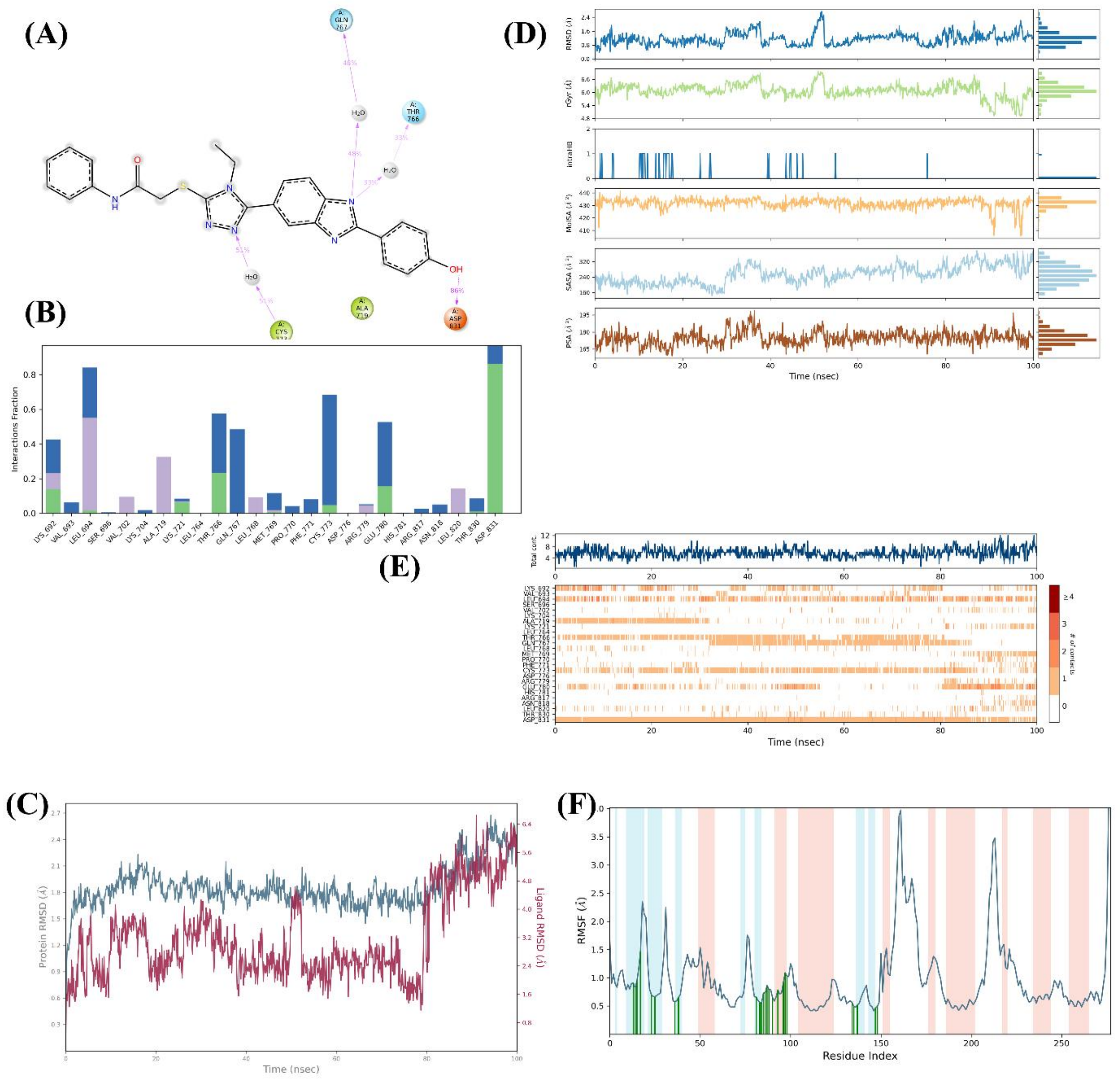

Figure S66. Molecular dynamic studies for compound **5j.**
